# Discovery of YJZ5118: a Potent and Highly Selective Irreversible CDK12/13 Inhibitor with Synergistic Effects in Combination with Akt Inhibition

**DOI:** 10.1021/acs.jmedchem.5c00127

**Published:** 2025-03-13

**Authors:** Jianzhang Yang, Yu Chang, Kaijie Zhou, Weixue Huang, Jean Ching-Yi Tien, Pujuan Zhang, Wenyan Liu, Licheng Zhou, Yang Zhou, Xiaomei Ren, Rahul Mannan, Somnath Mahapatra, Yuping Zhang, Rudana Hamadeh, Grafton Ervine, Zhen Wang, George Xiaoju Wang, Arul M. Chinnaiyan, Ke Ding

**Affiliations:** α State Key Laboratory of Chemical Biology, Shanghai Institute of Organic Chemistry, Chinese Academy of Sciences, #345 Lingling Road, Shanghai 200032, China; β International Cooperative Laboratory of Traditional Chinese Medicine Modernization and Innovative Drug Discovery of Chinese Ministry of Education (MOE), Guangzhou City Key Laboratory of Precision Chemical Drug Development, College of Pharmacy, Jinan University, 855 Xingye Avenue East, Guangzhou 511400, China; δ Michigan Center for Translational Pathology, University of Michigan, Ann Arbor, Michigan 48109, USA; Ψ Department of Pathology, University of Michigan, Ann Arbor, MI 48109, USA; Φ Howard Hughes Medical Institute, University of Michigan, Ann Arbor, MI 48109, USA; χ Rogel Cancer Center, University of Michigan, Ann Arbor, MI 48109, USA; ξ Department of Urology, University of Michigan, Ann Arbor, MI 48109, USA

**Keywords:** CDK12, CDK13, covalent inhibitor, castration-resistant prostate cancer, synergistic effect with Akt inhibitors

## Abstract

Cyclin-dependent kinases 12 and 13 (CDK12/13) have emerged as promising therapeutic targets for castration-resistant prostate cancer (CRPC) and other human cancers. Despite the development of several CDK12/13 inhibitors, challenges remain in achieving an optimal balance of potency, selectivity and pharmacokinetic properties. Here, we report the discovery of **YJZ5118**, a novel, potent and highly selective covalent inhibitor of CDK12/13 with reasonable pharmacokinetic profiles. **YJZ5118** effectively inhibited CDK12 and CDK13 with IC_50_ values of 39.5 and 26.4 nM, respectively, while demonstrating high selectivity over other CDKs. Mass spectrometry analysis, co-crystal structure determination, and pulldown-proteomic experiments confirmed the compound’s covalent binding mode with CDK12/13. Functionally, **YJZ5118** efficiently suppressed the transcription of DNA damage response genes, induced DNA damage, and triggered apoptosis. Moreover, the compound significantly inhibited the proliferation of multiple tumor cell lines, particularly prostate cancer cells. Notably, **YJZ5118** exhibited synergistic effects with Akt inhibitors both *in vitro* and *in vivo*.

## Graphical Abstract

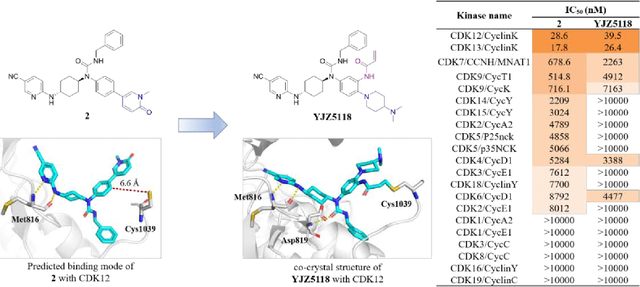

## INTRODUCTION

Cyclin-dependent kinase 12 (CDK12) and its paralog CDK13 belong to a transcription-associated CDK family,^1^ and play indispensable roles in DNA damage response (DDR) and the maintenance of genomic stability.^2, 3^ These kinases cooperatively regulate transcription elongation, splicing, and cleavage and polyadenylation by triggering phosphorylation of serine 2 in the C-terminal domain (CTD) of RNA polymerase II in complex with their essential partner protein, cyclin K (CCNK).^4^ CDK12/13 have emerged as promising therapeutic targets for various human cancers,^5, 6^ as evidenced by the inhibition of cell proliferation following genetic knockdown or pharmacological inhibition/degradation of these targets in both triple-negative breast cancer (TNBC) and castration-resistant prostate cancer (CRPC) cells.^7–11^ Furthermore, CDK12 mutations have been identified in 5%–7% of patients with metastatic CRPC (mCRPC).^10–12^ Our recent studies have also suggested that CDK12 mutation may serve as synergistic biomarkers for CDK12/13 proteolysis-targeting chimeras (PROTACs) in mCRPC cells.^13, 14^

**Figure 1.**
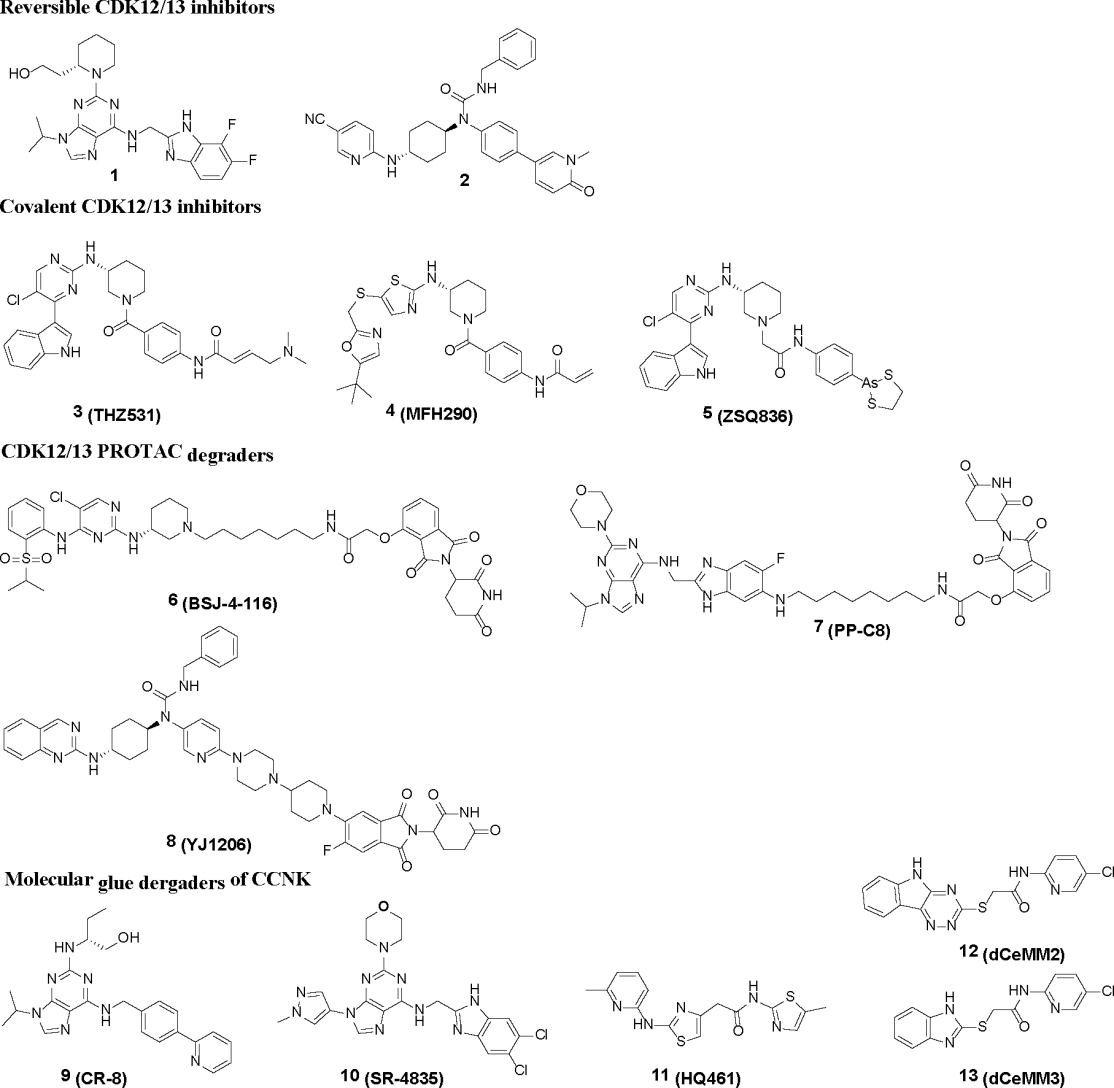
Representative compounds suppressing/degrading CDK12/13 kinases.

Multiple approaches are being explored to selectively suppress CDK12/13, including kinase function inhibition (*e.g.*, **1-5**),^7, 15–19^ PROTAC degradation (*e.g.*, **6-8**) of CDK12/13,^14, 20, 21^ and molecular glues degrading the partner protein CCNK (*e.g.*, **9-13**)^22–26^ (Figure 1). Most recently, Carrick Therapeutics initiated a phase 1 clinical trial for CT7439, a novel CCNK degrader.^27^ Additionally, a derivative of **YJ1206**, the 1^st^ reported orally bioavailable CDK12/13 degrader, was nominated as a potential clinical candidate for further development in our team.^14^ Although several reversible or irreversible CDK12/13 inhibitors have been reported,^7, 15–19^ none have progressed into clinical trials, likely due to the challenges in balancing antiproliferative activity, target selectivity, pharmacokinetic (PK) profiles and/or safety concerns. Moreover, there is limited *in vivo* efficacy data for these inhibitors.^7, 19^

SR-4835 (**10**) is the 1^st^ selective CDK12/13 inhibitor to demonstrate *in vivo* therapeutic efficacy in TNBC models; however, subsequent studies suggest that its activity may be attributed to CCNK degradation.^23, 24^ Interestingly, reversible inhibitors (*e.g.* **1**, **2**) generally exhibit significantly weaker antiproliferative activity compared to covalent inhibitors (*e.g.* **3-5**), despite their comparable kinase suppressive potency. This observation highlights the potential advantage of irreversible CDK12/13 inhibitors, such as prolonged target engagement and enhanced ATP-competitive binding within cells.^28, 29^ Additionally, the distinct cysteine residues near the ATP-binding pockets of CDK12 (Cys1039) and CDK13 (Cys1017),^17^ present an opportunity to improve target specificity through covalently targeting. Notably, most current covalent CDK12/13 inhibitors are structurally derived from the CDK7 covalent inhibitor THZ1, which targets a similar cysteine residue in the structure (Cys312).^17, 30^ Developing new irreversible inhibitors with alternative chemical scaffolds is highly desirable for further validating selective CDK12/13 inhibition as a viable cancer therapeutic strategy.

Herein, we report the discovery of **YJZ5118**, a new, potent, and highly selective covalent inhibitor of CDK12/13, by optimization of a structurally distinct reversible inhibitor **2**. **YJZ5118** effectively inhibited CDK12 and CDK13 with low nanomolar IC_50_ values, demonstrating improved target specificity. The covalent binding mode of **YJZ5118** was extensively validated through mass spectrometry analysis, co-crystal structure determination, and pulldown-proteomic experiments. Furthermore, the compound exhibited reasonable PK parameters and demonstrated promising *in vivo* efficacy in a VCaP CRPC xenograft mouse model when combined with Akt inhibitors.

## RESULTS AND DISCUSSION

### Molecule Design.

**Figure 2.**
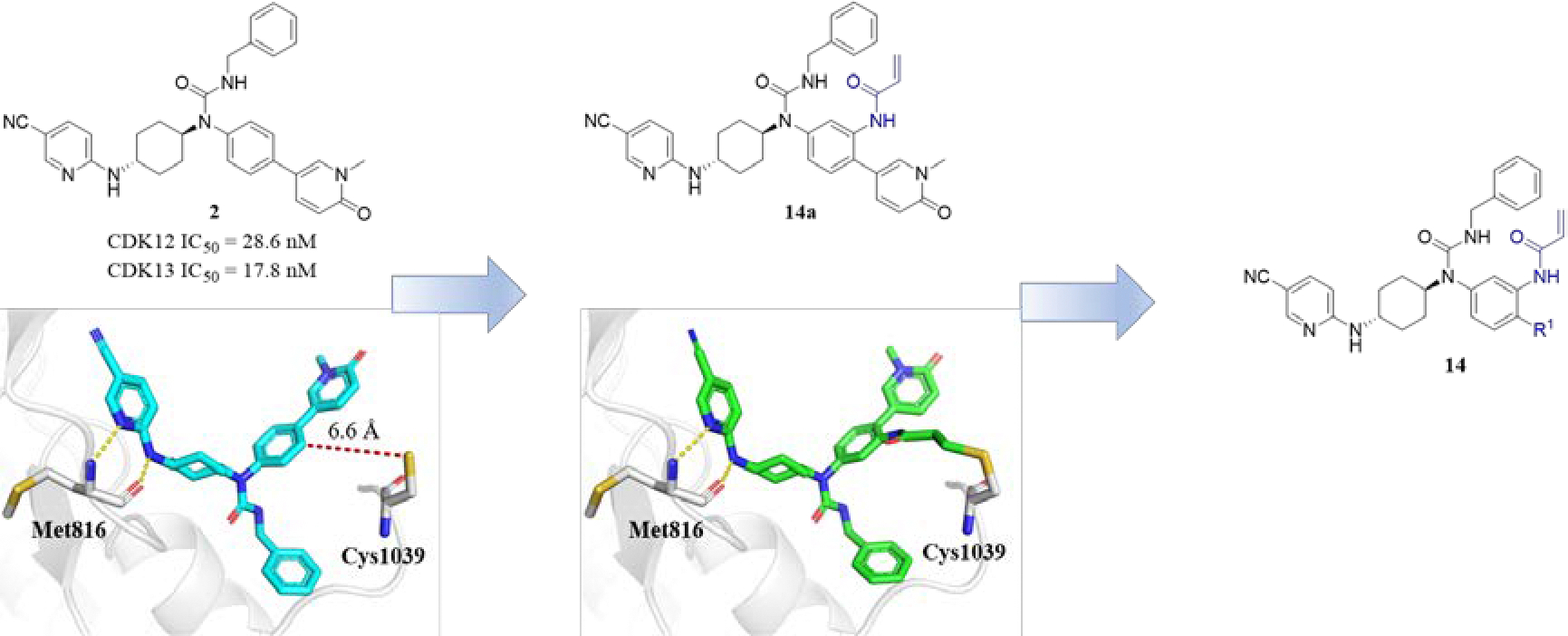
Structure-based design of new irreversible CDK12/13 inhibitors **11**. Chemical structures and predicted binding modes of compounds **2** and **14a** with CDK12 (PDB: 5ACB) were shown. Hydrogen bonds were indicated by yellow dashed lines.

**Figure 3.**
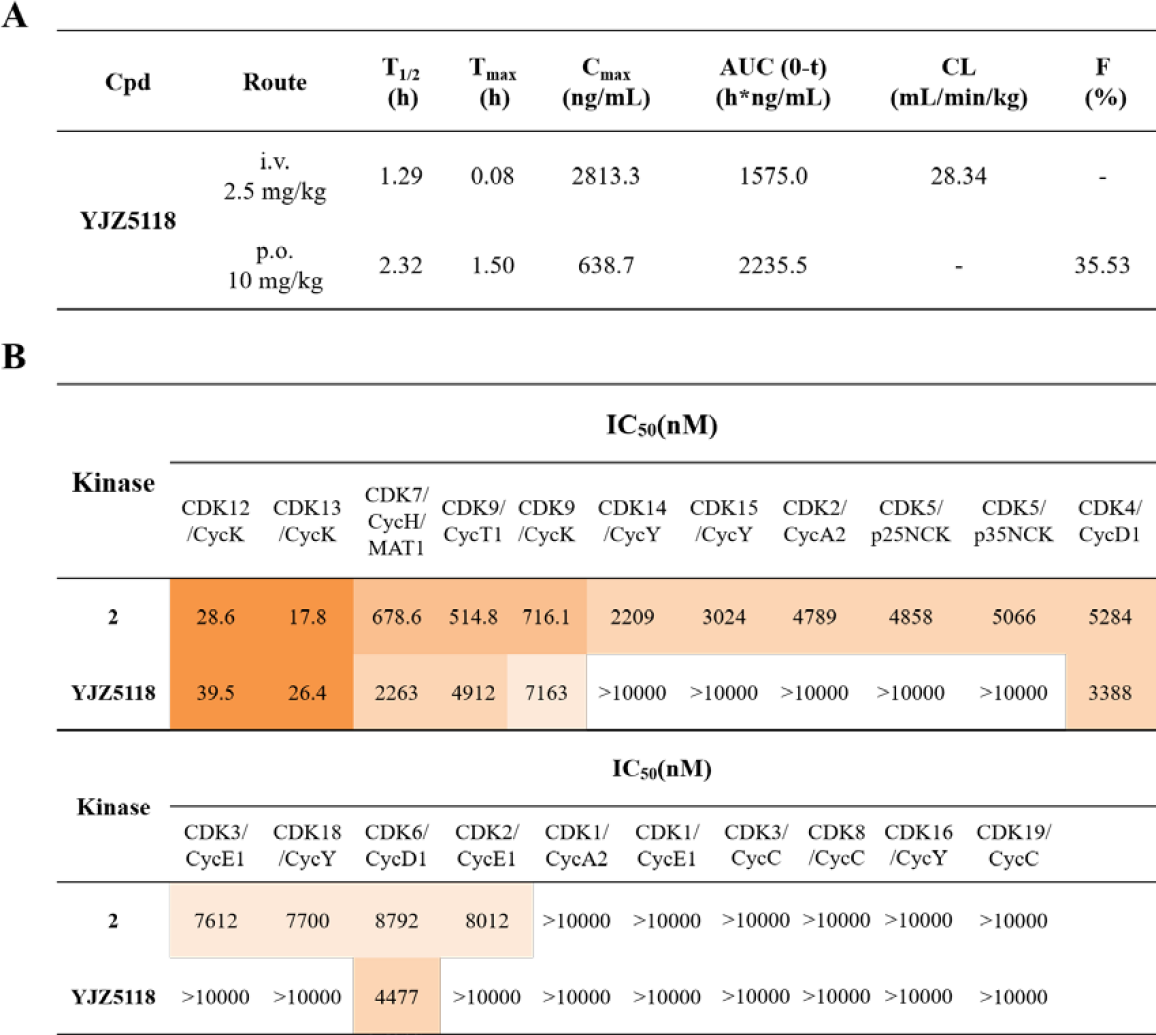
(A) PK profiles of compound **YJZ5118** in mice; (B) Kinase inhibitory IC_50_ values of compounds **2** and **YJZ5118** against CDK family members.

Compound **2** is a 3-benzyl-1-(trans-4-((5-cyanopyridin-2-yl)amino)cyclohexyl)-1-arylurea-based CDK12/13 inhibitor with strong enzymatic inhibitory activity and excellent kinase selectivity.^16^ It effectively inhibited the kinase activities of CDK12 and CDK13 with IC_50_ values of 28.6 and 17.8 nM, respectively, in the independent ADP-Glo validation assays conducted by ICE Bioscience Inc. (Figure 2). A CDK family panel screening further validated its specificity for CDK12 and CDK13, although its selectivity over CDK7 and CDK9 was moderate, with fold-difference ranging from 18- to 40-fold (Figure 3B). The X-ray co-crystal structure of an analogue of compound **2** with CDK12/CCNK revealed detailed structural interactions (PDB: 6CKX).^16^ Combining this structural information with data from the covalent inhibitor THZ531 (**3**) (PDB: 5ACB), a computational study suggested that the aminopyridine moiety of compound **2** forms two hydrogen bonds with hinge residue Met816, while the central phenyl ring is positioned 6.6 Å from Cys1039 of CDK12. Additionally, the hydrophilic 1-methylpyridin-2(1*H*)-one group is oriented toward the solvent accessible surface. Although Cys1039 is relatively distant from the reversible inhibitor **2**, the conformational flexibility of the corresponding loop suggested the possibility of introducing an acrylamide warhead at the central phenyl ring to covalently target Cys1039 of CDK12, achieving irreversible inhibition against the kinase (**14a**). We further hypothesized that the solvent exposing 1-methylpyridin-2(1*H*)-one moiety could be diversified with basic aliphatic amines to facilitate the deprotonation of Cys1039, thereby promoting the covalent bond formation. Incorporating hydrophilic aliphatic amines was also expected to improve aqueous solubility and PK profiles (Figure 2).

### Chemistry.

**Scheme 1.**
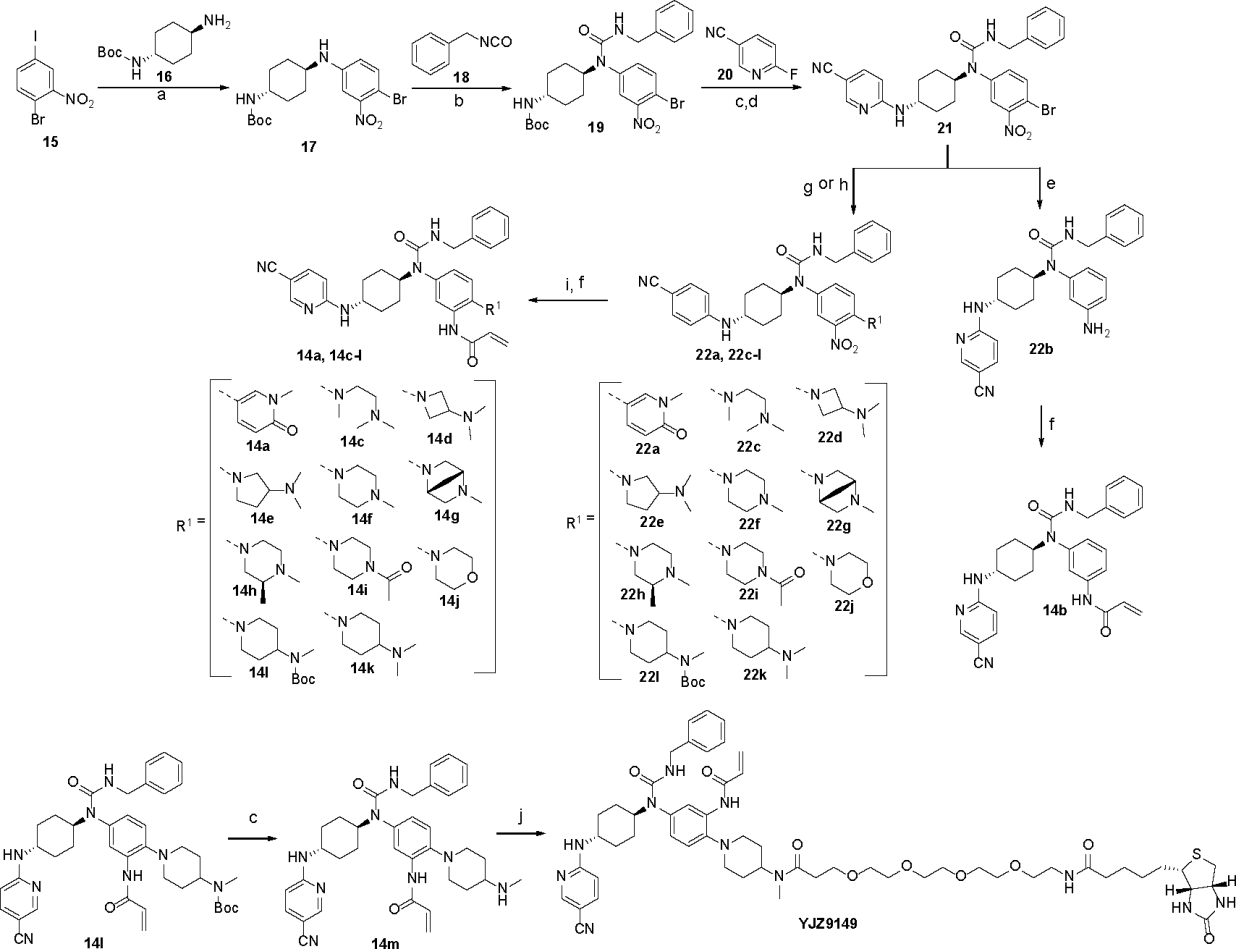
Synthesis of Compounds **14a-m** and **YJZ9149**^a^ **^*a*^Reagents and conditions:** (a) 4,5-bis(diphenylphosphino)-9,9-dimethylxanthene (Xantphos), tris(dibenzylideneacetone)dipalladium (Pd_2_(dba)_3_), *tert*-BuONa, toluene, 100 °C, 12 h, 82%; (b) *N,N*-diisopropylethylamine (DIPEA), *N,N*-dimethylformamide (DMF), 95 °C, 4 h, 79%; (c) trifluoroacetic acid (TFA), dichloromethane (DCM), 50 °C, reflux, 3 h, 60–77%; (d) Cs_2_CO_3_, DMF, 60 °C, 1 h, 80%; (e) 10% Pd-C/H_2_, MeOH, room temperature (rt), 2 h; (f) DCM, DIPEA, acryloyl chloride, 0 °C, 35–54% (two steps); (g) Xantphos, Pd_2_(dba)_3_, *tert*-BuONa, toluene, DMF, 100 °C, 40–64%; (h) 1-methyl-5-(4,4,5,5-tetramethyl-1,3,2-dioxaborolan-2-yl)pyridin-2(1*H*)-one, K_2_CO_3_, tetrakis(triphenylphosphine)palladium (Pd(PPh_3_)_4_), 1,4-dioxane/H_2_O (10:1), 100 °C, 10 h, 52%; (i) Fe, conc. HCl (aq), 70 °C, 2 h; (j) 2-(7-Azabenzotriazol-1-yl)-*N,’,N’,N’*-tetramethyluronium hexafluorophosphate (HATU), 17-oxo-21-((*3aS*,*4S*,*6aR*)-2-oxohexahydro-1*H*-thieno[3,4-d]imidazol-4-yl)-4,7,10,13-tetraoxa-16-azahenicosanoic acid, DCM, triethylamine (Et_3_N), rt, 20 min, 67%.

Synthesis of compound **14a** and the derivatives was illustrated in Scheme 1. Briefly, The commercially available 1-bromo-4-iodo-2-nitrobenzene underwent Buchwald-Hartwig reaction with *tert-*butyl ((*1R,4R*)-4-aminocyclohexyl)carbamate to produce intermediate **17**, which reacted with benzyl isocyanate **18** to afford urea **19**. Removal of Boc group allowed compound **19** to react with 6-fluoronicotinonitrile **20** to yield key intermediate **21**. Compound **21** reacted with 1-methyl-5-(4,4,5,5-tetramethyl-1,3,2-dioxaborolan-2-yl)pyridin-2(1*H*)-one under Suzuki coupling conditions to generate compound **22a**. Subsequent reduction of the nitro group of compound **22a** by Fe powder in acidic conditions, followed by coupling with acryloyl chloride yielded the first designed molecule **14a**. Alternatively, hydrogenation of **21** under catalysis of Pd/C yielded the debrominated aniline which reacted with acryloyl chloride to yield analogue **14b**. Compound **21** could also be converted to a series of analogs **22c-l** by a Buchwald-Hartwig reaction with a series of secondary amine derivatives, which were readily converted to analogues **14c-l** under similar procedure to that of **14a** and **14b**. A biotinylated probe **YJZ9149** was also synthesized by coupling compound **14m** and 17-oxo-21-((*3aS*,*4S*,*6aR*)-2-oxohexahydro-1*H*-thieno[3,4-*d*]imidazol-4-yl)-4,7,10,13-tetraoxa-16-azahenicosanoic acid.

### Discovery of YJZ5118 (14k) as a Potent and Highly Selective Irreversible CDK12/13 Inhibitor.

The kinase inhibitory activities of the compounds against CDK12 and CDK13 were preliminarily evaluated using the ADP-Glo kinase assay. Cell growth inhibition was determined in VCaP prostate cancer cells, which were characterized as being sensitive to CDK12/13 inhibition.^14^ Previously reported reversible CDK12/13 inhibitor **2** and the irreversible inhibitor **3** were included as the positive reference compounds and exhibited similar kinase inhibitory potencies to their reported data.^16, 17^ Compound **14a,** the initially designed molecule, showed strong CDK12/13 kinase inhibitory activities with IC_50_ values of 37.9 and 14.9 nM, respectively, comparable to the lead molecule **2** (Table 1). However, **14a** demonstrated approximately 6-fold higher antiproliferative potency in VCaP cells compared to the lead molecule **2**, with an IC_50_ value of 330.4 nM. These data suggested that **14a** may covalently target the kinases, extending target residence time and improving antiproliferative activity.

The methylpyridone group in **2** was originally introduced to improve aqueous solubility;^16^ however removal of this moiety in **14a** resulted in a 7- to 13-fold reduction in kinase inhibitory potency (**14b**), with IC_50_ values of 506 and 99 nM for CDK12 and CDK13, respectively. Interestingly, **14b** exhibited 3.4-fold more potency than **14a** in the growth inhibition assay against VCaP cells, likely due to its reduced hydrophilicity facilitating better cellular membrane penetration (Supporting Information Table S4).

It has been well documented that introducing an aliphatic amine adjacent to the acrylamide warhead could induce a localized basic environment to benefit the covalent bond formation by facilitating deprotonation of the sulfhydryl moiety of a cysteine.^31, 32^ Hydrophilicity of an aliphatic amine could also improve the PK properties of a lead compound by enhancing aqueous solubility. Thus, a hydrophilic *N*-methyl-*N*-(*N’,N*’-dimethyl)ethylamino group was first introduced and the resulting molecule **14c** partially restored CDK12/13 kinase inhibitory activities with IC_50_ values of 190.6 and 79.3 nM, respectively. Compound **14c** also exhibited significant antiproliferative activity, with an IC_50_ value of 44.4 nM, approximately 44-fold more potent than the lead compound **2**.

Replacement of the hydrophilic group in **14c** with other cyclic aliphatic amines (**14d-g**, **14j**) generally maintained kinase inhibitory potency and antiproliferative activity. However, compound **14i,** in which the aliphatic amine was neutralized by amide formation, exhibited 2- to 3-fold weaker potency against the kinases compared to its counterpart molecule **14f**. When a (*S*)-3,4-dimethylpiperazin-1-yl group was introduced, the resulting compound **14h** exhibited the strongest kinase inhibition and cell growth suppression in the series, with IC_50_ values of 33.1 nM, 21.7 nM, and 4.3 nM for CDK12, CDK13, and VCaP cells, respectively. KinomeScan profiling study further supported the target specificity of **14h** (Supporting Information Figure S1 and Table S1).

Despite its potency, compound **14h** exhibited unfavorable *in vivo* PK profile, with a short half-life (*T*_1/2_), poor oral exposure, and a high clearance rate (Supporting Information Table S3). To address these issues, compound **14k** (**YJZ5118**), incorporating a 4-(dimethylamino)piperidin-1-yl group, was developed. **YJZ5118** demonstrated similar CDK12/13 kinase inhibitory activities to **14h**, with IC_50_ values of 39.5 and 26.4 nM, respectively. The compound also potently suppressed VCaP cell growth with an IC_50_ value of 23.7 nM. Although the antiproliferative activity of **YJZ5118** was approximate 5.5-fold less potent than **14h**, this compound exhibited markedly improved PK properties in mice, with an AUC of 2235.5 h_*_ng/mL and *T*_1/2_ of 2.32 h upon 10 mg/kg oral administration (Figure 3A). The oral bioavailability of **YJZ5118** was 35.5%, and clearance rate of **YJZ5118** was 4-time lower than that of **14h** with a value of 28.34 mL/min/kg at 2 mg/kg i.v. dosing. During revising this manuscript, Insilico Medicine reported a new irreversible CDK12/13 inhibitor with oral bioavailability and *in vivo* efficacy^33^. However, **YJZ5118** exhibited a better PK profile.

Target specificity of **YJZ5118** was assessed against the CDK family, showing over 100-fold selectivity for CDK12/13 over other CDK family members, with the exception of CDK7 (Figure 3B). **YJZ5118** exhibited moderate inhibition against CDK7, with an IC_50_ value of 2263 nM, which was 57- and 86- fold higher than the values against CDK12 and CDK13, respectively. In contrast, the reversible lead molecule **2** exhibited significant inhibition of CDK7 and CDK9, with IC_50_ values of 678.6 and 514.8 nM, respectively. In conclusion, **YJZ5118** represented a new covalent CDK12/13 inhibitor with enhanced target specificity, cytotoxic effect and reasonable PK profiles, making it a promising candidate for further investigation.

### Validation of the Covalent Binding Mode of YJZ5118.

**Figure 4.**
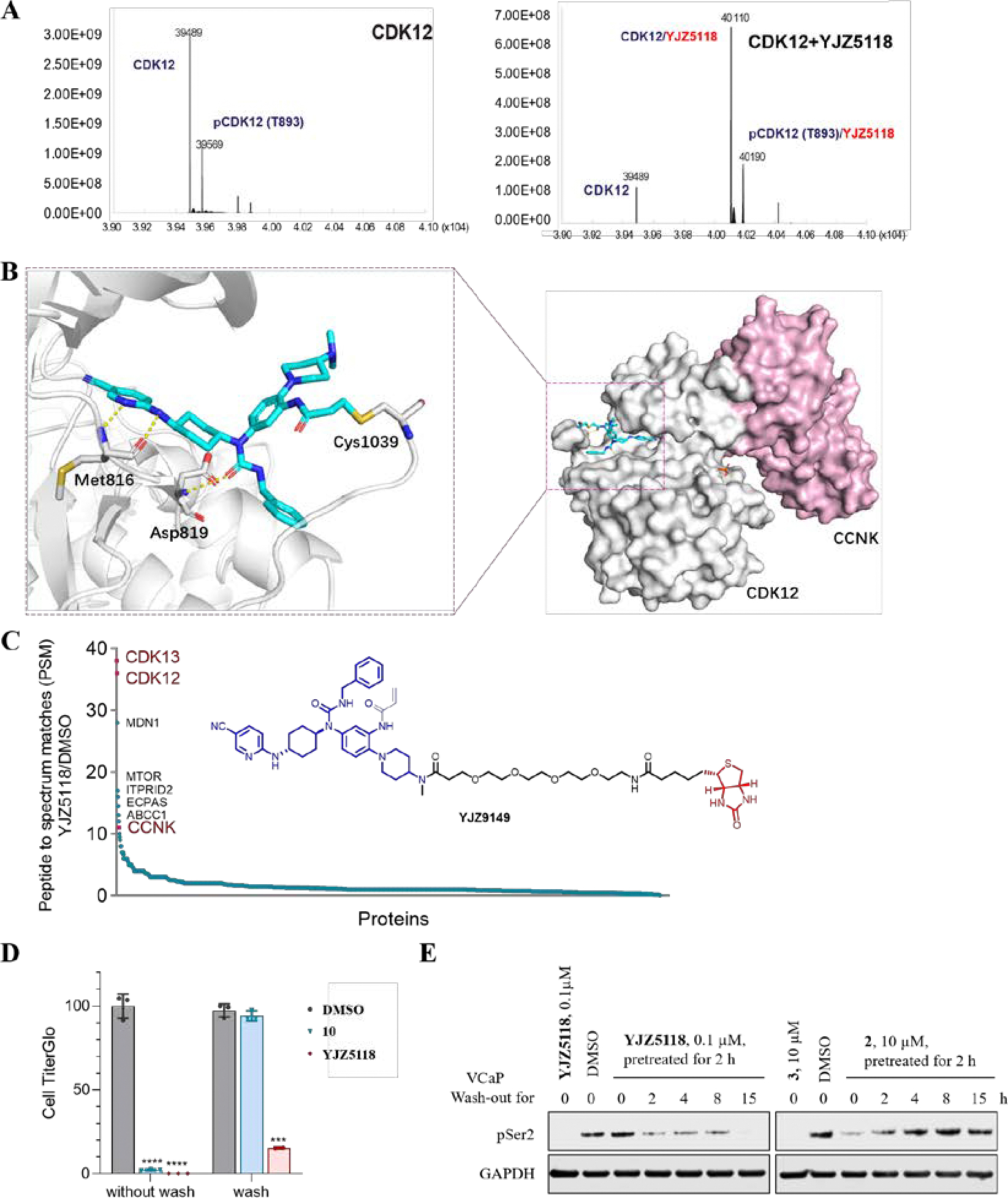
(A) Mass spectral peaks corresponding to CDK12 and CDK12 covalently labeled with compound **YJZ5118**; (B) Co-crystal structure of CDK12/CCNK and compound **YJZ5118** (PDB: 9JK1). Compound **YJZ5118** is shown in cyan stick structure. The key residues of CDK12 kinase are shown in gray sticks. Hydrogen bonds to key amino acids are indicated by yellow dashed lines; (C) Peptide to spectrum matches (PSM) proteomic analysis of pull-down experiments in whole cell lysate, and the structure of compound **YJZ9149** (biotinylated compound of **YJZ5118**); (D, E) Washout experiments using compound **YJZ5118** and reversible inhibitors **10** and **2** in VCaP cells.

To confirm that **YJZ5118** binds to its target protein in a covalent manner, we incubated the CDK12/CCNK complex with a 5-fold molar excess of the compound for 2 h at room temperature, followed by a liquid chromatograph-mass spectrometer (LC-MS) analysis. A mass shift consistent with the addition of a single molecule of **YJZ5118** to CDK12 was observed (Figure 4A). The covalent binding mode was further characterized by the co-crystal structure of **YJZ5118** with CDK12/CCNK complex at 2.5 Å resolution. The structure revealed that, in addition to two hydrogen bonds formed between the 6-aminonicotinonitrile moiety of **YJZ5118** with the hinge residue Met816 and one hydrogen bond formed between the urea group and Asp819, the acrylamide group formed a covalent bond with Cys1039 (Figure 4B). To further confirm covalent bond formation in cell lysates, we synthesized a biotinylated analog of **YJZ5118** (**YJZ9149**) to pull down covalently bound targets by streptavidin. As shown in Figure 4C, CDK12 and CDK13 were most enriched proteins in precipitate.

We next investigated the irreversible inhibition of **YJZ5118** on cell growth. The previously reported reversible inhibitor **10** was included for comparison. VCaP cells were treated with the compounds for 6 h, after which the compounds were removed, and the cells were cultured in compound-free media for 5 days. As shown in Figure 4D, while cell growth inhibitory effects of compound **10** was nearly completely lost following washout, **YJZ5118** maintained potent antiproliferative activity in VCaP cells even after washout (Figure 4D). Furthermore, **YJZ5118** inhibited the phosphorylation of RNA polymerase II at Ser2 for an extended period at a low concentration (100 nM), with complete loss of phosphorylation observed at 15 h after washout (Figure 4E). In contrast, the reversible counterpart **2** inhibited the RNA polymerase II Ser2 phosphorylation for a limited time at a higher concentration (10 μM), with the recovery of phosphorylation at 4 h after washout (Figure 4E). Furthermore, **YJZ5118** also maintained significant inhibitory effects on the expression of downstream gene RAD51 and markedly increased apoptotic marker c-PARP after pretreatment with **YJZ5118** for 2 h and then washout, but similar results were not observed upon the treatment of reversible inhibitor **2** (Supporting Information Figure S3A). These results collectively support that **YJZ5118** is an irreversible CDK12/13 kinase inhibitor.

### YJZ5118 Suppresses Transcription of DDR genes, and Provokes DNA Damage and Apoptosis in Multiple Cancer Cell Lines.

**Figure 5.**
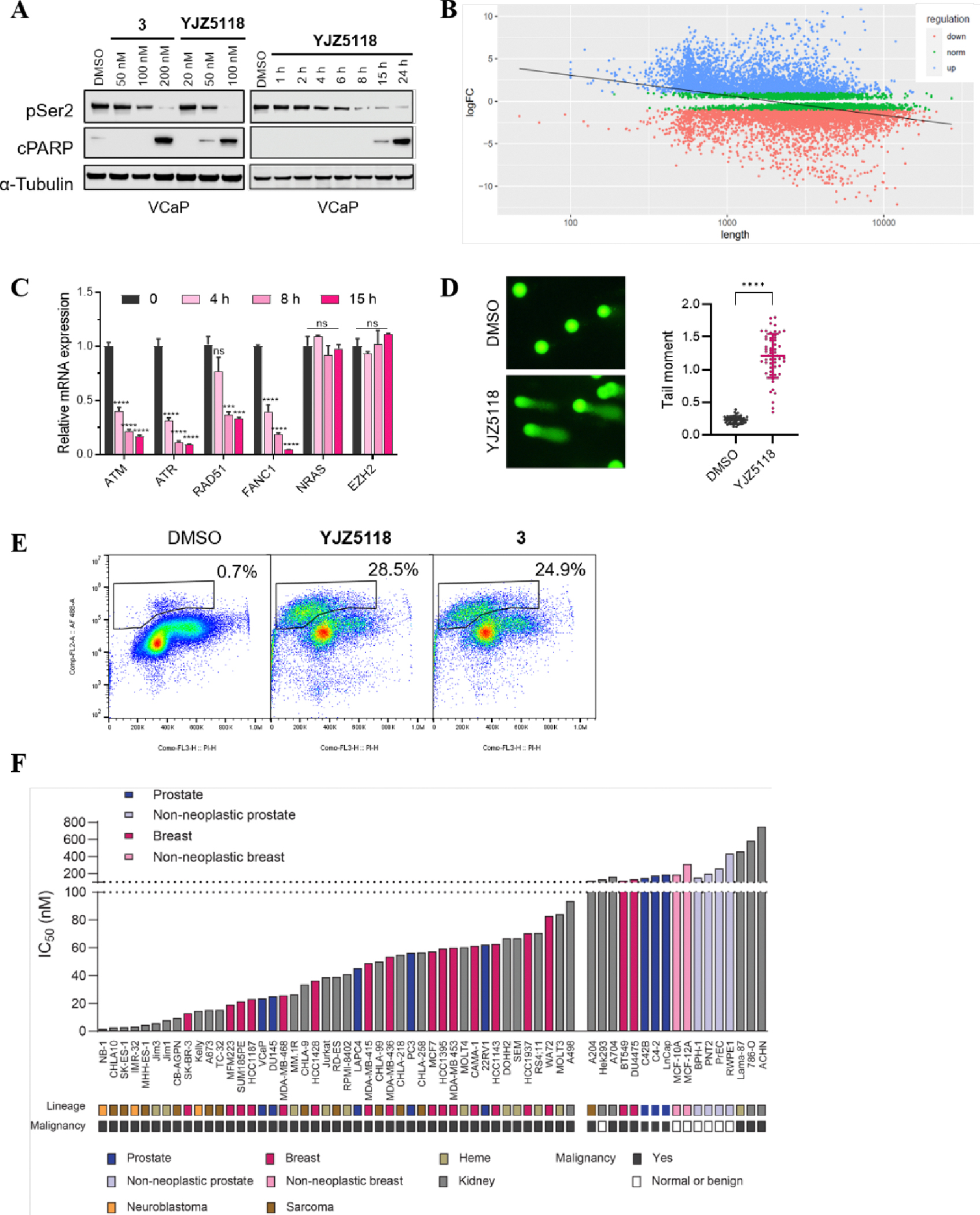
(A) Immunoblots of pSer2 of RNA polymerase II CTD and cleaved PARP in VCaP cells treated with compound **YJZ5118** or **3** at selected concentrations and time points. *α*-Tubulin is used as a loading control; (B) Scatter plot showing Log2 fold changes in gene expression vs. gene length in Log2 scale for each protein-coding gene in VCaP cells (p < 2.2e−16, F-test). Differentially expressed genes are indicated (FDR < 0.1 and Log2 FC > 1); (C) Analysis of indicated gene expression by qPCR at selected time points with 100 nM of compound **YJZ5118** in VCaP cells. Data are presented as mean values ± SD of triplicate points. *p ≤ 0.05, **p ≤ 0.01, ***p ≤ 0.001, ****p ≤ 0.0001 by t test; (D) Left: Representative images from comet assay of VCaP cells after 12 h of treatment with vehicle or compound **YJZ5118** (100 nM) stained with propidium iodide. Scale bar represents 50 μm; Right: Tail moments obtained from comet assay of VCaP cells after treatment with vehicle or compound **YJZ5118**. Horizontal bars denote the median. For each condition, 50 cells were analyzed. *p ≤ 0.05, **p ≤ 0.01, ***p ≤ 0.001, ****p ≤ 0.0001 by t test; (E) The apoptosis rate of VCaP cells was analyzed by flow cytometry using Annexin V/PI staining; (F) IC_50_ values of compound **YJZ5118** in a panel of human-derived cancer or normal cell lines after 5 days of treatment.

**YJZ5118** was demonstrated to inhibit phosphorylation of RNA polymerase II Ser2 in VCaP cells in a dose- and time-dependent manner (Figure 5A). Previous studies have reported that CDK12 inhibition leads to gene length-dependent elongation defects, causing premature cleavage and polyadenylation (PCPA) and the loss of expression of long genes, a substantial proportion of which are involved in the DDR.^13^ We performed RNA sequencing (RNA-seq) in VCaP cells treated with **YJZ5118** for 6 h. The results showed that a significant correlation between gene length and gene expression: longer genes were more likely to be downregulated (Figure 5B).

To assess if inhibition of CDK12/13 alters the expression of DDR genes, we performed quantitative PCR (qPCR) in cells treated with **YJZ5118**. We found treatment of VCaP cells with **YJZ5118** reduced the expression of a cast of DDR genes including *Ataxia Telangiectasia Mutated* (*ATM*), *Ataxia Telangiectasia and Rad3 Related* (*ATR*), *RAD51*, and *Fanconi Anemia Complementation Group I* (*FANC1*), as early as 4 h post-treatment (Figure 5C). Notably, the expression of other key cancer-related genes, such as *NRAS* and *Enhancer of Zeste Homolog 2* (*EZH2*), remained unaffected by **YJZ5118**.

To further investigate the DNA damage upon **YJZ5118** treatment, we performed neutral comet assays in VCaP cells and found a significant increase in neutral comet tails compared to DMSO (Figure 5D). Flow cytometry analysis showed that **YJZ5118** induced apoptosis in VCaP cell at the concentration of 100 nM, with an apoptosis rate of 28.5% (Figure 5E). Western blot analysis also confirmed that **YJZ5118** significantly increased cleaved PARP at a concentration of 100 nM (Figure 5A).

Next, we expanded the cell viability screening to a panel of normal and cancer cell lines from 8 different lineages. The results showed that several prostate cancer cells (*i.e.*, VCaP and DU145), breast cancer cells (*i.e.*, SK-BR-3, MFM223 and MDA-MD-468) and Ewing’s sarcoma cells (*i.e.*, CHLA10 and CB-AGPN), were preferentially sensitive to **YJZ5118**, with IC_50_ values of less than 40 nM. In contrast, normal and non-neoplastic cells exhibited much lower sensitive to the compound (Figure 5F).

### YJZ5118 Combined with Akt Inhibitors Shows a Synergistic Effect *In Vitro* and *In Vivo*.

**Figure 6.**
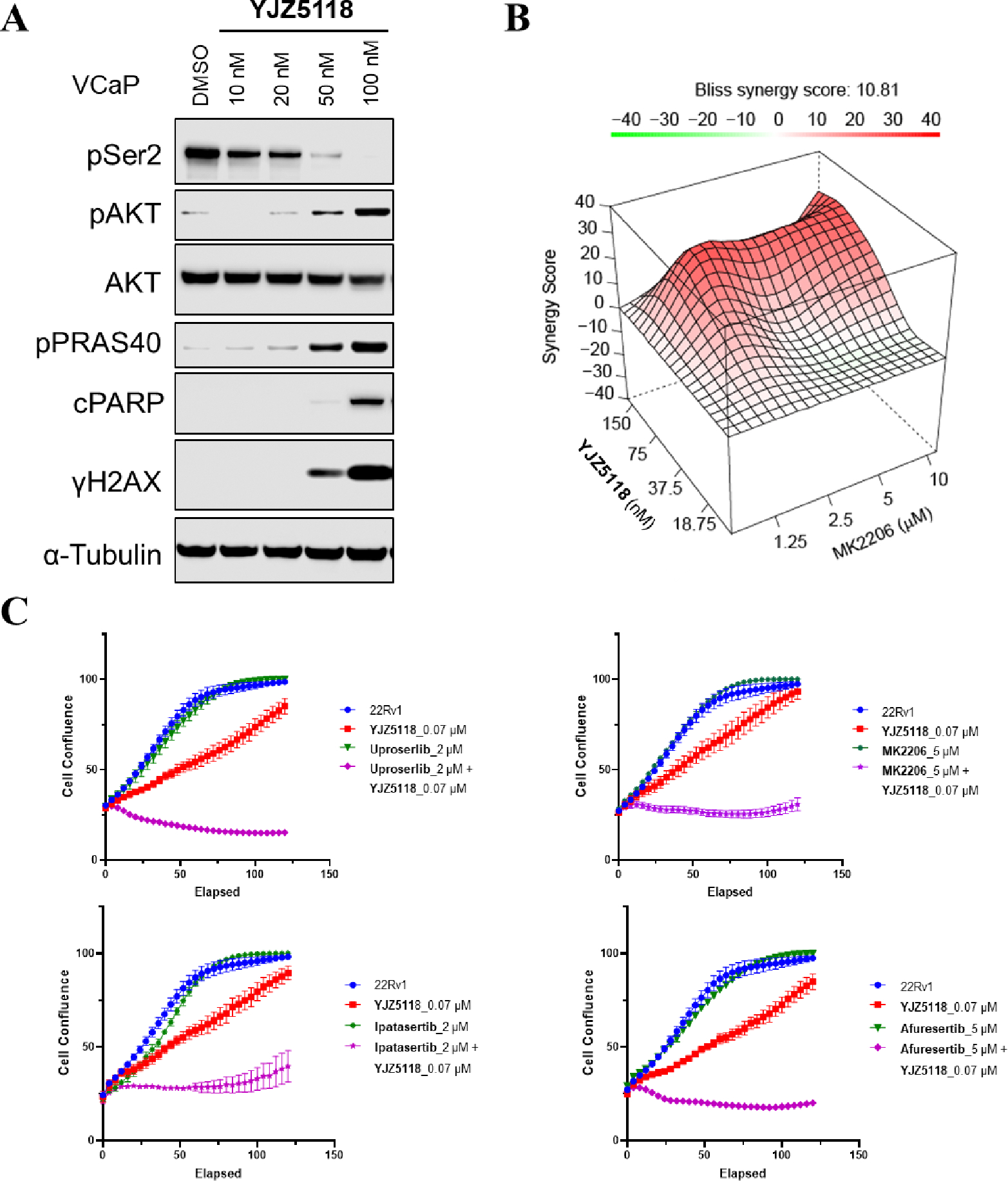
(A) Immunoblots of the indicated proteins at selected concentrations in VCaP cells after treated with compound **YJZ5118** for 24 h. α-Tubulin is used as a loading control; (B) 22RV1 cells were treated with compounds **YJZ5118** and/or **MK2206** at varied concentrations to determine the effect on cell growth and drug synergism, with assessments using the Loewe method. Red peaks in the 3D plots denote synergy with the average synergy scores noted above; (C) Real-time growth curves of 22RV1 cells upon treatment with compound **YJZ5118** and/or Akt inhibitors. Data are presented as mean +/− SD (n = 3) from one of three independent experiments.

Recently, our group reported that the CDK12/13 degrader **YJ1206** induced Akt phosphorylation and exhibited a synergistic effect with Akt inhibitors.^14^ To determine if blocking CDK12/13 kinase activity could also achieve a similar effect, we treated VCaP cells with **YJZ5118** for 24 h at different concentrations. As shown in Figure 6A, **YJZ5118** treatment increased phosphorylation of Akt and its direct substrate PRAS40 in a dose-dependent manner, while the total Akt levels remained unchanged. Additionally, **YJZ5118** treatment triggered DNA damage and apoptosis, as indicated by increased levels of γH2AX and cleaved-PARP (Figure 6A).

To investigate potential synergistic effects between **YJZ5118** and Akt inhibitors, we combined **YJZ5118** with **MK2206**, an Akt inhibitor, in 22RV1 cells, which exhibit moderate sensitivity to CDK12/13 inhibition (Figure 5F). The results revealed a significant synergistic effect between **YJZ5118** and **MK2206**, with a synergy score of 10.81 (Figure 6B). IncuCyte assays further demonstrated enhanced efficacy of the combinatorial treatment in 22RV1 cells (Figure 6C). Similar synergistic effects were also observed when **YJZ5118** was combined with other Akt inhibitors, including Uprosertib, Ipatasertib, and Afuresertib in 22RV1 cells (Figure 6C).

**Figure 7.**
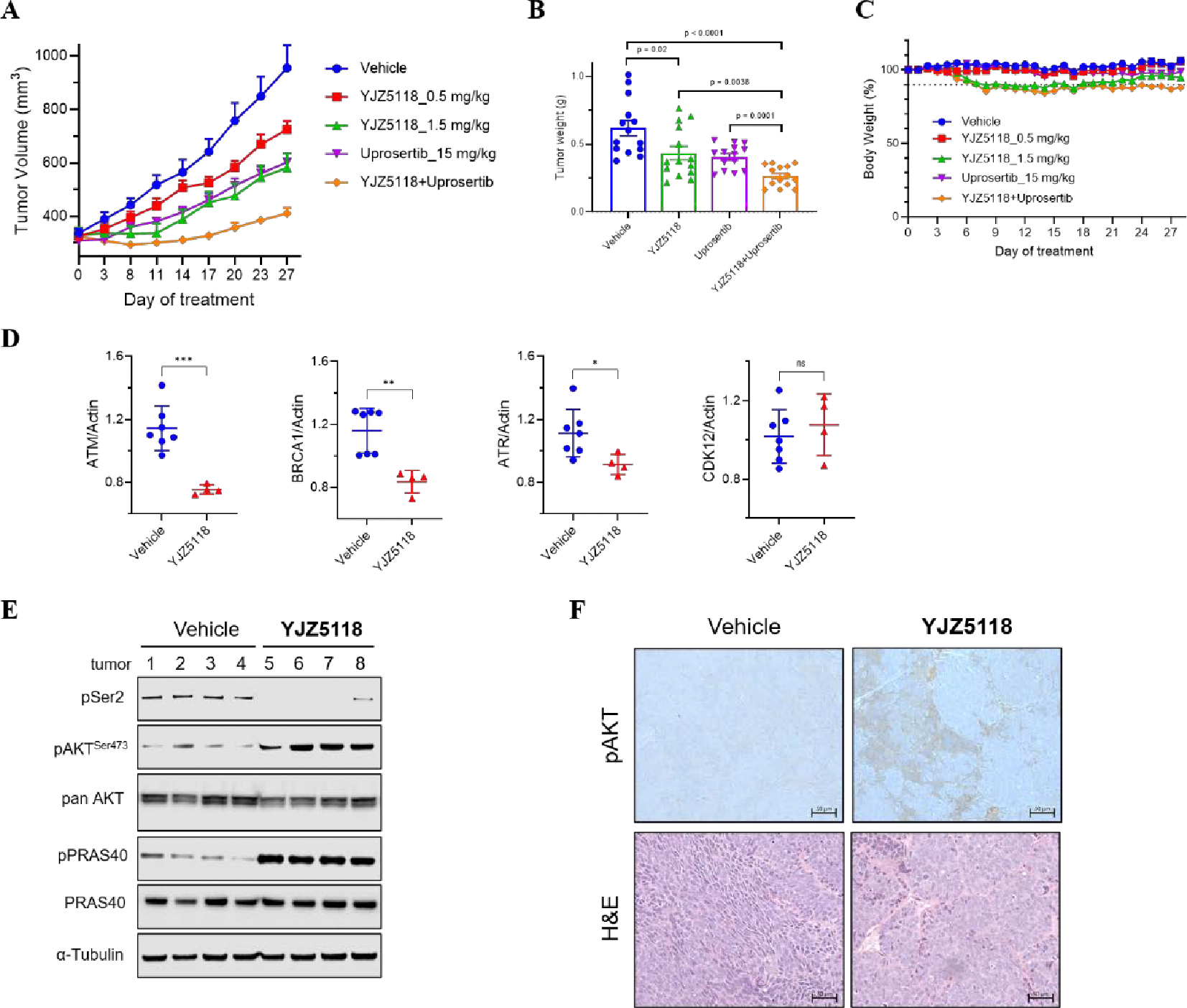
(A) Tumor volume (measured twice weekly using calipers) measurements of treated with compound **YJZ5118** (i.p., q.d.), uprosertib (p.o., 5x/week) or combination; (B) Waterfall plot depicting the change in tumor volume; (C) Percentage of mouse body weight throughout the treatment period. Data are presented as mean ± SEM; (D) Expression of indicated genes (Real-Time quantitative PCR assay) in tumors after the treatment of compound **YJZ5118** for 27 days in a VCaP CRPC model. Data are presented as mean values ± SD of triplicate points. *p ≤ 0.05, **p ≤ 0.01, ***p ≤ 0.001, by t test; (E) Immunoblots of the noted proteins from tumors after 27 days of treatment with compound **YJZ5118**. α-Tubulin is the loading control; (F) Representative H&E and pAKT staining for tumors after 27 days of treatment.

We further evaluated the *in vivo* anti-tumor activity of **YJZ5118** using a VCaP CRPC mouse model. Compared to the vehicle control, mice treated with **YJZ5118** or Uprosertib showed a significant reduction in tumor growth (Figures 7A, B). Encouragingly, the combination treatment of **YJZ5118** and Uprosertib markedly suppressed tumor growth, with no significant changes in animal body weights observed (Figure 7C). Endpoint analyses showed that **YJZ5118** effectively inhibited the phosphorylation of RNA polymerase II at Serine 2 and significantly increased pAkt (S473) and pPRAS40 levels in tumors compared to the vehicle group (Figure 7E). Additionally, **YJZ5118** treatment reduced the expression of DDR genes in tumors, whereas the expression of the CDK12 gene remained unaffected (Figure 7D).

Histological analysis of the tumors further confirmed that inhibition of CDK12/13 kinase activity led to increased pAkt levels (Figure 7F). Moreover, enhanced apoptosis was evident in the H&E-stained tumor sections (Figure 7F). These results demonstrate that **YJZ5118** not only provokes Akt phosphorylation but also exhibits a synergistic anti-tumor effect when combined with Akt inhibitors. While both CDK12/13 inhibitor **YJZ5118** and CDK12/13 degrader **YJ1206** exhibited a similar synergistic anti-tumor effect with Akt inhibition, they bear different mechanisms of action, *e.g.* prolonged target engagement of covalent CDK12/13 inhibitors and catalytic event-driven pharmacology of CDK12/13 degraders, which could result in distinct profiles with regard to potency, selectivity, PK properties and toxicity.

## CONCLUSIONS

In summary, starting from the reversible inhibitor **2**, **YJZ5118** was developed as a novel covalent inhibitor of CDK12/13 through structure-based optimization. **YJZ5118** showed significantly enhanced antiproliferative activity and improved target specificity. The covalent binding mode with CDK12/13 was comprehensively validated through mass spectrometry analysis, co-crystal structure studies, and pulldown-proteomic experiments. **YJZ5118** efficiently inhibited RNA polymerase II Ser2 phosphorylation, suppressed transcription of DNA damage response genes, and induced DNA damage and apoptosis. Furthermore, **YJZ5118** significantly inhibited the proliferation of multiple tumor cell lines, while normal and non-neoplastic cells were less sensitive. Notably, **YJZ5118** was shown to induce Akt phosphorylation via CDK12/13 inhibition. Synergistic anti-tumor effects were observed when **YJZ5118** was combined with Akt inhibitors, both *in vitro* and *in vivo*, highlighting its therapeutic potential.

## EXPERIMENTAL SECTION

### General Methods for Chemistry.

All commercially available reagents and solvents were used without further purification. All chemical reactions were monitored by thin-layer chromatography (TLC) plates with visualization under UV light (254 or 365 nm). ^1^H NMR spectra were performed with Bruker AV-400/600 spectrometer, ^13^C NMR spectra were recorded on Bruker AV-600 spectrometer at 150 MHz, internal reference was either TMS or deuterated NMR solvent. Low-resolution mass spectra (MS) were recorded on an Agilent 1200 HPLC-MSD mass spectrometer. High resolution mass spectral analysis was recorded on an Applied Biosystems Q-STAR Elite ESI-LC-MS/MS mass spectrometer. Purity of all final compounds was confirmed to be >95% by HPLC analysis with the Agilent 1260 system. The analytical columns were YMC-Triart C18 reversed-phase column, 5 *μ*m, 4.6 mm × 250 mm, and flow rate 1.0 mL/min.

#### tert-butyl ((1r,4r)-4-((4-bromo-3-nitrophenyl)amino)cyclohexyl)carbamate (14).

To a solution of 1-bromo-4-iodo-2-nitrobenzene **12** (25.0 g, 76.2 mmol) in toluene (400 mL) were added *tert*-butyl ((1r,4r)-4-aminocyclohexyl)carbamate **13** (16.3 g, 76.2 mmol), Cs_2_CO_3_ (29.8 g, 91.5 mmol), Pd_2_(dba)_3_ (1.74 g, 1.9 mmol) and Xantphos (2.2 g, 3.8 mmol). The mixture was evacuated and backfilled with argon (3 cycles). The reaction mixture was then heated at 100 °C for 12 h before being filtered through celite. The reaction solvent was evaporated under reduced pressure and purified by silica gel column chromatography to afford the title compound as yellow solid (26.0 g, yield 82%): ^1^H NMR (400 MHz, DMSO-*d*_6_) δ 7.43 (d, *J* = 8.8 Hz, 1H), 7.09 (d, *J* = 2.2 Hz, 1H), 6.80 (d, *J* = 7.7 Hz, 1H), 6.75 (d, *J* = 8.8 Hz, 1H), 6.33 (d, *J* = 7.9 Hz, 1H), 3.28–3.09 (m, 2H), 1.93 (d, *J* = 11.1 Hz, 2H), 1.78 (d, *J* = 12.3 Hz, 2H), 1.38 (s, 9H), 1.28 (q, *J* = 12.5, 12.1 Hz, 2H), 1.16 (q, *J* = 12.0 Hz, 2H). MS (ESI), *m/z*: 412.0 [M-H]^−^.

#### tert-butyl ((1r,4r)-4-(3-benzyl-1-(4-bromo-3-nitrophenyl)ureido)cyclohexyl)carbamate (16).

To a solution of *tert*-butyl ((1r,4r)-4-((4-bromo-3-nitrophenyl)amino)cyclohexyl)carbamate **14** (25 g, 60.3 mmol) and DIPEA (1.59 g, 12.3 mmol) in DMF (10 mL) was added benzyl isocyanate **15** (24 g, 181.0 mmol) at room temperature. The mixture was stirred at 95 °C for 4 h. The solvent was removed under reduced pressure and purified by column chromatography to give yellow solid (26 g, yield 79%): ^1^H NMR (400 MHz, DMSO-*d*_6_) δ 7.94 (d, *J* = 8.5 Hz, 1H), 7.84 (s, 1H), 7.40 (d, *J* = 8.5, 1H), 7.32–7.22 (m, 3H), 7.21–7.14 (m, 2H), 6.68 (d, *J* = 8.0 Hz, 1H), 6.43 (t, *J* = 6.3 Hz, 1H), 4.21–4.09 (m, 3H), 3.00 (s, 1H), 1.81–1.68 (d, *J* = 11.3 Hz, 4H), 1.35 (s, 9H), 1.28–1.19 (m, 2H), 1.13–1.04 (m, 2H). MS (ESI), *m/z*: 569.1 [M+Na]^+^.

#### 3-benzyl-1-(4-bromo-3-nitrophenyl)-1-((1r,4r)-4-((5-cyanopyridin-2-yl)amino)cyclohexyl)urea (18).

TFA (10 mL) was added to a solution of *tert*-butyl ((1r,4r)-4-(3-benzyl-1-(4-bromo-3-nitrophenyl)ureido)cyclohexyl)carbamate **16** (22.8 g, 41.6 mmol) in DCM (20 mL) and the mixture was refluxed at 50 °C for 3 h. After that the reaction mixture was concentrated to dryness under reduced pressure, it was then extracted with EtOAc (3x) and washed with H_2_O. The combined EtOAc layers were subsequently dried over MgSO_4_, and the solvent was removed under reduced pressure. The obtained crude material was used for next step without further purification. To a solution of the above deprotected crude product (14.4 g, 32.2 mmol) in DMF (30 mL) were added 5-cyano-2-fluoropyridine **17** (3.9 g, 32.2 mmol), and Cs_2_CO_3_ (12.6 g, 38.6 mmol). The mixture was stirred at room temperature for 10 minutes. Then the reaction mixture was heated at 60 °C for another 60 minutes. The reaction mixture was then filtered and the solvent was removed under reduced pressure. The crude material was purified by column chromatography to afford **18** as yellow solid (15.4 g, yield 80%): ^1^H NMR (400 MHz, DMSO-*d*_6_) δ 8.30 (d, *J* = 2.4 Hz, 1H), 7.95 (d, *J* = 8.4 Hz, 1H), 7.87 (d, *J* = 2.4 Hz, 1H), 7.61 (d, *J* = 8.9 Hz, 1H), 7.48 (d, *J* = 7.6 Hz, 1H), 7.42 (dd, *J* = 8.4, 2.5 Hz, 1H), 7.33–7.24 (m, 2H), 7.23–7.15 (m, 3H), 6.52–6.40 (m, 2H), 4.27 (t, *J* = 12.3 Hz, 1H), 4.17 (d, *J* = 5.9 Hz, 2H), 3.56 (s, 1H), 1.93 (d, *J* = 12.2 Hz, 2H), 1.83 (d, *J* = 12.0 Hz, 2H), 1.32 (q, *J* = 12.4 Hz, 2H), 1.24–1.09 (m, 2H). MS (ESI), *m/z*: 549.1 [M+H]^+^.

#### 3-benzyl-1-((1r,4r)-4-((5-cyanopyridin-2-yl)amino)cyclohexyl)-1-(4-(1-methyl-6-oxo-1,6-dihydropyridin-3-yl)-3-nitrophenyl)urea (19a).

A mixture of compound **18** (300 mg, 0.5 mmol), K_2_CO_3_ (69 mg, 1.0 mmol), Pd(PPh_3_)_4_ (57.8 mg, 0.05 mmol), and 1-methyl-5-(4,4,5,5-tetramethyl-1,3,2-dioxaborolan-2-yl)pyridin-2(1*H*)-one (141 mg, 0.6 mmol) in 1,4-dioxane/H_2_O (10:1, 60 mL) was evacuated and backfilled with argon. After stirring at 100 °C for 10 h, the solvent was removed under vacuum, and the resultant crude residue redissolved in EtOAc (20 mL), which was washed with H_2_O (60 mL). The layers was separated and the aqueous phase was extracted with EtOAc (2 × 20 mL). The combined EtOAc layers were subsequently dried over MgSO_4_, and the solvent was removed under reduced pressure. The crude material was purified by silica column chromatography to afford the title compound as white solid (150 mg, yield 52%). ^1^H NMR (400 MHz, DMSO-*d*_6_) δ 8.30 (d, *J* = 2.3 Hz, 1H), 7.95 (d, *J* = 2.7 Hz, 1H), 7.82 (d, *J* = 2.0 Hz, 1H), 7.65 – 7.56 (m, 3H), 7.51 (d, *J* = 7.6 Hz, 1H), 7.39 – 7.35 (m, 1H), 7.32 – 7.26(m, 2H), 7.24 – 7.17 (m, 3H), 6.47 (t, *J* = 8.8 Hz, 2H), 6.41 (t, *J* = 6.0 Hz, 1H), 4.29 (t, *J* = 11.9 Hz, 1H), 4.20 (d, *J* = 5.9 Hz, 2H), 3.58 – 3.51 (m, 1H) 3.50 (s, 3H), 1.91 (dd, *J* = 23.5, 12.3 Hz, 4H), 1.35 (q, *J* = 11.4 Hz, 2H), 1.18 (q, *J* = 11.4 Hz, 2H). MS (ESI), *m/z*: 575.6 [M-H]^−^.

#### 3-benzyl-1-((1r,4r)-4-((5-cyanopyridin-2-yl)amino)cyclohexyl)-1-(4-((2-(dimethylamino)ethyl)(methyl)amino)-3-nitrophenyl)urea (19c).

A mixture of 3-benzyl-1-(4-bromo-3-nitrophenyl)-1-((1r,4r)-4-((5-cyanopyridin-2-yl)amino)cyclohexyl)urea **18** (200 mg, 0.36 mmol), *N*^*1*^*,N*^*1*^*,N*^*2*^*-trimethylethane-1,2-diamine* (44.1 mg, 0.43 mmol), Cs_2_CO_3_ (237.4 mg·0.72 mmol), Pd_2_(dba)_3_ (36.6 mg·0.04 mmol) and Xantphos (41.6 mg, 0.07 mmol) in 20 mL toluene : DMF (10 : 1) was evacuated and backfilled with argon (3 cycles) and then heated at 100 °C for 12 h. The mixture was filtered through Celite after cooling, concentrated in vacuo, and purified by column chromatography to afford the title compound as yellow solid (110 mg, yield 50%): ^1^H NMR (400 MHz, DMSO-*d*_6_) δ 8.30 (s, 1H), 7.61 (d, *J* = 8.9 Hz, 1H), 7.54–7.44 (d, *J* = 11.3 Hz, 2H), 7.32–7.23 (m, 4H), 7.21–7.13 (d, *J* = 7.4 Hz, 3H), 6.48 (d, *J* = 8.9 Hz, 1H), 6.19 (t, *J* = 6.1 Hz, 1H), 4.25 (t, *J* = 12.6 Hz, 1H), 4.16 (d, *J* = 5.9 Hz, 2H), 3.51 (s, 1H), 3.28 (t, *J* = 7.0 Hz, 3H), 3.18 (s, 1H), 2.81 (s, 3H), 2.47 (t, *J* = 6.8 Hz, 2H), 2.15 (s, 6H), 1.92 (d, *J* = 11.3 Hz, 2H), 1.80 (d, *J* = 12.4 Hz, 2H), 1.31 (q, *J* = 13.2, 12.5 Hz, 2H), 1.12 (q, *J* = 12.9 Hz, 2H). MS (ESI), *m/z*: 569.2 [M-H]^−^.

#### 3-benzyl-1-((1r,4r)-4-((5-cyanopyridin-2-yl)amino)cyclohexyl)-1-(4-(3-(dimethylamino)azetidin-1-yl)-3-nitrophenyl)urea (19d).

Compound **19d** was synthesized from commercially available *N,N*-dimethylazetidin-3-amine and **18** by following a similar procedure as that of **19c** (yield, 40%). ^1^H NMR (400 MHz, DMSO-*d*_6_) δ 8.30 (s, 1H), 7.61 (d, *J* = 9.0 Hz, 1H), 7.56 (s, 1H), 7.48 (d, *J* = 7.5 Hz, 1H), 7.27 (t, *J* = 7.8 Hz, 3H), 7.21–7.13 (m, 3H), 6.82 (d, *J* = 8.9 Hz, 1H), 6.47 (d, *J* = 8.9 Hz, 1H), 6.18 (t, *J* = 5.8 Hz, 1H), 4.26 (t, *J* = 11.9 Hz, 1H), 4.15 (d, *J* = 5.9 Hz, 2H), 4.00 (t, *J* = 8.2 Hz, 2H), 3.78–3.66 (m, 2H), 3.51 (s, 1H), 3.12 (p, *J* = 6.3 Hz, 1H), 2.11 (s, 6H), 1.92 (d, *J* = 12.3 Hz, 2H), 1.80 (d, *J* = 12.0 Hz, 2H), 1.31 (q, *J* =13.9 Hz, 2H), 1.11 (q, *J* = 12.6 Hz, 2H). MS (ESI), *m/z*: 569.3 [M+H]^+^.

#### 3-benzyl-1-((1r,4r)-4-((5-cyanopyridin-2-yl)amino)cyclohexyl)-1-(4-(3-(dimethylamino)pyrrolidin-1-yl)-3-nitrophenyl)urea (19e).

Compound **19e** was synthesized from commercially available *N,N*-dimethylpyrrolidin-3-amine and **18** by following a similar procedure as that of **19c** (yield, 41%). ^1^H NMR (400 MHz, DMSO-*d*_6_) δ 8.29 (s, 1H), 7.61 (d, *J* = 9.0 Hz, 1H), 7.51 (d, *J* = 2.4 Hz, 1H), 7.48 (d, *J* = 7.4 Hz, 1H), 7.31–7.22 (m, 3H), 7.21–7.14 (m, 3H), 7.11 (d, *J* = 8.9 Hz, 1H), 6.47 (d, *J* = 8.9 Hz, 1H), 6.15 (t, *J* = 6.0 Hz, 1H), 4.31–4.22 (m, 1H), 4.21–4.08 (m, 2H), 3.51 (s, 1H), 3.43–3.38 (m, 1H), 3.17 (q, *J* = 8.4, 7.4 Hz, 3H), 2.75 (t, *J* = 7.8 Hz, 1H), 2.25–2.11 (s, 7H), 1.92 (d, *J* = 11.9 Hz, 2H), 1.85–1.71 (m, 3H), 1.30 (q, *J* = 12.4 Hz, 2H), 1.18–1.02 (m, 2H). MS (ESI), *m/z*: 583.3 [M-H]^+^.

#### 3-benzyl-1-((1r,4r)-4-((5-cyanopyridin-2-yl)amino)cyclohexyl)-1-(4-(4-methylpiperazin-1-yl)-3-nitrophenyl)urea (19f).

Compound **19f** was synthesized from commercially available 1-methylpiperazine and **18** by following a similar procedure as that of **19c** (yield, 58%). ^1^H NMR (400 MHz, DMSO-*d*_6_) δ 8.30 (s, 1H), 7.65–7.55 (m, 2H), 7.48 (d, *J* = 8.4 Hz, 1H), 7.39–7.34 (m, 1H), 7.29 (dd, *J* = 15.5, 8.0 Hz, 3H), 7.18 (d, *J* = 7.3 Hz, 3H), 6.48 (d, *J* = 8.9 Hz, 1H), 6.24 (t, *J* = 6.2 Hz, 1H), 4.28 (t, *J* = 10.9 Hz, 1H), 4.17 (d, *J* = 5.9 Hz, 2H), 3.52 (s, 1H), 3.05 (t, *J* = 4.5 Hz, 4H), 2.44 (t, *J* = 4.5 Hz, 4H), 2.23 (s, 3H), 1.93 (d, *J* = 11.2 Hz, 2H), 1.81 (d, *J* = 12.1 Hz, 2H), 1.32 (q, *J* = 12.3, 11.9 Hz, 2H), 1.12 (q, *J* = 10.1, 8.0 Hz, 2H). MS (ESI), *m/z*: 567.2 [M-H]^−^.

#### 3-benzyl-1-((1r,4R)-4-((5-cyanopyridin-2-yl)amino)cyclohexyl)-1-(4-((1R,4R)-5-methyl-2,5-diazabicyclo[2.2.1]heptan-2-yl)-3-nitrophenyl)urea (19g).

Compound **19g** was synthesized from commercially available (1*R*,4*R*)-2-methyl-2,5-diazabicyclo[2.2.1]heptane and **18** by following a similar procedure as that of **19c** (yield, 40%). MS (ESI), *m/z*: 581.3 [M+H]^+^.

#### 3-benzyl-1-((1r,4S)-4-((5-cyanopyridin-2-yl)amino)cyclohexyl)-1-(4-((S)-3,4-dimethylpiperazin-1-yl)-3-nitrophenyl)urea (19h).

Compound **19h** was synthesized from commercially available (*S*)-1,2-dimethylpiperazine and **18** by following a similar procedure as that of **19h** (yield, 50%). ^1^H NMR (400 MHz, DMSO-*d*_6_) δ 8.30 (s, 1H), 7.64–7.57 (m, 2H), 7.48 (d, *J* = 7.6 Hz, 1H), 7.38–7.25 (m, 4H), 7.22–7.14 (m, 3H), 6.48 (d, *J* = 8.8 Hz, 1H), 6.23 (t, *J* = 5.8 Hz, 1H), 4.32–4.22 (m, 1H), 4.16 (d, *J* = 5.9 Hz, 2H), 3.52 (s, 1H), 3.15–3.04 (m, 2H), 2.99 (t, *J* = 11.1 Hz, 1H), 2.78 (d, *J* = 12.2 Hz, 1H), 2.65 (t, *J* = 10.7 Hz, 1H), 2.31–2.13 (m, 5H), 1.92 (d, *J* = 12.1 Hz, 2H), 1.81 (d, *J* = 12.4 Hz, 2H), 1.32 (q, *J* = 12.1 Hz, 2H), 1.11 (q, *J* = 12.5 Hz, 2H), 1.01 (d, *J* = 6.3 Hz, 3H). MS (ESI), *m/z*: 583.3 [M+H]^+^.

#### 1-(4-(4-acetylpiperazin-1-yl)-3-nitrophenyl)-3-benzyl-1-((1r,4r)-4-((5-cyanopyridin-2-yl)amino)cyclohexyl)urea (19i).

Compound **19i** was synthesized from commercially available 1-(piperazin-1-yl)ethan-1-one and **18** by following a similar procedure as that of **19c** (yield, 64%). ^1^H NMR (400 MHz, DMSO-*d*_6_) δ 8.30 (s, 1H), 7.63 (s, 1H), 7.61 (d, *J* = 8.7 Hz, 1H), 7.50 (d, *J* = 7.7 Hz, 1H), 7.43–7.37 (m, 1H), 7.37–7.32 (m, 1H), 7.28 (t, *J* = 7.6 Hz, 2H), 7.22–7.15 (m, 3H), 6.48 (d, *J* = 8.9 Hz, 1H), 6.26 (t, *J* = 6.0 Hz, 1H), 4.26 (t, *J* = 12.0 Hz, 1H), 4.16 (d, *J* = 5.9 Hz, 2H), 3.57 (s, 4H), 3.45 (s, 1H), 3.07 (d, *J* = 20.0 Hz, 4H), 2.04 (s, 3H), 1.92 (d, *J* = 12.2 Hz, 2H), 1.82 (d, *J* = 12.1 Hz, 2H), 1.32 (q, *J* = 12.3 Hz, 2H), 1.12 (q, *J* = 11.0, 9.9 Hz, 2H). MS (ESI), *m/z*: 596.9 [M+H]^+^.

#### 3-benzyl-1-((1r,4r)-4-((5-cyanopyridin-2-yl)amino)cyclohexyl)-1-(4-morpholino-3-nitrophenyl)urea (19j).

Compound **19j** was synthesized from commercially available morpholine and **18** by following a similar procedure as that of **19c** (yield, 51%). ^1^H NMR (400 MHz, DMSO-*d*_6_) δ 8.30 (s, 1H), 7.67–7.55 (m, 2H), 7.50 (d, *J* = 7.6 Hz, 1H), 7.43–7.37 (m, 1H), 7.36–7.31 (m, 1H), 7.28 (t, *J* = 7.5 Hz, 2H), 7.21–7.15 (d, *J* = 7.5 Hz, 3H), 6.48 (d, *J* = 8.9 Hz, 1H), 6.26 (t, *J* = 6.0 Hz, 1H), 4.27 (t, *J* = 12.2 Hz, 1H), 4.16 (d, *J* = 5.9 Hz, 2H), 3.71 (t, *J* = 4.1 Hz, 4H), 3.52 (s, 1H), 3.05 (t, *J* = 4.5 Hz, 4H), 1.92 (d, *J* = 12.2 Hz, 2H), 1.82 (d, *J* = 12.2 Hz, 2H), 1.32 (q, *J* = 12.4 Hz, 2H), 1.12 (q, *J* = 12.6 Hz, 2H). MS (ESI), *m/z*: 556.1 [M+H]^+^.

#### 3-benzyl-1-((1r,4r)-4-((5-cyanopyridin-2-yl)amino)cyclohexyl)-1-(4-(4-(dimethylamino)piperidin-1-yl)-3-nitrophenyl)urea (19k).

Compound **19k** was synthesized from commercially available *N,N*-dimethylpiperidin-4-amine and **18** by following a similar procedure as that of **19c** (yield, 40%).^1^H NMR (400 MHz, DMSO-*d*_6_) δ 8.30 (d, *J* = 2.3 Hz, 1H), 7.61 (dd, *J* = 8.9, 2.3 Hz, 1H), 7.58 (d, *J* = 2.4 Hz, 1H), 7.49 (d, *J* = 7.8 Hz, 1H), 7.35 (dd, *J* = 8.8, 2.5 Hz, 1H), 7.32–7.25 (m, 3H), 7.18 (td, *J* = 5.5, 3.0 Hz, 3H), 6.48 (d, *J* = 9.0 Hz, 1H), 6.24 (t, *J* = 6.1 Hz, 1H), 4.33–4.21 (m, 1H), 4.16 (d, *J* = 6.1 Hz, 2H), 3.51 (s, 1H), 3.28 (d, *J* = 12.4 Hz, 2H), 2.86 (td, *J* = 12.2, 2.3 Hz, 2H), 2.24 (s, 1H), 2.21 (s, 6H), 1.92 (d, *J* = 12.0 Hz, 2H), 1.82 (t, *J* = 12.7 Hz, 4H), 1.49 (q, *J* = 11.9 Hz, 2H), 1.31 (q, *J* = 14.8, 13.7 Hz, 2H), 1.18–1.04 (m, 2H). MS (ESI), *m/z*: 597.2 [M+H]^+^.

#### tert-butyl (1-(4-(3-benzyl-1-((1r,4r)-4-((5-cyanopyridin-2-yl)amino)cyclohexyl)ureido)-2-nitrophenyl)piperidin-4-yl)(methyl)carbamate (19l).

Compound **19l** was synthesized from commercially available *tert*-butyl methyl(piperidin-4-yl)carbamate and **18** by following a similar procedure as that of **19c** (yield, 62%). ^1^H NMR (400 MHz, DMSO-*d*_6_) δ 8.30 (d, *J* = 2.3 Hz, 1H), 7.65 – 7.56 (m, 2H), 7.48 (d, *J* = 7.7 Hz, 1H), 7.39 – 7.31 (m, 2H), 7.31 – 7.24 (m, 2H), 7.21 – 7.15 (m, 3H), 6.47 (d, *J* = 8.9 Hz, 1H), 6.24 (t, *J* = 5.9 Hz, 1H), 4.26 (t, *J* = 12.0 Hz, 1H), 4.16 (d, *J* = 5.9 Hz, 2H), 4.07 – 3.75 (m, 1H), 3.59 – 3.45 (m, 1H), 3.30 (t, *J* = 12.0 Hz, 2H), 2.94 (t, *J* = 12.0 Hz, 2H), 2.72 (s, 3H), 1.92 (d, *J* = 11.8 Hz, 2H), 1.85 – 1.72 (m, 4H), 1.64 (d, *J* = 9.5 Hz, 2H), 1.42 (s, 9H), 1.32 (q, *J* = 12.9, 12.0 Hz, 2H), 1.11 (q, *J* = 13.5, 12.8 Hz, 2H). MS (ESI), *m/z*: 683.4 [M+H]^+^.

#### N-(5-(3-benzyl-1-((1r,4r)-4-((5-cyanopyridin-2-yl)amino)cyclohexyl)ureido)-2-(1-methyl-6-oxo-1,6-dihydropyridin-3-yl)phenyl)acrylamide (14a).

To a solution of **19a** (810 mg, 1.4 mmol) in H_2_O/EtOH (3:7, 30 mL), 0.05 mL of concentrated HCl and Fe powder (602 mg, 7 mmol) were added. The mixture was stirred for 2 h at 70 °C. The mixture was filtered through Celite after cooling, concentrated in vacuo to dryness to afford the crude product as brown solid, which was used in the next step without further purification. To the solution of the crude product and DIPEA (361.2 mg, 2.8 mmol) in anhydrous DCM (30 mL), which was cooled to 0°C, was added acryloyl chloride (216.7 mg, 1.7 mmol) slowly. The reaction mixture was stirred for 10 min at 0 °C to rt for 15 min. The mixture was quenched with H_2_O, the pH was adjusted to 7–8 with saturated NaHCO_3_ solution, and extracted with EtOAc three times. The combined organic phase was concentrated in vacuo and purified by column chromatography to give the desired compound **14a** (352 mg, yield 42%, two steps). ^1^H NMR (600 MHz, DMSO-*d*_6_) δ 9.55 (s, 1H), 8.32 (d, *J* = 2.3 Hz, 1H), 7.85 (d, *J* = 2.6 Hz, 1H), 7.67–7.59 (m, 2H), 7.56 (s, 1H), 7.44 (dd, *J* = 9.4, 2.6 Hz, 1H), 7.39 (d, *J* = 8.1 Hz, 1H), 7.28 (t, *J* = 7.6 Hz, 2H), 7.19 (dd, *J* = 17.0, 7.7 Hz, 3H), 7.07 (d, *J* = 8.1 Hz, 1H), 6.53–6.42 (m, 3H), 6.24 (d, *J* = 19.0 Hz, 1H), 5.95 (t, *J* = 6.2 Hz, 1H), 5.74 (d, *J* = 10.0 Hz, 1H), 4.30 (tt, *J* = 12.0, 3.7 Hz, 1H), 4.19 (d, *J* = 5.9 Hz, 2H), 3.51(s, 1H), 3.48 (s, 4H), 1.94 (d, *J* = 10.6 Hz, 2H), 1.82 (d, *J* = 10.9 Hz, 2H), 1.34 (q, *J* = 11.2 Hz, 2H), 1.20 (q, *J* = 12.3, 11.0 Hz, 2H). ^13^C NMR (151 MHz, DMSO-*d*_6_) δ 164.11, 161.68, 159.51, 156.85, 153.36, 141.63, 141.16, 139.83, 137.77, 136.02, 132.00, 130.90, 130.56, 128.52 (4C), 127.99, 127.55, 127.21 (4C), 126.74, 119.51, 119.24, 116.10, 94.53, 53.79, 49.06, 43.98, 40.51, 37.41, 31.75, 30.66. HRMS (ESI) calcd for C_35_H_35_N_7_O_3_ [M + H]^+^, 602.2874; found, 602.2853. HPLC analysis: MeOH-H_2_O (70:30), 5.91 min, 98.7% purity.

#### N-(3-(3-benzyl-1-((1r,4r)-4-((5-cyanopyridin-2-yl)amino)cyclohexyl)ureido)phenyl)acrylamide (14b).

To a solution of the compound **18** (250 mg, 0.42 mmol) in methanol (20 mL) was added 10% Pd/C (25 mg, 10% w/w) at room temperature. The reaction mixture was stirred under hydrogen balloon for 3 h and then filtered through celite. The filtrate was evaporated to dryness to give the crude material, which was used in the next step without further purification. To the solution of the crude product and DIPEA (81.3 mg, 0.63 mmol) in anhydrous DCM (30 mL), which was cooled to 0°C, was added acryloyl chloride (45.3 mg, 0.5 mmol) slowly. The reaction mixture was stirred for 10 min at 0 °C to rt for 15 min. The mixture was quenched with H_2_O, the pH was adjusted to 7–8 with saturated NaHCO_3_ solution, and extracted with EtOAc three times. The combined organic phase was concentrated in vacuo and purified by column chromatography to give the desired compound **14b** (82 mg, yield 39%, two steps): ^1^H NMR (400 MHz, DMSO-*d*_6_) δ 10.28 (s, 1H), 8.30 (d, *J* = 2.3 Hz, 1H), 7.73 (d, *J* = 8.2 Hz, 1H), 7.61 (dd, *J* = 8.8, 2.4 Hz, 1H), 7.56 (d, *J* = 2.1 Hz, 1H), 7.48 (d, *J* = 7.6 Hz, 1H), 7.41 (t, *J* = 8.0 Hz, 1H), 7.30–7.23 (m, 2H), 7.22–7.13 (m, 3H), 6.89 (d, *J* = 8.5 Hz, 1H), 6.50–6.46 (m, 1H), 6.46–6.39 (m, 1H), 6.28 (dd, *J* = 16.9, 2.1 Hz, 1H), 5.85 (t, *J* = 6.0 Hz, 1H), 5.79 (dd, *J* = 10.0, 2.1 Hz, 1H), 4.34–4.23 (m, 1H), 4.16 (d, *J* = 6.0 Hz, 2H), 3.50 (s, 1H), 1.93 (d, *J* = 11.9 Hz, 2H), 1.79 (d, *J* = 11.6 Hz, 2H), 1.32 (q, *J* = 12.5 Hz, 2H), 1.17 (q, *J* = 12.5 Hz, 2H). ^13^C NMR (151 MHz, DMSO-*d*_6_) δ 163.71, 156.84, 153.58, 141.74, 140.40, 138.81, 132.19, 130.07, 128.48 (4C), 127.70, 127.15 (4C), 126.70, 126.59, 122.18, 119.60, 119.09, 94.45, 53.60, 49.07, 43.94, 40.49, 31.76, 30.66. HRMS (ESI) calcd for C_29_H_30_N_6_O_2_ [M + H]^+^, 495.2503; found, 495.2485. HPLC analysis: MeOH-H_2_O (70:30), 11.84 min, 95.9% purity.

#### N-(5-(3-benzyl-1-((1r,4r)-4-((5-cyanopyridin-2-yl)amino)cyclohexyl)ureido)-2-((2-(dimethylamino)ethyl)(methyl)amino)phenyl)acrylamide (14c).

Compound **14c** was synthesized from compound **19c** with a similar procedure to that of **14a** (yield 38%, two steps). ^1^H NMR (400 MHz, DMSO-*d*_6_) δ 10.22 (s, 1H), 8.31 (d, *J* = 2.3 Hz, 1H), 8.24 (d, *J* = 2.5 Hz, 1H), 7.61 (dd, *J* = 8.9, 2.4 Hz, 1H), 7.50 (d, *J* = 7.6 Hz, 1H), 7.33 (d, *J* = 8.4 Hz, 1H), 7.30–7.24 (m, 2H), 7.21–7.14 (m, 3H), 6.89 (dd, *J* = 8.4, 2.5 Hz, 1H), 6.48 (d, *J* = 8.7 Hz, 1H), 6.45–6.39 (m, 1H), 6.30 (dd, *J* = 17.0, 2.2 Hz, 1H), 5.82 (dd, *J* = 9.9, 2.2 Hz, 1H), 5.76 (t, *J* = 6.0 Hz, 1H), 4.33–4.23 (m, 1H), 4.18 (d, *J* = 6.0 Hz, 2H), 3.52 (s, 1H), 2.83 (t, *J* = 5.7 Hz, 2H), 2.72 (s, 3H), 2.40 (t, *J* = 5.6 Hz, 2H), 2.23 (s, 6H), 1.93 (d, *J* = 12.1 Hz, 2H), 1.79 (d, *J* = 11.9 Hz, 2H), 1.32 (q, *J* = 12.3 Hz, 2H), 1.18 (dd, *J* = 28.1, 15.7 Hz, 2H). ^13^C NMR (151 MHz, DMSO-*d*_6_) δ 163.64, 159.70, 157.03, 153.58, 142.77, 141.73, 134.93, 134.13, 132.27, 128.49 (4C), 127.67, 127.06 (4C), 126.69, 122.88, 122.48, 119.60, 94.45, 57.38, 56.72, 53.71, 49.0, 46.18 (2C), 43.91, 41.48, 40.52, 31.79, 30.65. HRMS (ESI) calcd for C_34_H_42_N_8_O_2_ [M + H]^+^, 595.3503; found, 595.3508. HPLC analysis: MeOH-H_2_O (80:20), 8.92 min, 95.3% purity.

#### N-(5-(3-benzyl-1-((1r,4r)-4-((5-cyanopyridin-2-yl)amino)cyclohexyl)ureido)-2-(3-(dimethylamino)azetidin-1-yl)phenyl)acrylamide (14d).

Compound **14d** was synthesized from compound **19d** with a similar procedure to that of **14a** (yield 35%, two steps). ^1^H NMR (400 MHz, DMSO-*d*_6_) δ 9.38 (s, 1H), 8.33 (d, *J* = 2.3 Hz, 1H), 7.61 (dd, *J* = 8.9, 2.4 Hz, 1H), 7.49 (d, *J* = 7.6 Hz, 1H), 7.30–7.23 (m, 2H), 7.20–7.14 (m, 3H), 7.11 (d, *J* = 2.4 Hz, 1H), 6.90 (dd, *J* = 8.6, 2.4 Hz, 1H), 6.61 (d, *J* = 8.5 Hz, 1H), 6.55 (dd, *J* = 17.0, 10.2 Hz, 1H), 6.47 (d, *J* = 8.9 Hz, 1H), 6.24 (dd, *J* = 17.0, 2.1 Hz, 1H), 5.74 (dd, *J* = 10.2, 2.1 Hz, 1H), 5.59 (t, *J* = 6.1 Hz, 1H), 4.31–4.20 (m, 1H), 4.17 (d, *J* = 6.0 Hz, 2H), 3.98 (t, *J* = 7.2 Hz, 2H), 3.60 (t, *J* = 6.7 Hz, 2H), 3.50 (s, 1H), 3.12 (d, *J* = 14.5 Hz, H), 2.11 (s, 6H), 1.96–1.85 (m, 2H), 1.76 (d, *J* = 12.0 Hz,2H), 1.30 ((q, *J* = 11.9 Hz, 2H)), 1.14 (q, *J* = 11.7 Hz, 2H). ^13^C NMR (151 MHz, DMSO-*d*_6_) δ 163.95, 159.70, 157.21, 153.63, 145.74, 141.66, 132.15, 129.79, 129.24, 128.52 (4C), 128.29, 127.16 (4C), 126.72, 124.77, 119.62, 114.23, 94.44, 57.44, 56.28, 53.50, 49.06, 46.06, 43.96, 41.93 (2C), 40.51, 31.77, 30.61. HRMS (ESI) calcd for C_34_H_40_N_8_O_2_ [M + H]^+^, 593.3347; found, 593.3329. HPLC analysis: MeOH-H_2_O (70:30), 12.20 min, 96.1% purity.

#### N-(5-(3-benzyl-1-((1r,4r)-4-((5-cyanopyridin-2-yl)amino)cyclohexyl)ureido)-2-(3-(dimethylamino)pyrrolidin-1-yl)phenyl)acrylamide (14e).

Compound **14e** was synthesized from compound **19e** with a similar procedure to that of **14a** (yield 35%, two steps). ^1^H NMR (600 MHz, DMSO-*d*_6_) δ 9.49 (s, 1H), 8.32 (d, *J* = 2.3 Hz, 1H), 7.61 (dd, *J* = 9.0, 2.4 Hz, 1H), 7.51 (s, 1H), 7.31–7.23 (m, 2H), 7.21–7.13 (m, 4H), 6.89 (s, 2H), 6.57 (dd, *J* = 17.0, 10.2 Hz, 1H), 6.48 (d, *J* = 8.9 Hz, 1H), 6.24 (dd, *J* = 17.0, 2.0 Hz, 1H), 5.74 (dd, *J* = 10.1, 2.0 Hz, 1H), 5.58 (s, 1H), 4.26 (td, *J* = 10.1, 8.3, 6.0 Hz, 1H), 4.17 (d, *J* = 6.2 Hz, 2H), 3.51 (s, 1H), 3.27–3.15 (m, 3H), 2.66 (p, *J* = 7.8 Hz, 1H), 2.16 (s, 6H), 2.12–2.05 (m, 1H), 1.92 (s, 2H), 1.80–1.74 (m, 2H), 1.74–1.68 (m, 1H), 1.32 (q, *J* = 13.0, 12.2 Hz, 2H), 1.24 (d, *J* = 5.2 Hz, 1H), 1.21–1.11 (m, 2H). ^13^C NMR (151 MHz, DMSO-*d*_6_) δ 164.02, 159.70, 157.19, 153.61, 144.28, 141.64, 132.26, 130.32, 129.22, 128.52 (4C), 127.18 (4C), 126.96, 126.73, 126.08, 119.61, 116.16, 94.44, 65.62, 55.29, 53.49, 49.61, 49.07, 44.42 (2C), 43.97, 40.52, 31.78, 30.63, 30.10. HRMS (ESI) calcd for C_35_H_42_N_8_O_2_ [M + H]^+^, 607.3503; found, 607.3480. HPLC analysis: MeOH-H_2_O (70:30), 16.19 min, 99.1% purity.

#### N-(5-(3-benzyl-1-((1r,4r)-4-((5-cyanopyridin-2-yl)amino)cyclohexyl)ureido)-2-(4-methylpiperazin-1-yl)phenyl)acrylamide (14f).

Compound **14f** was synthesized from compound **19f** with a similar procedure to that of **14a** (yield 46%, two steps). ^1^H NMR (400 MHz, DMSO-*d*_6_) δ 9.08 (s, 1H), 8.31 (d, *J* = 2.3 Hz, 1H), 8.05 (s, 1H), 7.61 (dd, *J* = 8.9, 2.4 Hz, 1H), 7.52 (d, *J* = 7.6 Hz, 1H), 7.31–7.24 (m, 3H), 7.18 (dt, *J* = 6.4, 1.6 Hz, 3H), 6.95 (dd, *J* = 8.4, 2.4 Hz, 1H), 6.75 (dd, *J* = 16.9, 10.2 Hz, 1H), 6.47 (d, *J* = 8.9 Hz, 1H), 6.32 (dd, *J* = 17.0, 1.9 Hz, 1H), 5.89–5.79 (m, 2H), 4.34–4.22 (m, 1H), 4.18 (d, *J* = 6.0 Hz, 2H), 3.69–3.37 (m, 5H), 3.18 (s, 2H) 3.07 (d, *J* = 19.4 Hz, 2H), 2.88 (s, 3H), 1.92 (d, *J* = 11.8 Hz, 2H), 1.80 (d, *J* = 11.5 Hz, 2H), 1.39–1.27 (m, 2H), 1.21–1.09 (m, 2H). ^13^C NMR (151 MHz, DMSO-*d*_6_) δ 163.77, 159.68, 156.95, 153.57, 141.68, 134.73, 133.46, 132.53, 128.50 (4C), 127.80, 127.49, 127.11(4C), 126.72, 124.38, 121.42, 119.60, 94.46, 53.66, 53.30 (2C), 49.01, 48.78 (2C), 43.92, 42.93, 40.52, 31.74, 30.66. HRMS (ESI) calcd for C_34_H_40_N_8_O_2_ [M + H]^+^, 593.3347; found, 593.3365. HPLC analysis: MeOH-H_2_O (80:30), 6.87 min, 100% purity.

#### N-(5-(3-benzyl-1-((1r,4R)-4-((5-cyanopyridin-2-yl)amino)cyclohexyl)ureido)-2-((1R,4R)-5-methyl-2,5-diazabicyclo[2.2.1]heptan-2-yl)phenyl)acrylamide (14g).

Compound **14g** was synthesized from compound **19g** with a similar procedure to that of **14a** (yield 30%, two steps). ^1^H NMR (400 MHz, DMSO-*d*_6_) δ 9.30 (s, 1H), 8.33 (d, *J* = 2.4 Hz, 1H), 7.61 (dd, *J* = 8.8, 2.4 Hz, 1H), 7.49 (d, *J* = 7.6 Hz, 1H), 7.30–7.23 (m, 2H), 7.17 (dt, *J* = 9.0, 3.0 Hz, 3H), 7.13 (d, *J* = 2.1 Hz, 1H), 6.90–6.79 (m, 2H), 6.54 (dd, *J* = 17.0, 10.2 Hz, 1H), 6.47 (d, *J* = 8.9 Hz, 1H), 6.23 (dd, *J* = 17.1, 2.1 Hz, 1H), 5.73 (dd, *J* = 10.1, 2.1 Hz, 1H), 5.59 (d, *J* = 6.2 Hz, 1H), 4.31–4.20 (m, 1H), 4.17 (d, *J* = 6.1 Hz, 2H), 4.09 (s, 1H), 3.51 (s, 1H), 3.39 (dd, *J* = 8.9, 2.4 Hz, 2H), 3.07 (d, *J* = 9.0 Hz, 1H), 2.81 (d, *J* = 9.6 Hz, 1H), 2.65 (dd, *J* = 9.7, 2.3 Hz, 1H), 2.26 (s, 3H), 1.92 (d, *J* = 11.2 Hz, 2H), 1.85–1.71 (m, 3H), 1.68 (d, *J* = 9.2 Hz, 1H), 1.31 (dt, *J* = 15.5, 11.6 Hz, 3H), 1.14 (t, *J* = 13.5 Hz, 3H). ^13^C NMR (151 MHz, DMSO-*d*_6_) δ 163.80, 159.70, 157.21, 153.62, 143.65, 141.64, 132.28, 130.50, 128.98, 128.52 (4C), 128.12, 127.15 (4C), 126.94, 126.72, 119.61, 116.85, 94.44, 63.28, 60.35, 58.66, 53.53, 49.07, 43.96, 41.82, 40.52, 34.36, 31.78, 29.56. HRMS (ESI) calcd for C_35_H_40_N_8_O_2_ [M + H]^+^, 605.3347; found, 605.3329. HPLC analysis: MeOH-H_2_O (70:30), 10.96 min, 100% purity.

#### N-(5-(3-benzyl-1-((1r,4S)-4-((5-cyanopyridin-2-yl)amino)cyclohexyl)ureido)-2-((S)-3,4-dimethylpiperazin-1-yl)phenyl)acrylamide (14h).

Compound **14h** was synthesized from compound **19h** with a similar procedure to that of **14a** (yield 40%, two steps). ^1^H NMR (400 MHz, DMSO-*d*_6_) δ 9.02 (s, 1H), 8.31 (d, *J* = 2.3 Hz, 1H), 7.91 (s, 1H), 7.60 (dd, *J* = 8.9, 2.3 Hz, 1H), 7.49 (d, *J* = 7.6 Hz, 1H), 7.30–7.24 (m, 2H), 7.21 (d, *J* = 8.4 Hz, 1H), 7.20–7.15 (m, 3H), 6.92 (dd, *J* = 8.4, 2.4 Hz, 1H), 6.65 (dd, *J* = 16.9, 10.2 Hz, 1H), 6.47 (d, *J* = 8.9 Hz, 1H), 6.27 (dd, *J* = 17.0, 1.9 Hz, 1H), 5.78 (td, *J* = 9.6, 3.9 Hz, 2H), 4.33–4.22 (m, 1H), 4.17 (d, *J* = 6.0 Hz, 2H), 3.51 (s, 1H), 2.95–2.75 (m,4H), 2.45 (d, *J* = 10.6 Hz, 2H), 2.37 (d, *J* = 9.4 Hz, 1H), 2.25 (s, 3H), 1.92 (d, *J* = 12.0 Hz, 2H), 1.78 (d, *J* = 12.0 Hz, 2H), 1.32 (q, *J* = 12.5 Hz, 2H), 1.16 (t, *J* = 12.8 Hz, 2H), 1.01 (d, *J* = 6.1 Hz, 3H). ^13^C NMR (151 MHz, DMSO-*d*_6_) δ 170.83, 163.61, 159.67, 156.99, 153.59, 142.86, 141.73, 133.64, 132.91, 132.56, 128.49 (4C), 127.52, 127.10 (4C), 126.69, 124.58, 120.84, 119.62, 94.43, 58.86, 57.59, 53.61, 51.79, 49.05, 43.91, 42.64 (2C), 40.49, 31.75, 30.64, 17.22. HRMS (ESI) calcd for C_35_H_42_N_8_O_2_ [M + H]^+^, 607.3503; found, 607.3521. HPLC analysis: MeOH-H_2_O (80:20), 7.19 min, 95.1% purity.

#### N-(2-(4-acetylpiperazin-1-yl)-5-(3-benzyl-1-((1r,4r)-4-((5-cyanopyridin-2-yl)amino)cyclohexyl)ureido)phenyl)acrylamide (14i).

Compound **14i** was synthesized from compound **19i** with a similar procedure to that of **14a** (yield 40%, two steps). ^1^H NMR (400 MHz, DMSO-*d*_6_) δ 9.15 (s, 1H), 8.31 (dd, *J* = 2.4, 0.7 Hz, 1H), 8.02–7.95 (m, 1H), 7.61 (dd, *J* = 8.8, 2.4 Hz, 1H), 7.50 (d, *J* = 7.6 Hz, 1H), 7.30–7.24 (m, 2H), 7.22 (d, *J* = 8.4 Hz, 1H), 7.20–7.15 (m, 3H), 6.92 (dd, *J* = 8.4, 2.5 Hz, 1H), 6.75 (dd, *J* = 16.9, 10.2 Hz, 1H), 6.48 (d, *J* = 8.9 Hz, 1H), 6.30 (dd, *J* = 17.0, 1.9 Hz, 1H), 5.85–5.78 (m, 2H), 4.26 (d, *J* = 11.9 Hz, 1H), 4.17 (d, *J* = 6.0 Hz, 2H), 3.72–3.63 (m, 4H), 3.51 (s, 1H), 2.86 (t, *J* = 5.0 Hz, 2H), 2.81 (t, *J* = 5.1 Hz, 2H), 2.06 (s, 3H),1.93 (d, *J* = 9.7 Hz, 2H), 1.79 (d, *J* = 11.4 Hz, 2H), 1.32 (q, *J* = 12.4, 12.0 Hz, 2H), 1.18 (q, J = 12.4, 12.0 Hz, 2H). ^13^C NMR (151 MHz, DMSO-*d*_6_) δ 168.83, 163.71, 159.67, 156.99, 153.59, 141.73, 134.09, 133.26, 132.57, 128.49 (4C), 127.66, 127.49, 127.10 (4C), 126.69, 124.44, 121.28, 119.62, 94.43, 60.24, 53.66, 52.10, 51.61, 49.07, 46.27, 43.91, 41.37, 40.49, 31.75, 30.63, 21.78. HRMS (ESI) calcd for C_35_H_40_N_8_O_3_ [M + H]^+^, 621.3296; found, 621.3295. HPLC analysis: MeOH-H_2_O (70:30), 8.39 min, 100% purity.

#### N-(5-(3-benzyl-1-((1r,4r)-4-((5-cyanopyridin-2-yl)amino)cyclohexyl)ureido)-2-morpholinophenyl)acrylamide (14j).

Compound **14j** was synthesized from compound **19j** with a similar procedure to that of **14a** (yield 42%, two steps). ^1^H NMR (400 MHz, DMSO-*d*_6_) δ 9.13 (s, 1H), 8.31 (dd, *J* = 2.4, 0.7 Hz, 1H), 7.95 (s, 1H), 7.61 (dd, *J* = 8.9, 2.4 Hz, 1H), 7.50 (d, *J* = 7.6 Hz, 1H), 7.30–7.22 (m, 3H), 7.22–7.15 (m, 3H), 6.94 (dd, *J* = 8.4, 2.5 Hz, 1H), 6.73 (dd, *J* = 16.9, 10.2 Hz, 1H), 6.48 (d, *J* = 8.9 Hz, 1H), 6.28 (dd, *J* = 16.9, 1.9 Hz, 1H), 5.80 (dt, *J* = 10.2, 3.3 Hz, 2H), 4.35–4.22 (m, 1H), 4.17 (d, *J* = 6.0 Hz, 2H), 3.83 (t, *J* = 4.5 Hz, 4H), 3.50 (s, H), 2.86 (t, *J* = 4.6 Hz, 4H), 1.93 (d, *J* = 11.9 Hz, 2H), 1.79 (d, *J* = 11.6 Hz, 2H), 1.32 (q, *J* = 12.2 Hz, 2H), 1.18 (q, *J* = 12.2 Hz, 2H). ^13^C NMR (151 MHz, DMSO-*d*_6_) δ 163.69, 159.69, 157.00, 153.59, 142.78, 141.72, 133.92, 133.10, 132.58, 128.49 (4C), 127.59, 127.11 (4C), 126.69, 124.60, 120.99, 119.60, 94.45, 66.64 (2C), 53.69, 52.16 (2C), 49.00, 43.93, 40.52, 31.76, 30.65. HRMS (ESI) calcd for C_33_H_37_N_7_O_3_ [M + H]^+^, 580.3031; found, 5890.3024. HPLC analysis: MeOH-H_2_O (70:30), 12.33 min, 99.1% purity.

#### N-(5-(3-benzyl-1-((1r,4r)-4-((5-cyanopyridin-2-yl)amino)cyclohexyl)ureido)-2-(4-(dimethylamino)piperidin-1-yl)phenyl)acrylamide (14k, YJZ5118).

Compound **YJZ5118** was synthesized from compound **19k** with a similar procedure to that of **14a** (yield 40%, two steps). ^1^H NMR (400 MHz, DMSO-*d*_6_) δ 9.01 (s, 1H), 8.31 (d, *J* = 2.4 Hz, 1H), 7.92 (s, 1H), 7.60 (dd, *J* = 9.0, 2.4 Hz, 1H), 7.49 (d, *J* = 7.5 Hz, 1H), 7.30–7.23 (m, 2H), 7.22–7.13 (m, 4H), 6.90 (dd, *J* = 8.3, 2.5 Hz, 1H), 6.73 (dd, *J* = 16.9, 10.2 Hz, 1H), 6.47 (d, *J* = 8.9 Hz, 1H), 6.28 (dd, *J* = 17.0, 2.0 Hz, 1H), 5.84–5.72 (m, 2H), 4.32–4.22 (m, 1H), 4.17 (d, *J* = 6.0 Hz, 2H), 3.50 (s, 1H), 3.07 (d, *J* = 11.4 Hz, 2H), 2.65 (t, *J* = 11.2 Hz, 2H), 2.23 (s, 6H), 2.17 (d, *J* = 11.1 Hz, 1H),1.92 (d, *J* = 11.9 Hz, 2H), 1.85 (d, *J* = 11.4 Hz, 2H), 1.78 (d, *J* = 12.9 Hz, 2H), 1.74–1.68 (m, 2H), 1.32 (q, *J* = 12.2 Hz, 2H), 1.22–1.09 (m, 2H). ^13^C NMR (151 MHz, DMSO-*d*_6_) δ 163.68, 159.69, 157.06, 153.57, 143.52, 141.69, 133.43, 132.91, 132.60, 128.50 (3C), 127.54, 127.44, 127.10 (4C), 126.71, 124.43, 120.75, 119.60, 94.45, 61.87, 53.69, 51.64 (2C), 49.02, 43.92, 42.07 (2C), 40.47, 31.76, 30.64, 28.79 (2C). HRMS (ESI) calcd for C_36_H_44_N_8_O_2_ [M + H]^+^, 621.3660; found, 621.3666. HPLC analysis: MeOH-H_2_O (80:20), 10.27 min, 100% purity.

#### tert-butyl (1-(2-acrylamido-4-(3-benzyl-1-((1r,4r)-4-((5-cyanopyridin-2-yl)amino)cyclohexyl)ureido)phenyl)piperidin-4-yl)(methyl)carbamate (14l).

Compound **14l** was synthesized from compound **19l** with a similar procedure to that of **14a** (yield 44%, two steps). ^1^H NMR (400 MHz, DMSO-*d*_6_) δ 9.04 (s, 1H), 8.30 (d, *J* = 2.3 Hz, 1H), 7.94 (s, 1H), 7.60 (dd, *J* = 8.9, 2.3 Hz, 1H), 7.48 (d, *J* = 7.6 Hz, 1H), 7.30–7.21 (m, 3H), 7.21–7.13 (m, 3H), 6.90 (dd, *J* = 8.3, 2.4 Hz, 1H), 6.74 (dd, *J* = 16.9, 10.2 Hz, 1H), 6.47 (d, *J* = 8.9 Hz, 1H), 6.29 (dd, *J* = 17.0, 1.8 Hz, 1H), 5.86–5.74 (m, 2H), 4.28 (t, *J* = 10.9 Hz, 1H), 4.17 (d, *J* = 6.0 Hz, 2H), 3.92 (s, 1H), 3.06 (d, *J* = 11.2 Hz, 2H), 2.77 (s, 3H), 2.75–2.68 (m, 2H), 2.06–1.95 (m, 2H), 1.91 (d, *J* = 12.4 Hz, 2H), 1.78 (d, *J* = 11.9 Hz, 2H), 1.61 (s, 2H), 1.41 (s, 9H), 1.31 (q, *J* = 12.4, 10.1 Hz, 3H), 1.15 (q, *J* = 12.5 Hz, 2H). MS (ESI), *m/z*: 707.4 [M+H]^+^.

#### N-(5-(3-benzyl-1-((1r,4r)-4-((5-cyanopyridin-2-yl)amino)cyclohexyl)ureido)-2-(4-(methylamino)piperidin-1-yl)phenyl)acrylamide (14m).

TFA (2 mL) was added to a solution of *tert*-butyl (1-(2-acrylamido-4-(3-benzyl-1-((1r,4r)-4-((5-cyanopyridin-2-yl)amino)cyclohexyl)ureido)phenyl)piperidin-4-yl)(methyl)carbamate **14l** (185 mg, 0.26 mmol) in DCM (6 mL), and the mixture was stirred at 50 °C for 3 h. The reaction mixture was then concentrated to dryness under reduced pressure. The resultant crude material was dissolved in EtOAc (20 mL) and H_2_O (40 mL) was added. Subsequently, the pH was adjusted to 7–8 with saturated NaHCO_3_ solution, and extracted with EtOAc three times. The combined organic phase was concentrated in vacuo and purified by column chromatography to give the desired compound **14m** (95 mg, yield 60%). ^1^H NMR (400 MHz, DMSO-*d*_6_) δ 9.10 (s, 1H), 8.66 (s, 2H), 8.30 (d, *J* = 2.3 Hz, 1H), 7.90 (s, 1H), 7.60 (dd, *J* = 8.9, 2.4 Hz, 1H), 7.50 (d, *J* = 7.6 Hz, 1H), 7.30–7.25 (m, 2H), 7.23 (d, *J* = 8.4 Hz, 1H), 7.21–7.14 (m, 3H), 6.93 (dd, *J* = 8.4, 2.4 Hz, 1H), 6.63 (dd, *J* = 17.0, 10.2 Hz, 1H), 6.47 (d, *J* = 8.9 Hz, 1H), 6.28 (dd, *J* = 17.0, 1.8 Hz, 1H), 5.84–5.74 (m, 2H), 4.34–4.23 (m, 1H), 4.18 (d, *J* = 6.0 Hz, 2H), 3.50 (s, 1H), 3.13 (dd, *J* = 14.3, 10.4 Hz, 3H), 2.79–2.68 (m, 2H), 2.62 (s, 3H), 2.08 (d, *J* = 11.9 Hz, 2H), 1.92 (d, *J* = 11.3 Hz, 2H), 1.87–1.73 (m, 4H), 1.32 (q, *J* = 12.5 Hz, 2H), 1.14 (q, *J* = 13.3, 12.0 Hz, 2H). ^13^C NMR (151 MHz, DMSO-*d*_6_) δ 163.62, 159.69, 156.99, 153.57, 142.88, 141.67, 134.01, 133.04, 132.53, 128.51 (4C), 127.50, 127.12 (4C), 126.72, 124.69, 121.25, 119.60, 94.46, 55.42, 53.68, 50.28, 49.03, 43.94, 40.52, 31.75, 30.67, 30.02 (2C), 28.56 (2C). HRMS (ESI) for C_35_H_42_N_8_O_2_ [M+H]^+^, calcd: 607.3503, found: 607.3489. HPLC analysis: MeOH-H_2_O (77:23), 14.3 min, 99.9% purity.

#### N-(1-(2-acrylamido-4-(3-benzyl-1-((1r,4r)-4-((5-cyanopyridin-2-yl)amino)cyclohexyl)ureido)phenyl)piperidin-4-yl)-N-methyl-1-(5-((3aS,4S,6aR)-2-oxohexahydro-1H-thieno[3,4-d]imidazol-4-yl)pentanamido)-3,6,9,12-tetraoxapentadecan-15-amide (YJZ9149).

To a solution of **14m** (65 mg·0.1 mmol), HATU (48.8 mg·0.12 mmol), and DIPEA (20.7 mg, 0.16 mmol) in DMF (8 mL), was added 17-oxo-21-((*3aS,4S,6aR*)-2-oxohexahydro-1*H*-thieno[3,4-d]imidazol-4-yl)-4,7,10,13-tetraoxa-16-azahenicosanoic acid (58.3 mg, 0.12 mmol). The mixture was stirred at rt for 30 min. The reaction mixture was concentrated in vacuo and purified by column chromatography to give the desired compound **YJZ9149** as white solid (77 mg, yield 71%). ^1^H NMR (400 MHz, DMSO-*d*_6_) δ 9.06 (d, *J* = 7.0 Hz, 1H), 8.30 (d, *J* = 2.3 Hz, 1H), 7.96 (s, 1H), 7.88–7.78 (m, 1H), 7.60 (dd, *J* = 8.8, 2.3 Hz, 1H), 7.49 (d, *J* = 7.6 Hz, 1H), 7.26 (q, *J* = 9.2, 8.4 Hz, 3H), 7.21–7.15 (m, 3H), 6.91 (dt, *J* = 8.5, 2.9 Hz, 1H), 6.76 (dd, *J* = 17.0, 10.4 Hz, 1H), 6.47 (d, *J* = 8.9 Hz, 1H), 6.41 (s, 1H), 6.35 (s, 1H), 6.30 (dd, *J* = 16.9, 1.9 Hz, 1H), 5.84–5.76 (d, *J* = 11.3 Hz, 2H), 4.49–4.39 (m, 0.5H), 4.34–4.22 (m, 2H), 4.17 (d, *J* = 5.9 Hz, 2H), 4.15–4.10 (m, 1H), 3.90–3.80 (m, 0.5H), 3.65 (t, *J* = 6.8 Hz, 2H), 3.52–3.48 (m, 14H), 3.21–3.15 (m, 2H), 3.12–3.01 (m, 3H), 2.93 (s, 2H), 2.87–2.71 (m, 4H), 2.66 (t, *J* = 6.6 Hz, 1H), 2.61–2.55 (m, 2H), 2.06 (td, *J* = 7.4, 2.6 Hz, 3H), 1.92 (d, *J* = 11.6 Hz, 2H), 1.78 (d, *J* = 11.7 Hz, 2H), 1.72–1.57 (m, 2H), 1.57–1.38 (m, 5H), 1.40–1.09 (m, 7H). ^13^C NMR (151 MHz, DMSO-*d*_6_) δ 172.58, 170.41, 170.24, 163.64, 163.17, 159.70, 157.04, 153.58, 143.20, 141.72, 133.70, 133.04, 132.64, 128.49 (3C), 127.66, 127.38, 127.11 (3C), 126.70, 124.21, 121.01, 120.92, 119.60, 94.46, 70.27, 70.20, 70.18, 70.04, 69.64, 67.63, 67.27, 61.50, 59.66, 55.90, 54.23, 53.74, 51.92, 51.69, 50.31, 49.01, 43.93, 40.53, 38.91, 35.56, 34.20, 33.57, 31.78, 30.65, 30.07, 29.86, 29.50, 29.10, 28.67, 28.51, 27.53, 25.73. HRMS (ESI) for C_56_H_77_N_11_O_9_S [M+H]^+^, calcd: 1080.5699, found: 1080.5905. HPLC analysis: MeOH-H_2_O (70:30), 6.87 min, 99.0% purity.

### Cell Culture.

VCaP and 22RV1 cells were purchased from ATCC, and each cell line was cultured according to ATCC guidelines. VCaP cells were cultured in DMEM, GlutaMAX^™^, while 22RV1 cells were cultured in RPMI 1640 Medium (ATCC modification). Both media were supplemented with 10% fetal bovine serum (FBS), 100 U/mL penicillin, and 100 *μ*g/mL streptomycin (P/S). The cells were maintained at 37 °C in 5% CO_2_ incubator. Cell lines were tested for mycoplasma using the Lonza MycoAlert Kit following the manufacturer’s protocol.

### Western Blotting.

Generally, after incubation with the compounds for the indicated time, the tested cells were washed by cold PBS buffer (Gibco) and lysed with RIPA buffer (ThermoFisher Scientific) supplemented with cOmplete protease inhibitor cocktail tablets (Sigma-Aldrich). The cell lysate was treated with ultrasonic for 20s, following a centrifugation of 14000 rpm at 4°C for 10 min. The protein concentration was measured by BCA protein assay (DC Protein Assay, BIO-RAD). An equal amount of protein was loaded to 4–12% Bis-Tris gels and underwent SDS-PAGE electrophoresis at 120 V for 90 min. The proteins were transferred to a nitrocellulose (NC) membrane (Thermo Scientific) and the membranes were blocked in 5% milk buffer at room temperature for 1h, followed by incubating with primary antibody at the recommended dilution at 4°C for overnight. The membranes were washed by PBST for three times and incubated with anti-rabbit or anti-mouse HRP-conjugated secondary antibody for 1h at room temperature, after which the membranes were imaged by Odyssey CLx Imager (LiCOR Biosciences).

### *In Vitro* Kinase Enzyme Assay.

All the kinase activity were performed using an ADP-Glo kinase assay by ICE Bioscience Inc. (Beijing, China). Kinome selectivity profiles (KINOMEscan profiling) and *K*_d_ detection were performed by Eurofins DiscoveryX Corporation (San Diego, CA, USA).

### Protein Expression and Purification.

Protein used for crystallization was expressed in *Spodoptera frugiperda* cells (Sf9) transfected with codon-optimized synthetic genes comprising the kinase domain of human CDK12 (UniProt accession number Q9NYV4, residues 715–1052), the cyclin box domain of human Cyclin K (UniProt accession number O75909, residues 1–267), and full-length CAK1 from *Saccharomyces cerevisiae* (UniProt accession number P43568, residues 1–368). The expression and purification process were performed as previously described^8^.

### Crystallography.

The purified CDK12/CycK protein complex was stored in a buffer of 25 mM HEPES, pH 7.5, 150 mM NaCl and 1 mM TCEP. Prior to setting up crystallization trials, compounds diluted in DMSO was added in a 1:5 ratio to the pooled kinase complexes (10 mg/ml) and incubated for 30 minutes on ice, in order to generate a CDK12/CycK-compound ternary complex. Optimization on pH, buffer pairs, precipitant and ion concentration helped in growing big single crystals from the initial strip or sheet ones using the hanging drop vapor diffusion technique at 291 K by mixing the protein solution in 1:1 with the reservoir solution containing 0.1 M Bis-Tris, pH 5.8, 21.5% PEG 3350, 0.4 M MgCl_2_. The crystals were cryo-protected by rapid transfer into a reservoir solution supplemented with 20% glycerol and rapidly flash-frozen in ALS-style pucks that were submerged in liquid nitrogen. The diffraction data were collected at Protein Microcrystal Structure beamline 18U (Shanghai Synchrotron Radiation Facility, Shanghai) and processed using the XDS software package, pointless and aimless. The complex structure of compound **YJZ5118** with CDK12/CCNK was determined by molecular replacement using the program PHASER in CCP4 suite. Iterative cycle of refinement was carried out using COOT and PHENIX. Structure figures were created using PyMol.

### Real-Time Quantitative PCR Assay.

Total RNA was extracted using RNeasy Mini Kit (Qiagen). RNA concentrations were measured by NanoDrop 2000 spectrophotometer (Thermo Scientific). Reverse transcriptions and qRT-PCR assay were performed as previously described^14^.

### RNA-seq.

VCaP cells were treated by compound **YJZ5118** with the indicated concentrations. After 6 h incubation, RNA was extracted using RNeasy Mini Kit (Qiagen) and quantified by NanoDrop 2000 Spectrophotometers (Thermo Scientific) for RNA-seq as previously described^14^.

### Comet Assay.

Briefly, VCaP cells were treated with compound **YJZ5118** of 100 nM. After 12 h incubation, cells were collected and resuspended in ice-cold PBS. Following the protocol for OxiSelect Comet Assay Kit (Cell Biolabs, STA-351), the comets were observed using a fluorescence microscope and quantified using ImageJ. The data was analyzed using GraphPad Prism software.

### Pull-Down Assay.

Briefly, VCaP cells were treated with **YJZ9149** for 4 h, and then lysed in RIPA buffer (Thermo Fisher). The cell lysate was incubated with streptavidin beads overnight at 4 °C. The beads were washed with PBST buffer (0.1% Tween) for three times and eluted by 2X SDS loading buffer. The protein was then separated by SDS-PAGE gel electrophoresis.

### Cell Proliferation Assay.

Cells were seeded in 96-well plates and incubated at 37 °C and 5% CO_2_. After overnight incubation, serial dilutions of tested compounds were added to the plate. The assay was tested with Cell Titer-Glo (Promega) after 5 days. The luminescence signal was detected by the Infinite M1000 Pro plate reader (Tecan), and the IC_50_ values were calculated by GraphPad Prism 10.

### Cell Apoptosis Assay.

Cells were seeded in six-well plates and incubated for 24 h at 37 °C and 5% CO_2_. Cells were then treated with the indicated concentrations of the tested compounds for 48 h. The treated cells then were collected, centrifuged and washed twice by cold PBS. Cell apoptosis assay was performed using TUNEL Assay Kit (Cell Signaling Technology) by following the manufacturer’s instruction. Cell samples were analyzed with flow cytometry (SH800S cell sorter, Sony Biotechnology).

### Drug Synergism Assessment.

Briefly, 22RV1 cells were exposed to escalating concentrations of each drug for 5 days. Cell viability was determined post-treatment using the CellTiter-Glo Luminescent Cell Viability Assay (Promega) with three biological replicates. Results were calculated as percentage inhibition relative to control. The assessment of synergy was conducted using the Bliss method in SynergyFinder.

### IncuCyte Proliferation Assays.

Cell proliferation was quantitatively assessed using the IncuCyte Live-Cell Analysis System (Essen Bioscience). 22RV1 cells were plated in 96-well plates. Following overnight incubation at 37 °C and 5% CO_2_, the cells were treated with **YJZ5118** at the concentration of 70 nM, with or without AKT inhibitors. Real-time cell proliferation was monitored by capturing phase-contrast images every 4 h using a 10× objective. The IncuCyte software (version 2022A Rev1) was utilized to measure cell confluence continuously as a proxy for proliferation. Data analysis was performed using the software’s built-in analytical tools, focusing on growth curves and confluence metrics (percentage area). Figures were generated using GraphPad Prism software.

### Pharmacokinetics Study.

The pharmacokinetic investigation was taken by Shanghai Medicilon Inc. (Project Code: 10017–20023). Specific pathogen free male and female ICR mice provided by Shanghai Xipuer-BK Laboratory Animal Co., Ltd were dosed with tested compounds solution formulation (5% DMSO, 10% Solutol, 85% normal saline, 2.5 or 2 mg/kg for intravenous dose, 10 mg/kg for oral dose). Blood samples were collected at 0.083, 0.25, 0.5, 1, 2, 4, 8 and 24 h in intravenous administration, 0.25, 0.5, 1, 2, 4, 6, 8, and 24 h in oral administration. The blood samples were collected from sets of three mice at each time point in labeled microcentrifuge tubes containing heparin sodium as an anticoagulant. Plasma samples were separated by centrifugation (2–8 °C, 6800 g for 6 min) within 1 h and stored below −80 °C until bioanalysis. All samples were processed for analysis by precipitation using acetonitrile and analyzed with a partially validated LC/MS/MS method. Pharmacokinetic parameters were calculated using the noncompartmental analysis tool of WinNonlin Enterprise software.

### *In Vivo* Efficacy Study.

All the animal experiments were performed under an approved animal protocol (Protocol ID: PRO00010006, PI, Arul Chinnaiyan) by the Institutional Animal Care & Use Committee of the University of Michigan. Six- to eight-week-old NSG (Jackson Laboratory) or CB17SCID female (Charles River Laboratory) mice were in a regular SPF housing room prior to cell injection. Briefly, 5 ×10^6^ cells of VCaP CRPC were injected orthotopically into the mammary fat pad of NSG or CB17SCID mice, respectively. After tumor size reached approximately 200–400 mm^3^, animals were subjected to drug treatment. 0.5 or 1.5 mg/kg of compound **YJZ5118** was administered to animals by i.p. injection for 27 days. Vehicle consisted of 20% PEG400, 6% Cremophor EL, and 74% PBS solution. Tumors were collected at the end of the experiment for Western blot analysis.

### Histology Analysis.

H&E staining of formalin-fixed paraffin embedded (FFPE) tissue sections and histological assessment was performed as previously described^14^.

### Computational Modeling Studies.

The structure of CDK12 (PDB: 5ACB) was prepared using Protein Preparation Wizard and the compounds were prepared by LigPrep. The covalent docking was carried out using covalent docking module with default settings (Schrödinger, LLC, New York, NY, 2021).

## Supplementary Material

Supporting

ASSOCIATED CONTENT

Supporting Information

The selectivity profiling results of compound **14h**; PK profiles of compound **14h and YJZ5118**; Caco-2 permeability results of compounds **14a** and **14b**; Washout results for **YJZ5118**; Real-time growth curves of VCaP cells upon treatment with **YJZ5118**; Atomic coordinates and experimental data for the co-crystal structure of compound **YJZ5118** with CDK12/CCNK; NMR spectra and HPLC chromatograms for all final compounds;

Molecular formula strings (CSV);

Docking pose of compound **2** in CDK12 (PDB);

Docking pose of compound **14a** in CDK12 (PDB);

## Figures and Tables

**Table 1.** *In Vitro* CDK12 and CDK13 Kinase Inhibitory Activity and Cellular Antiproliferative Potency of Compounds 14a–k.

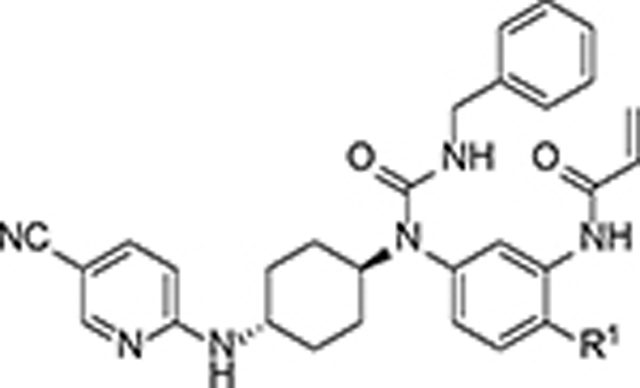				

Cpd.	R^1^	IC_50_ (nM)		
		CDK12^a^	CDK13^a^	VCaP

**3**	-	77.4 (158^b^)	63.3 (69^b^)	33.5
**2**	-	28.6 (52^c^)	17.8 (10^c^)	1950
**14a**	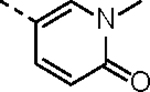	37.9	14.9	330.4
**14b**	H	506.0	99.0	96.2
**14c**	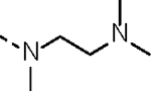	190.6	79.3	44.4
**14d**	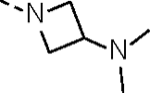	151.3	73.7	133.3
**14e**	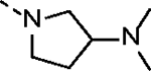	567.5	176.8	57.6
**14f**	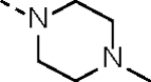	130.3	66.8	39.9
**14g**	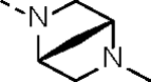	209.1	83.9	76.3
**14h**	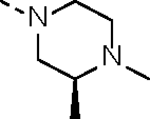	33.1	21.7	4.3
**14i**	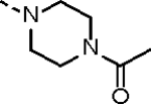	331.3	164	45.5
**14j**	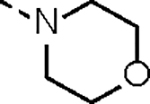	55.0	12.8	59.9
**14k (YJZ5118)**	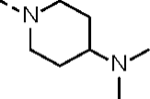	39.5	26.4	23.7

a CDK12/13 inhibition was performed by using an ADP-Glo kinase assay. The data are means from three independent experiments.

b The reported IC_50_ values for CDK12/13 determined by using a radioactive kinase activity assay.^17^

c The reported IC_50_ values for CDK12/13 determined by using a LANCE Ultra assay.^16^

## ABBREVIATIONS

CDK12/13: Cyclin-dependent kinases 12 and 13
CPRC: castration-resistant prostate cancer
KD: kinase domain
CCNK: cyclin K
CTD: C-terminal domain
DDR: DNA damage response
PCa: prostate cancer
mCRPC: metastatic castration-resistant prostate cancer
PK: pharmacokinetics
IC_50_: half inhibition concentration
Xantphos: 4,5-bis(diphenylphosphino)-9,9-dimethylxanthene
*tert*-BuONa: sodium *tert*-butoxide
Pd_2_(dba)_3_: tris(dibenzylideneacetone) dipalladium
DIPEA: *N,N*-diisopropylethylamine
DMF: *N,N*-dimethylformamide
TFA: trifluoroacetic acid
DCM: dichloromethane
Cs_2_CO_3_: cesium carbonate
K_2_CO_3_: potassium carbonate
K_3_PO_4_: tripotassium phosphate
Pd(pph_3_)_4_: Tetrakis(triphenylphosphine)palladium
T_1/2_: terminal half-life
C_max_: maximum drug plasma concentration
AUC: area under the drug concentration-time curve
CL: plasma clearance rate
F: oral bioavailability
PARP: Poly ADP-ribose polymerase
PCPA: polyadenylation
RNA-seq: RNA sequencing
5-EU: 5-ethynyluridine
i.v.: intravenous administration
i.p.: intraperitoneal injection
HATU: 2-(7-azabenzotriazol-1-yl)-*N*′,*N*′,*N*′-tetramethyluronium hexafluorophosphate
TLC: thin-layer chromatography
TUNEL: terminal dUTP nick end labeling
IHC: immunohistochemistry
