# Supplementary material for: Discovery of YJZ5118: a Potent and Highly Selective Irreversible CDK12/13 Inhibitor with Synergistic Effects in Combination with Akt Inhibition
Source: J Med Chem. Author manuscript; Available in PMC 2025 Jun 9. (PMC12147761; doi:10.1021/acs.jmedchem.5c00127)
Supplement: Supporting [file NIHMS2082140-supplement-Supporting.pdf]

Supporting Information

Discovery of YJZ5118: a Potent and Highly Selective Irreversible CDK12/13 Inhibitor with Synergistic Effects in Combination with Akt Inhibition

Jianzhang Yang, [α, β, #] Yu Chang, [δ, #] Kaijie Zhou, [α, #] Weixue Huang, [α, #] Jean Ching-Yi Tien, [δ, Ψ] Pujuan Zhang, [α] Wenyan Liu, [α, δ] Licheng Zhou, [β] Yang Zhou, [β] Xiaomei Ren, [α] Rahul Mannan, [δ] Somnath Mahapatra,[δ] Yuping Zhang,[δ] Rudana Hamadeh,[δ] Grafton Ervine, [δ] Zhen Wang, [α, *] George Xiaoju Wang, [δ, Ψ, *] Arul M. Chinnaiyan, [δ, Ψ, Φ, χ, ξ, *] and Ke Ding [α, *]

[α] State Key Laboratory of Chemical Biology, Shanghai Institute of Organic Chemistry, Chinese Academy of Sciences, #345 Lingling Road, Shanghai 200032, China

[β] International Cooperative Laboratory of Traditional Chinese Medicine Modernization and Innovative Drug Discovery of Chinese Ministry of Education (MOE), Guangzhou City Key Laboratory of Precision Chemical Drug Development, College of Pharmacy, Jinan University, 855 Xingye Avenue East, Guangzhou 511400, China

[δ] Michigan Center for Translational Pathology, University of Michigan, Ann Arbor, Michigan 48109, USA

[Ψ] Department of Pathology, University of Michigan, Ann Arbor, MI 48109, USA

[Φ] Howard Hughes Medical Institute, University of Michigan, Ann Arbor, MI 48109, USA

[χ] Rogel Cancer Center, University of Michigan, Ann Arbor, MI 48109, USA

[ξ] Department of Urology, University of Michigan, Ann Arbor, MI 48109, USA

[#]J.Y., Y.C., K.Z., and W.H. contributed equally to this work.

[*]wangz@sioc.ac.cn (Z.W.); xiaojuw@med.umich.edu (X.W.); arul@med.umich.edu (A.M.C.); dingk@sioc.ac.cn, Tel: +86-21-5492 5100 (K.D.)

Contents

The Results of Selectivity Profiling Study of Compound **14h**	S3-7
The Results of the Pharmacokinetics Study of Compound **14h**	S7
Permeability and Efflux Ratio Determination of Compounds **14a** and **14b** in Caco-2 Cells.	S8
Kinase Inhibition Activity Detection of **YJZ5118** against TAOK3 by ADP-Glo Assay.	S8
Comparisons of Anti-tumor Cell Effects between **YJZ5118** and the Reversible Inhibitor **2**	S9
PK Parameters of Compound **YJZ5118** by Intraperitoneal Administration in Mice.	S9
Data Collection and Refinement Statistics of Compound **YJZ5118** with CDK12/CCNK	S10
The ^{1}H NMR, ^{13}C NMR, HRMS and HPLC Traces of Compounds **14a-k**, and **YJZ9149**	S11-34

A

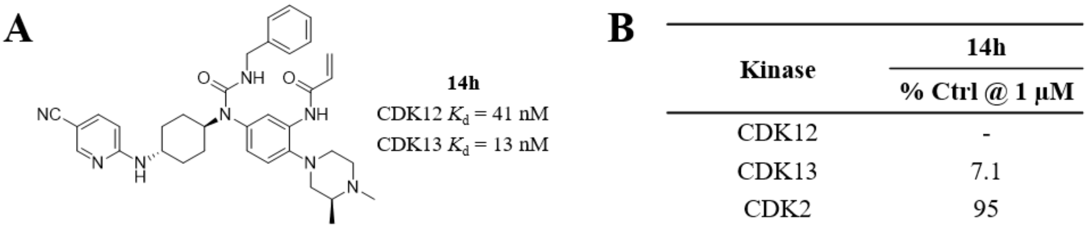

14h
CDK12 K_d = 41 nM
CDK13 K_d = 13 nM

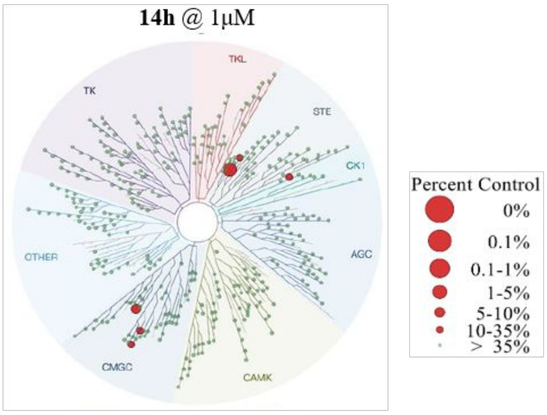

B

**Kinase**	**14h**
	**% Ctrl @ 1 µM**
CDK12	-
CDK13	7.1
CDK2	95
CDK3	88
CDK4	100
CDK4-cyclinD1	89
CDK4-cyclinD3	100
CDK5	84
CDK7	38
CDK8	88
CDK9	94
CDK11	85
CDK11A	95
CDK11B	93

Figure S1. The results of selectivity profiling study of compound **14h**. (A) Chemical structure and the K_d values of compound **14h** against CDK12/13, and the KinomeScan kinase selectivity profiles of compound **14h** at 1 µM against 468 kinases; (B) KinomeScan profiling results of compound **14h** against CDK family members.

Table S1. Matrix of Compound Screen Results for Inhibitor **14h** (**YJZ6093**).

Target	YJZ6093
Gene Symbol	%Ctrl @ 1000nM
AAK1	94
ABL1(E255K)-phosphorylated	87
ABL1(F317I)-nonphosphorylated	100
ABL1(F317I)-phosphorylated	100
ABL1(F317L)-nonphosphorylated	92
ABL1(F317L)-phosphorylated	100
ABL1(H396P)-nonphosphorylated	92
ABL1(H396P)-phosphorylated	89
ABL1(M351T)-phosphorylated	100
ABL1(Q252H)-nonphosphorylated	83
ABL1(Q252H)-phosphorylated	99
ABL1(T315I)-nonphosphorylated	100
ABL1(T315I)-phosphorylated	100
ABL1(Y253F)-phosphorylated	87
ABL1-nonphosphorylated	66
ABL1-phosphorylated	85
ABL2	92
ACVR1	84
ACVR1B	90
ACVR2A	75
ACVR2B	63
ACVRL1	97
ADCK3	95
ADCK4	100
AKT1	68
AKT2	94
AKT3	100
ALK	100
ALK(C1156Y)	100
ALK(L1196M)	100
AMPK-alpha1	83
AMPK-alpha2	68
ANKK1	100
ARK5	86
ASK1	87
ASK2	100
AURKA	95
AURKB	100
AURKC	100
AXL	94
BIKE	94
BLK	95
BMPR1A	91
BMPR1B	100
BMPR2	94
BMX	97
BRAF	100
BRAF(V600E)	100
BRK	88
BRSK1	99
BRSK2	99
BTK	100
BUB1	88
CAMK1	79
CAMK1B	100
CAMK1D	69
CAMK1G	93
CAMK2A	100
CAMK2B	84
CAMK2D	84
CAMK2G	90
CAMK4	100
CAMKK1	86
CAMKK2	77
CASK	100
CDC2L1	95
CDC2L2	93
CDC2L5	7.1
CDK11	85
CDK2	95
CDK3	88
CDK4	100
CDK4-cyclinD1	89
CDK4-cyclinD3	100
CDK5	84
CDK7	38
CDK8	88
CDK9	94
CDKL1	76
CDKL2	88
CDKL3	100
CDKL5	100
CHEK1	94
CHEK2	85
CIT	95
CLK1	80
CLK2	93
CLK3	94
CLK4	91
CSF1R	88
CSF1R-autoinhibited	100
CSK	74
CSNK1A1	51
CSNK1A1L	64
CSNK1D	67
CSNK1E	50
CSNK1G1	78
CSNK1G2	38
CSNK1G3	22
CSNK2A1	100
CSNK2A2	83
CTK	100
DAPK1	86
DAPK2	85
DAPK3	55
DCAMKL1	100
DCAMKL2	90
DCAMKL3	85
DDR1	82
DDR2	100
DLK	100
DMPK	89
DMPK2	98
DRAK1	100
DRAK2	89
DYRK1A	100
DYRK1B	78
DYRK2	90
EGFR	100
EGFR(E746-A750del)	91
EGFR(G719C)	96
EGFR(G719S)	95
EGFR(L747-E749del, A750P)	97
EGFR(L747-S752del, P753S)	93
EGFR(L747-T751del,Sins)	88
EGFR(L858R)	99
EGFR(L858R,T790M)	100
EGFR(L861Q)	100
EGFR(S752-I759del)	86
EGFR(T790M)	95
EIF2AK1	100
EPHA1	85
EPHA2	84
EPHA3	97
EPHA4	77
EPHA5	87
EPHA6	99
EPHA7	96
EPHA8	90
EPHB1	92
EPHB2	100
EPHB3	90
EPHB4	92
EPHB6	100
ERBB2	100
ERBB3	50

Target	YJZ6093
Gene Symbol	%Ctrl @ 1000nM
ERBB4	95
ERK1	94
ERK2	88
ERK3	70
ERK4	94
ERK5	89
ERK8	74
ERN1	92
FAK	91
FER	100
FES	90
FGFR1	92
FGFR2	84
FGFR3	96
FGFR3(G697C)	86
FGFR4	95
FGR	86
FLT1	94
FLT3	100
FLT3(D835H)	99
FLT3(D835V)	100
FLT3(D835Y)	59
FLT3(ITD)	82
FLT3(ITD,D835V)	100
FLT3(ITD,F691L)	100
FLT3(K663Q)	100
FLT3(N841I)	94
FLT3(R834Q)	100
FLT3-autoinhibited	100
FLT4	99
FRK	87
FYN	88
GAK	94
GCN2(Kin.Dom.2,S808G)	74
GRK1	100
GRK2	100
GRK3	100
GRK4	62
GRK7	87
GSK3A	60
GSK3B	100
HASPIN	100
HCK	86
HIPK1	76
HIPK2	100
HIPK3	100
HIPK4	68
HPK1	100
HUNK	99

Target	YJZ6093
Gene Symbol	%Ctrl @ 1000nM
ICK	100
IGF1R	94
IKK-alpha	100
IKK-beta	100
IKK-epsilon	100
INSR	95
INSRR	81
IRAK1	91
IRAK3	92
IRAK4	89
ITK	83
JAK1(JH1domain-catalytic)	93
JAK1(JH2domain-pseudokinase)	94
JAK2(JH1domain-catalytic)	95
JAK3(JH1domain-catalytic)	100
JNK1	62
JNK2	28
JNK3	21
KIT	86
KIT(A829P)	100
KIT(D816H)	100
KIT(D816V)	90
KIT(L576P)	85
KIT(V559D)	78
KIT(V559D,T670I)	75
KIT(V559D,V654A)	86
KIT-autoinhibited	100
LATS1	100
LATS2	100
LCK	90
LIMK1	93
LIMK2	96
LKB1	100
LOK	82
LRRK2	100
LRRK2(G2019S)	100
LTK	100
LYN	100
LZK	100
MAK	78
MAP3K1	100
MAP3K15	93
MAP3K2	100
MAP3K3	84
MAP3K4	77
MAP4K2	100
MAP4K3	88
MAP4K4	89
MAP4K5	100

Target	YJZ6093
Gene Symbol	%Ctrl @ 1000nM
MAPKAPK2	59
MAPKAPK5	100
MARK1	96
MARK2	92
MARK3	100
MARK4	100
MAST1	99
MEK1	96
MEK2	97
MEK3	100
MEK4	100
MEK5	100
MEK6	89
MELK	85
MERTK	86
MET	86
MET(M1250T)	95
MET(Y1235D)	100
MINK	100
MKK7	100
MKNK1	100
MKNK2	100
MLCK	95
MLK1	87
MLK2	90
MLK3	94
MRCKA	100
MRCKB	95
MST1	91
MST1R	90
MST2	100
MST3	84
MST4	100
MTOR	100
MUSK	84
MYLK	95
MYLK2	80
MYLK4	97
MYO3A	88
MYO3B	92
NDR1	100
NDR2	89
NEK1	83
NEK10	100
NEK11	100
NEK2	97
NEK3	98
NEK4	100
NEK5	89

Target	YJZ6093
Gene Symbol	%Ctrl @ 1000nM
NEK6	86
NEK7	91
NEK9	96
NIK	100
NIM1	100
NLK	79
OSR1	100
p38-alpha	94
p38-beta	93
p38-delta	86
p38-gamma	83
PAK1	100
PAK2	100
PAK3	94
PAK4	100
PAK6	89
PAK7	80
PCTK1	100
PCTK2	93
PCTK3	91
PDGFRA	100
PDGFRB	89
PDPK1	52
PFCDPK1(P.falciparum)	100
PFPK5(P.falciparum)	100
PFTAIRE2	84
PFTK1	74
PHKG1	95
PHKG2	67
PIK3C2B	90
PIK3C2G	86
PIK3CA	81
PIK3CA(C420R)	78
PIK3CA(E542K)	100
PIK3CA(E545A)	91
PIK3CA(E545K)	99
PIK3CA(H1047L)	100
PIK3CA(H1047Y)	83
PIK3CA(I800L)	100
PIK3CA(M1043I)	100
PIK3CA(Q546K)	97
PIK3CB	100
PIK3CD	97
PIK3CG	94
PIK4CB	100
PIKFYVE	36
PIM1	93
PIM2	80
PIM3	99

Target	YJZ6093
Gene Symbol	%Ctrl @ 1000nM
PIP5K1A	89
PIP5K1C	48
PIP5K2B	78
PIP5K2C	66
PKAC-alpha	73
PKAC-beta	100
PKMYT1	63
PKN1	98
PKN2	100
PKNB(M.tuberculosis)	99
PLK1	100
PLK2	100
PLK3	98
PLK4	86
PRKCD	95
PRKCE	97
PRKCH	75
PRKCI	91
PRKCQ	92
PRKD1	87
PRKD2	91
PRKD3	97
PRKG1	99
PRKG2	100
PRKR	79
PRKX	87
PRP4	80
PYK2	90
QSK	100
RAF1	100
RET	84
RET(M918T)	85
RET(V804L)	91
RET(V804M)	94
RIOK1	87
RIOK2	97
RIOK3	99
RIPK1	88
RIPK2	85
RIPK4	77
RIPK5	88
ROCK1	100
ROCK2	100
ROS1	100
RPS6KA4(Kin.Dom.1-N-terminal)	90
RPS6KA4(Kin.Dom.2-C-terminal)	96
RPS6KA5(Kin.Dom.1-N-terminal)	90
RPS6KA5(Kin.Dom.2-C-terminal)	93
RSK1(Kin.Dom.1-N-terminal)	84

Target	YJZ6093
Gene Symbol	%Ctrl @ 1000nM
RSK1(Kin.Dom.2-C-terminal)	88
RSK2(Kin.Dom.1-N-terminal)	100
RSK2(Kin.Dom.2-C-terminal)	100
RSK3(Kin.Dom.1-N-terminal)	97
RSK3(Kin.Dom.2-C-terminal)	79
RSK4(Kin.Dom.1-N-terminal)	100
RSK4(Kin.Dom.2-C-terminal)	72
S6K1	100
SBK1	100
SGK	100
SgK110	74
SGK2	100
SGK3	97
SIK	81
SIK2	100
SLK	88
SNARK	100
SNRK	100
SRC	100
SRMS	100
SRPK1	95
SRPK2	98
SRPK3	100
STK16	70
STK33	99
STK35	96
STK36	96
STK39	57
SYK	89
TAK1	75
TAOK1	25
TAOK2	84
TAOK3	3.5
TBK1	72
TEC	98
TESK1	95
TGFBR1	82
TGFBR2	92
TIE1	94
TIE2	84
TLK1	81
TLK2	79
TNIK	86
TNK1	80
TNK2	89
TNNI3K	76
TRKA	100
TRKB	100
TRKC	100

Target	YJZ6093
Gene Symbol	%Ctrl @ 1000nM
TRPM6	100
TSSK1B	95
TSSK3	100
TTK	45
TXK	87
TYK2(JH1domain-catalytic)	100
TYK2(JH2domain-pseudokinase)	100
TYRO3	93
ULK1	98
ULK2	98
ULK3	100
VEGFR2	100
VPS34	100
VRK2	100
WEE1	84
WEE2	90
WNK1	96
WNK2	100
WNK3	94
WNK4	88
YANK1	100
YANK2	100
YANK3	88
YES	81
YSK1	99
YSK4	100
ZAK	93
ZAP70	100

%Ctrl Legend

0≤x<.1 | .1≤x<1 | 1≤x<10 | 10≤x<35 | x≥35

Table S2. S-score Table for **14h** (1 μM).

Compound	Selectivity Score Type	Number of Hits	Number of Non-Mutant Kinases	Selectivity Score
	S (35)	6	403	0.015
**14h**	S (10)	2	403	0.005
	S (1)	0	403	0

Table S3. PK Profiles of Compound **14h** in Mice.

Compound	Route	$T_{1/2}$ (h)	T_{max} (h)	C_{max} (ng/mL)	AUC (0-t) (h*ng/mL)	CL (mL/min/kg)	F (%)
**14h**	i.v. 2 mg/kg	0.36	0.08	657.9	309.2	107.6	
	p.o. 10 mg/kg	0.62	0.50	499.2	652.2	-	42.18

Table S4. Permeability and Efflux Ratio Determination of Compounds **14a** and **14b** in Caco-2 Cells.

Compounds	Papp (10^{-6} cm/s)		Efflux Ratio
	A to B	B to A	
**14a**	0.07	5.25	72.58
**14b**	0.91	13.72	15.00

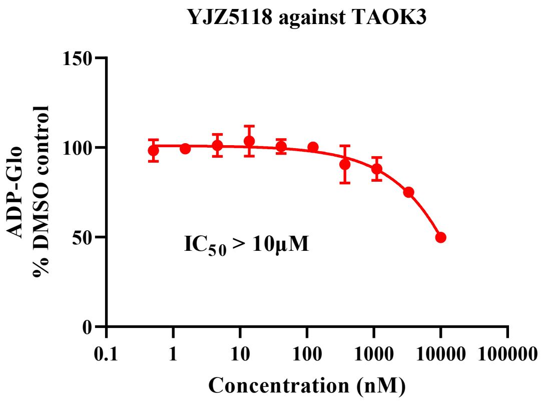

Figure S2. Kinase inhibition activity detection of **YJZ5118** against TAOK3 by ADP-Glo assay.

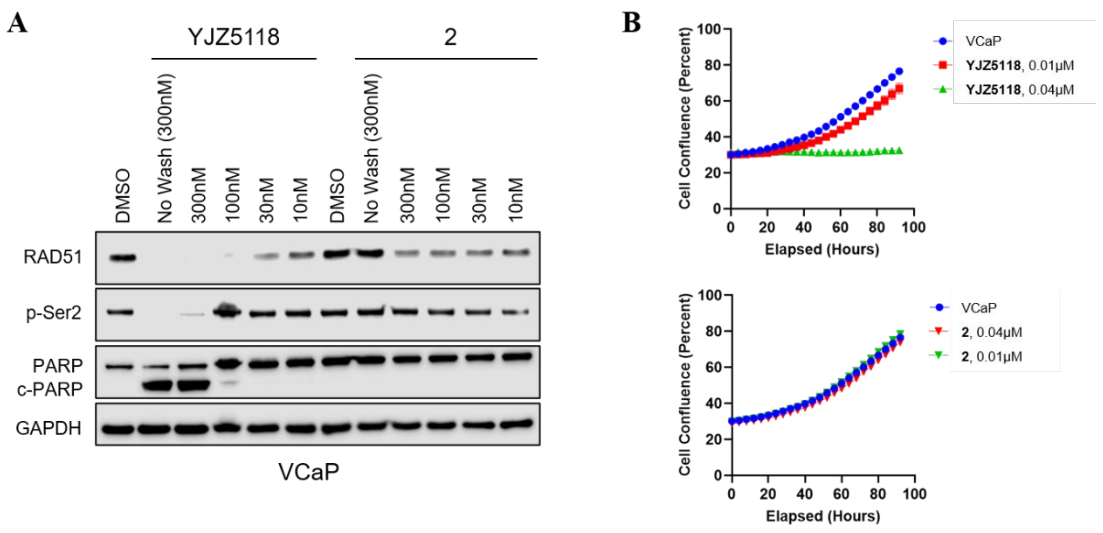

Figure S3. (A) Washout experiments using compound **YJZ5118** and reversible inhibitors **2** in VCaP cells. Cells were pretreated with compounds for 2 h, then washout and further incubated for 12 h. GAPDH is used as a loading control. (B) Real-time growth curves of VCaP cells upon treatment with compound **YJZ5118** or reversible inhibitors **2** at different concentrations.

Table S5. PK Parameters of Compound **YJZ5118** by Intraperitoneal Administration in Mice.

Compound	Route	$T_{1/2}$ (h)	T_{max} (h)	C_{max} (ng/mL)	AUC (0-t) (h*ng/mL)	CL (mL/min/kg)	F (%)
**YJZ5118**	i.v. 2.5 mg/kg	1.21	0.08	3313.77	1657.52	25.58	
	i.p. 2.5 mg/kg	1.46	0.33	1583.30	1857.69	-	113.16

Table S6. Data collection and refinement statistics (molecular replacement)

	CDK12/Cyclin K/**YJZ5118**
PDB ID	9JK1
**Data collection**	
Wavelength(Å)	0.97915
Space group	*P*1
Cell dimensions	
a, *b*, *c* (Å)	50.2, 77.19, 91.46
α, β, γ (°)	75.88, 85.63, 77.92
Resolution (Å)	29.56 - 2.72 (2.82 - 2.72) *
R_{merge}	0.079 (0.737)
$I / \sigma (I)$	11.18 (1.92)
$CC^{1/2}$	0.997 (0.670)
Completeness (%)	99.42 (97.30)
Redundancy	3.5 (3.6)
**Refinement**	
Resolution (Å)	29.56 - 2.72
No. Unique reflections	34065（3385）
R_{work} / R_{free}	0.2483/0.2665
No. atoms	8709
Protein	8584
Ligand/ion	120
Water	5
B-factors	68.69
Protein	68.51
Ligand/ion	82.06
Water	56.64
R.m.s. deviations	
Bond lengths (Å)	0.004
Bond angles (°)	0.71
Ramachandran plot	
Most favored region (%)	95.99
Allowed regions (%)	3.73
Disallowed regions (%)	0.28

*Values in parentheses are for highest-resolution shell.

The ^{1}H NMR, ^{13}C NMR, HRMS and HPLC traces of Compounds 14a-k, and YJZ9149.

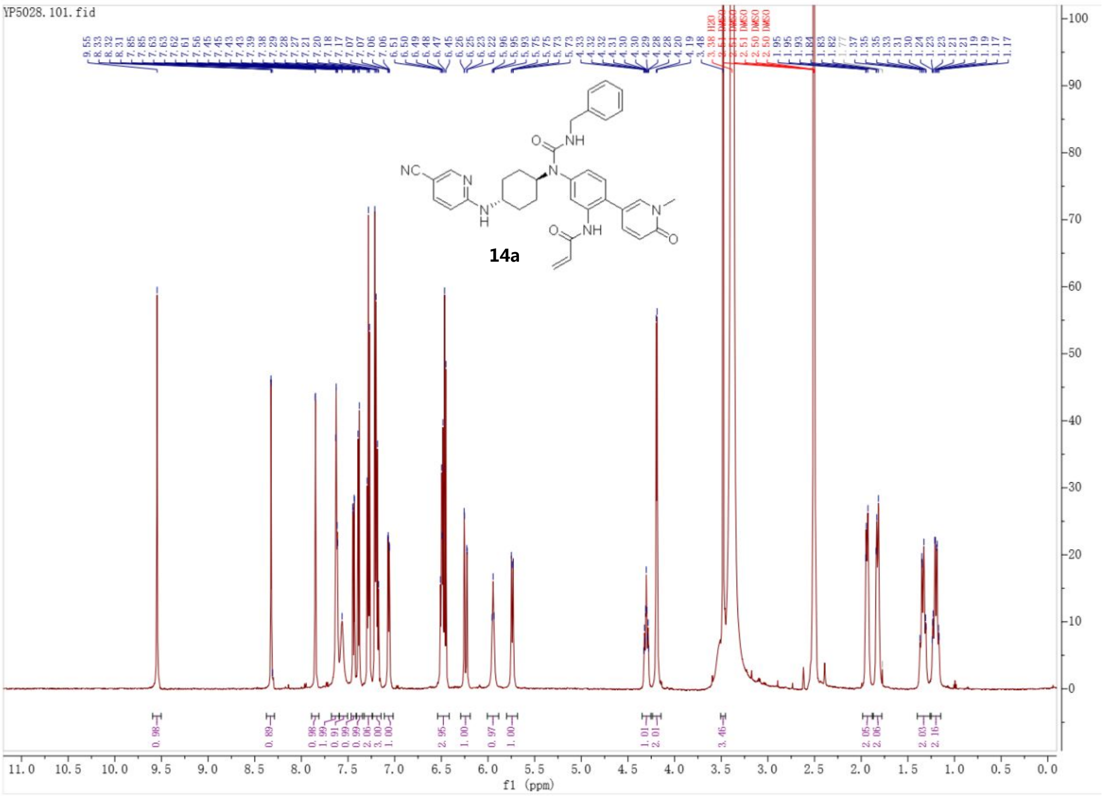

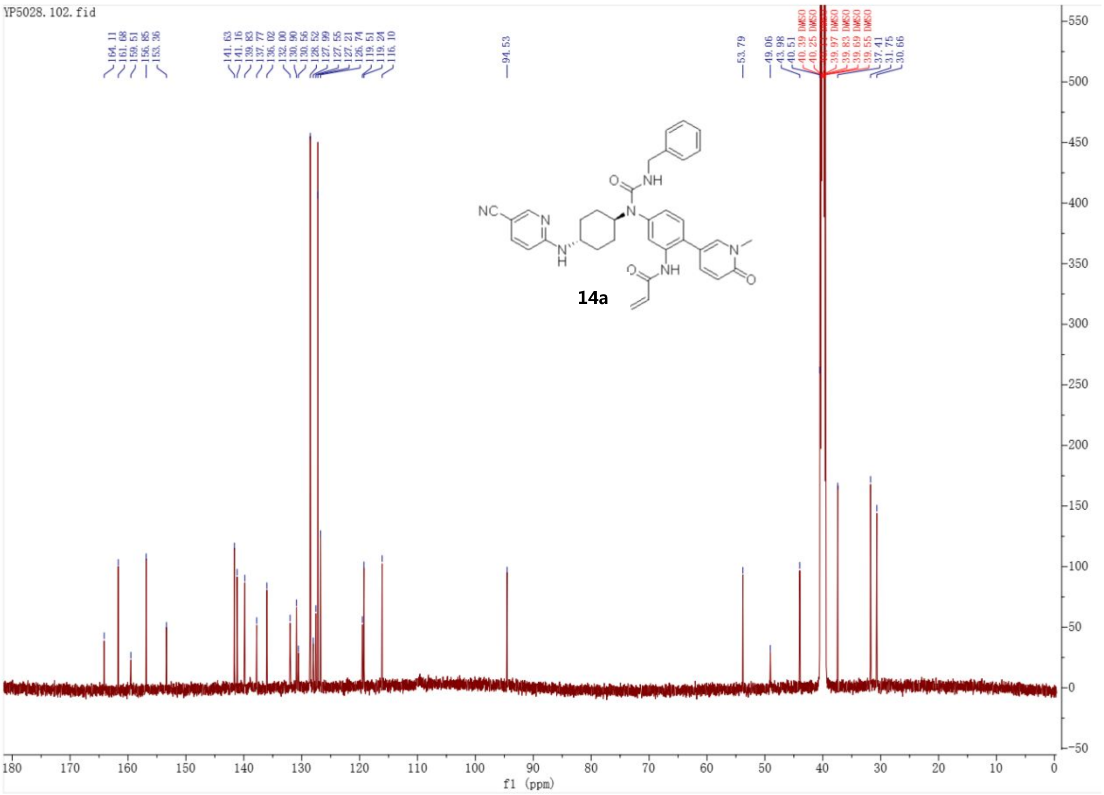

Hit	Formula	m/z	RDB	ppm	MS Rank	MSMS ppm	MSMS Rank	Found
1	C35H35N7O3	602.2874	22.0	-3.5	1			NA/NA

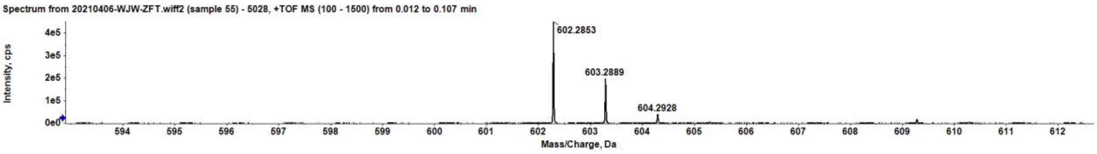


```
ata File E:\DK\YJZ\data\20220422\DELL-PC15076.D
ample Name: 5028

=====================================================================
Acq. Operator   : 系统
Sample Operator : 系统
Acq. Instrument : 1260LC                    Location :   1
Injection Date  : 22/04/2022 14:50:33
                                        Inj Volume : 5.000 µl
Acq. Method     : E:\DK\TL\方法\70C-30D-40min-1u.M
Last changed    : 22/04/2022 14:49:12 by 系统
                  (modified after loading)
Analysis Method : E:\DK\TL\方法\85C-15A-30min-1u.M
Last changed    : 23/06/2021 10:52:58 by 系统
```

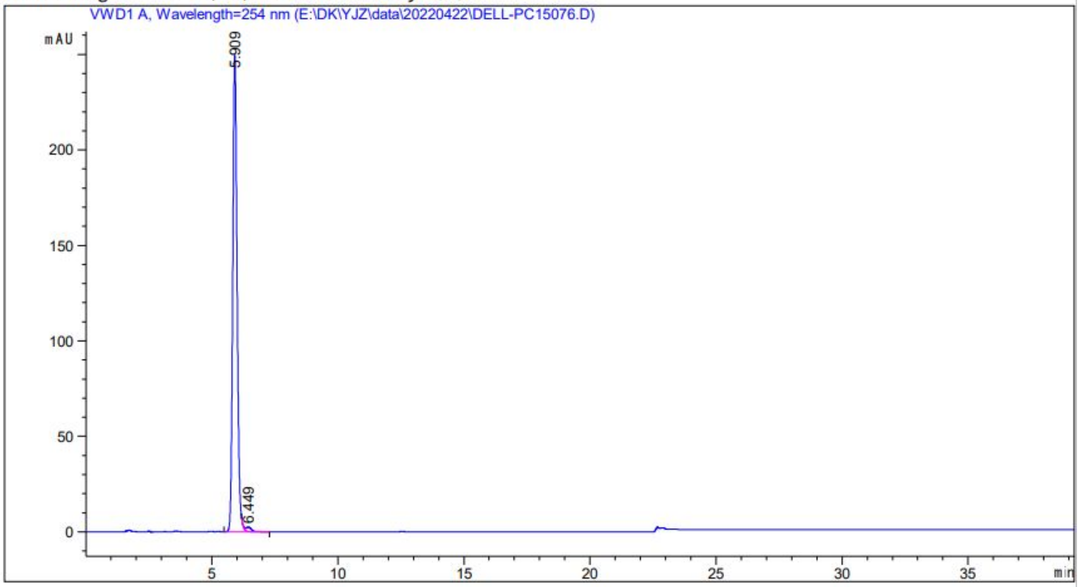


```
=====================================================================
                         Area Percent Report
=====================================================================

Sorted By             :      Signal
Multiplier            :      1.0000
Dilution              :      1.0000
Sample Amount:               :      20.00000  [ng/ul]   (not used in calc.)
Use Multiplier & Dilution Factor with ISTDs


Signal 1: VWD1 A, Wavelength=254 nm

Peak RetTime Type  Width     Area      Height     Area
  #   [min]        [min]   [mAU*s]     [mAU]        %
----|-------|----|-------|----------|----------|--------|
   1   5.909 BV R  0.1858 3011.96924  249.62656  98.7172
   2   6.449 VB E  0.2364   39.13809    2.34923   1.2828
```

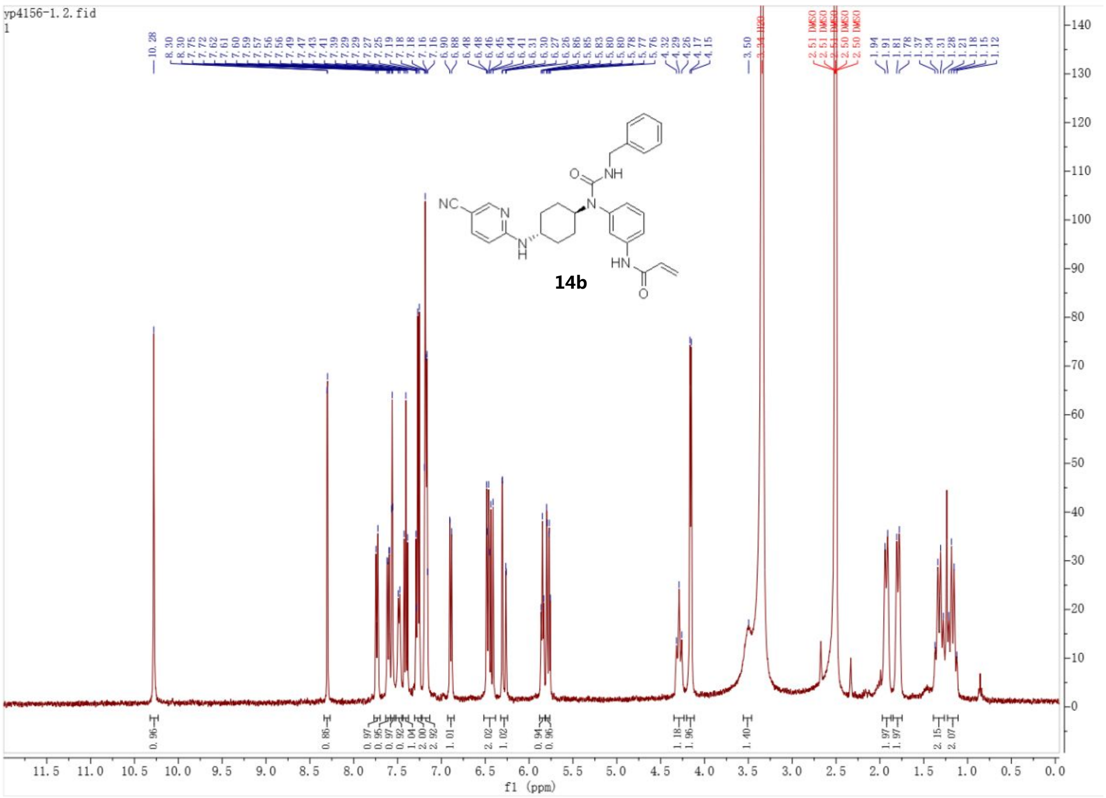

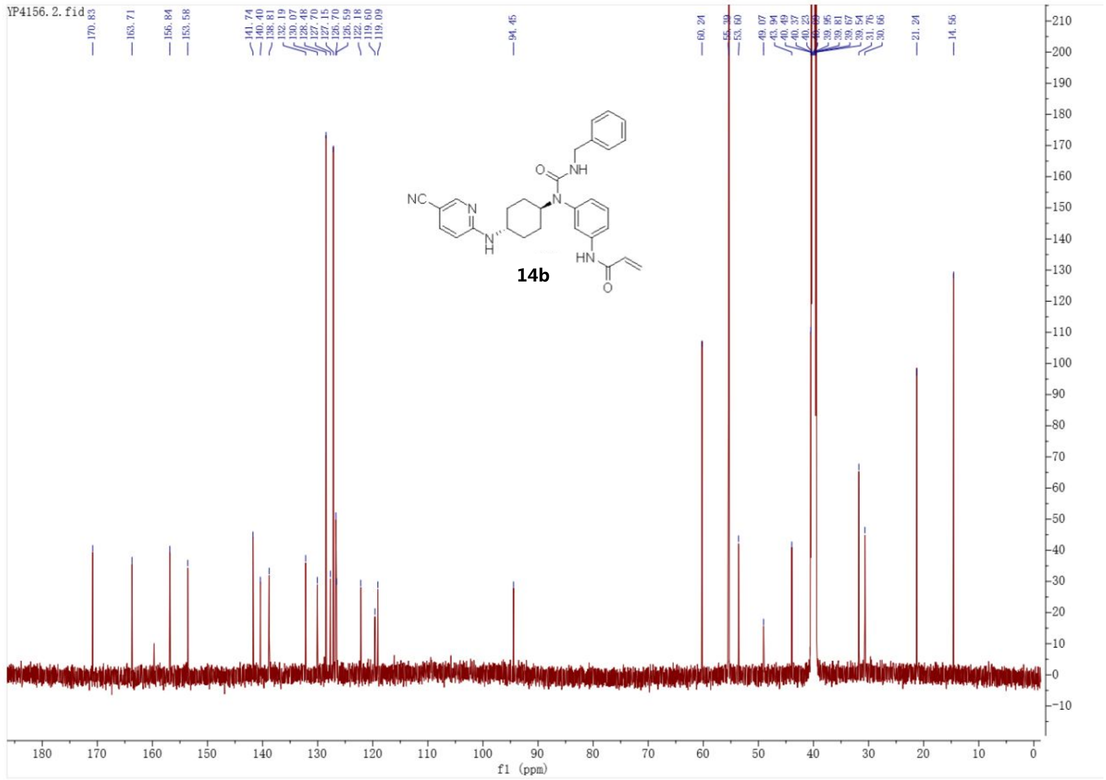

Hit	Formula	m/z	RDB	ppm	MS Rank	MSMS ppm	MSMS Rank	Found
1	C29H30N6O2	495.2503	18.0	-3.6	1			NA/NA

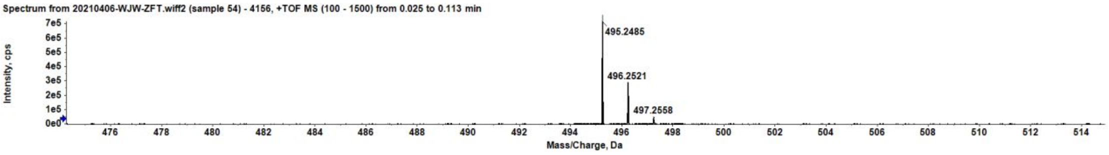

Data File E:\DK\YJZ\data\20220622\17938.D
Sample Name: 4156

===

Acq. Operator : 系统
Sample Operator : 系统
Acq. Instrument : 1260LC Location : 2
Injection Date : 22/06/2022 14:09:35
Inj Volume : 10.000 µl
Acq. Method : E:\DK\TL\方法\70C-30D-30min-1u.M
Last changed : 22/06/2022 14:08:50 by 系统
(modified after loading)
Analysis Method : E:\DK\TL\方法\90C-10D-30min-1u.M
Last changed : 04/03/2023 17:45:43 by 系统
Additional Info : Peak(s) manually integrated

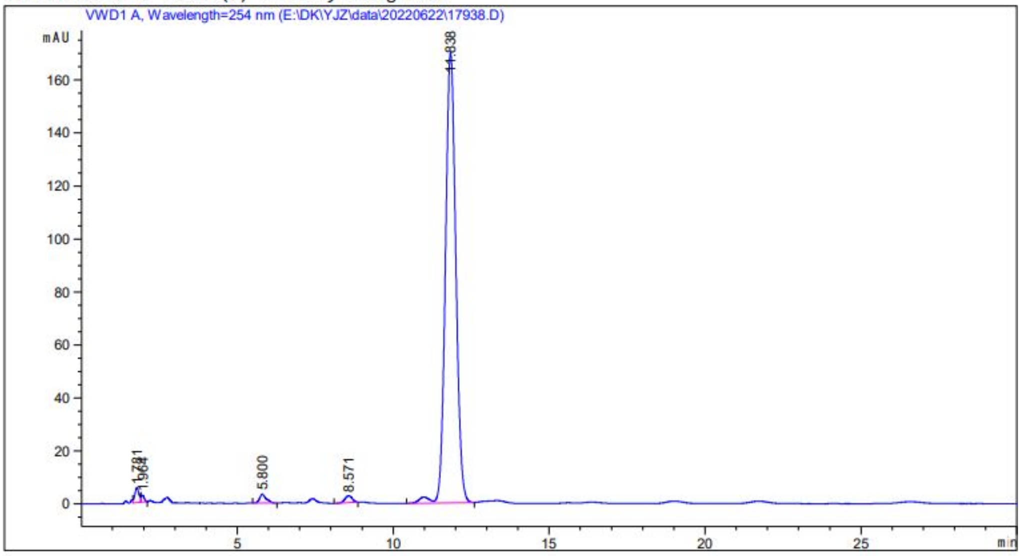

===

Area Percent Report

===

Sorted By : Signal
Multiplier : 1.0000
Dilution : 1.0000
Sample Amount: : 1.00000 [ng/ul] (not used in calc.)
Use Multiplier & Dilution Factor with ISTDs

Signal 1: VWD1 A, Wavelength=254 nm

Peak #	RetTime [min]	Type	Width [min]	Area [mAU*s]	Height [mAU]	Area %
1	1.781	VV	0.1370	53.07150	5.67924	1.2894
2	1.964	VB	0.0996	17.45209	2.58930	0.4240
3	5.800	BB	0.2157	51.23201	3.40125	1.2447
4	8.571	BB	0.2630	44.15936	2.64166	1.0729
5	11.838	VB R	0.3556	3950.08398	169.95958	95.9690

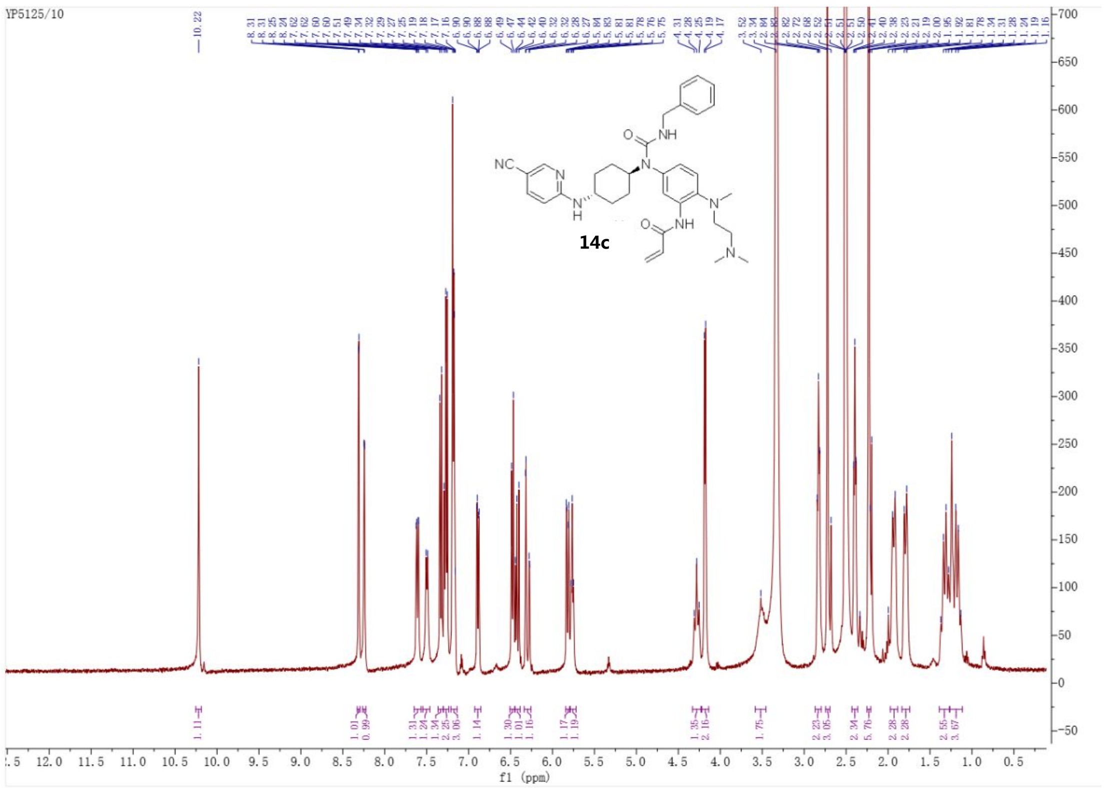

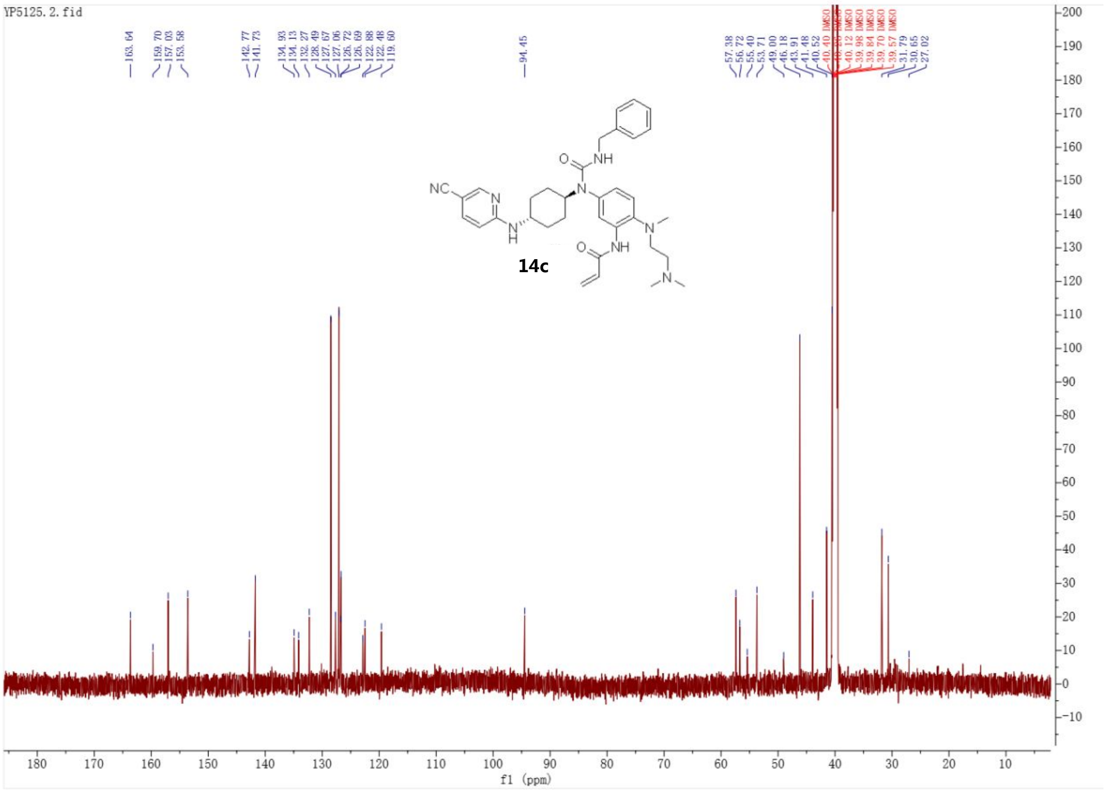

Hit	Formula	m/z	RDB	ppm	MS Rank	MSMS ppm	MSMS Rank	Found
1	C34H42N8O2	595.3503	18.0	0.8	1			NA/NA

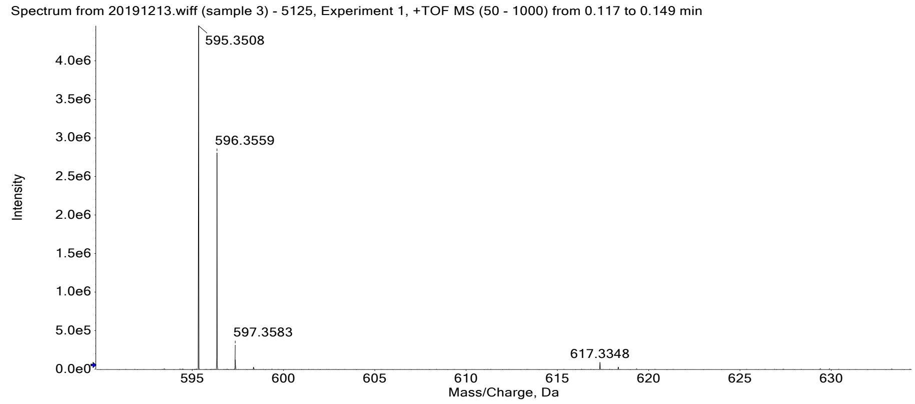


```
Data File E:\DK\YJZ\data\20191203\51252019-12-0317-31-27.D
Sample Name: 5125

    =====================================================================
    Acq. Operator   : 系统
    Sample Operator : 系统
    Acq. Instrument : 1260LC                        Location :   22
    Injection Date  : 03/12/2019 17:32:08
                                                  Inj Volume : 5.000 µl
    Acq. Method     : E:\DK\TL\方法\80C-20D-30min-1u.M
    Last changed    : 03/12/2019 17:25:55 by 系统
                      (modified after loading)
    Analysis Method : E:\DK\TL\方法\70C-30D-30min-1u.M
    Last changed    : 25/02/2022 21:41:46 by 系统
```

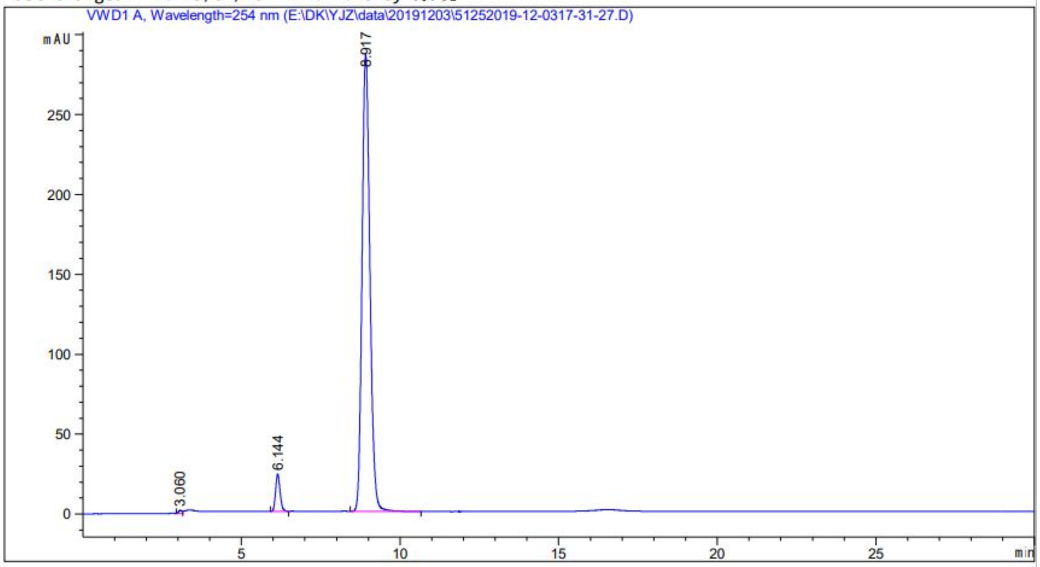


```
    =====================================================================
                             Area Percent Report
    =====================================================================

    Sorted By             :      Signal
    Multiplier            :      1.0000
    Dilution              :      1.0000
    Sample Amount:               :      20.00000  [ng/ul]   (not used in calc.)
    Use Multiplier & Dilution Factor with ISTDs


    Signal 1: VWD1 A, Wavelength=254 nm
```

Peak #	RetTime [min]	Type	Width [min]	Area [mAU*s]	Height [mAU]	Area %
1	3.060	BV	0.1070	14.98677	2.03270	0.2905
2	6.144	BB	0.1517	229.96640	23.38974	4.4571
3	8.917	BB	0.2657	4914.63867	285.74664	95.2525

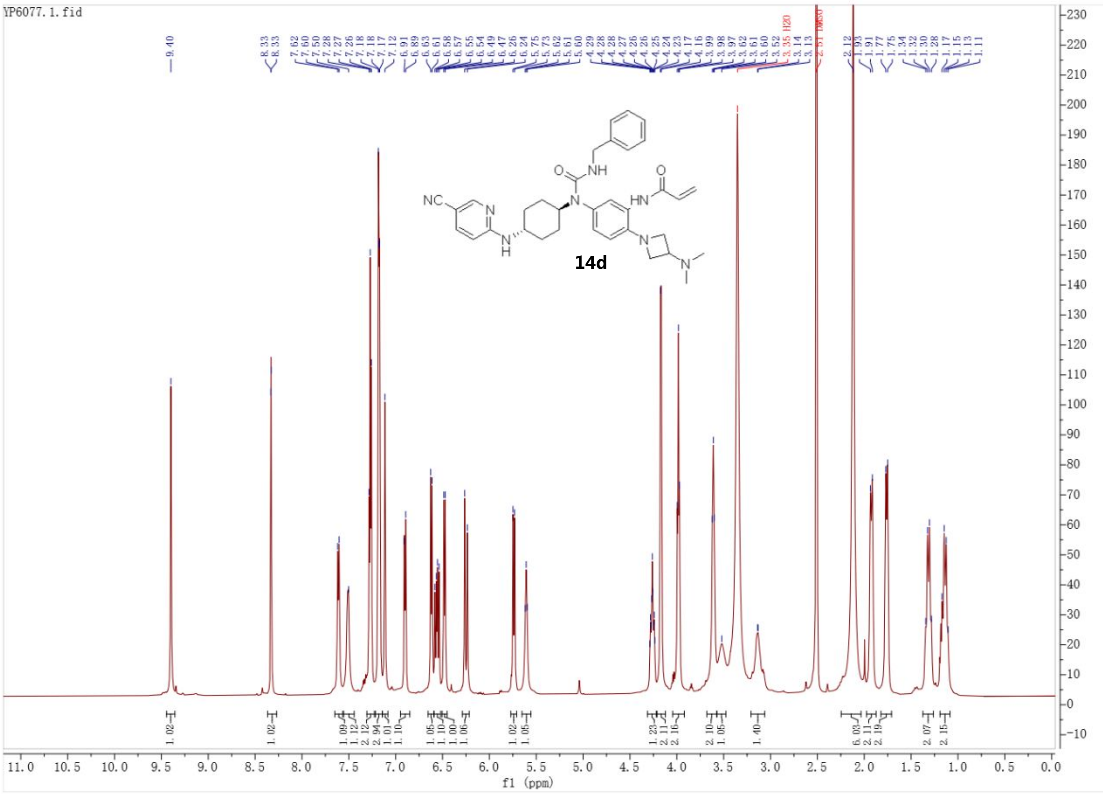

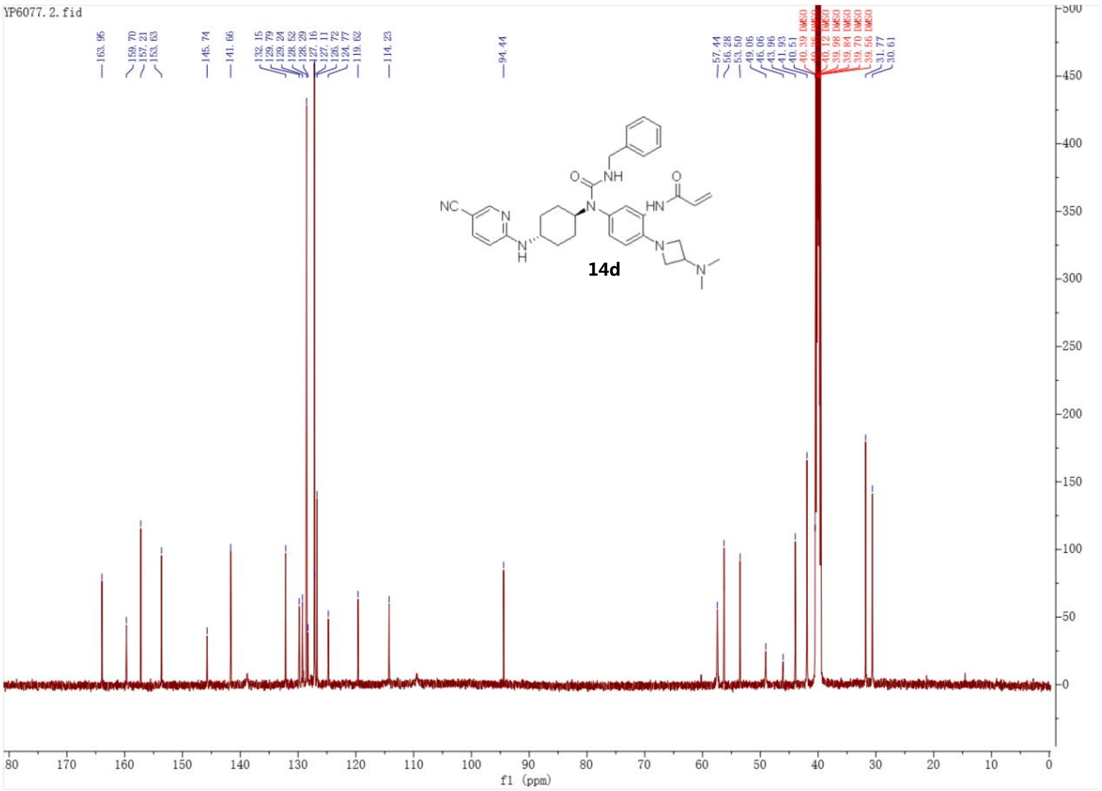

Hit	Formula	m/z	RDB	ppm	MS Rank	MSMS ppm	MSMS Rank	Found
1	C34H40N8O2	593.3347	19.0	-3.0	1			NA/NA

Spectrum from 20210406-WJW-ZFT.wiff2 (sample 46) - 6017, +TOF MS (100 - 1500) from 0.025 to 0.119 min

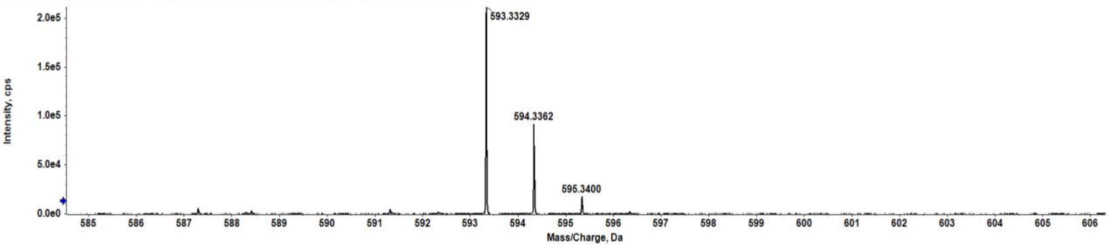

Data File E:\DK\YJZ\data\20200605\60772020-06-0519-43-03.D
Sample Name: 6077

===

Acq. Operator : 系统
Sample Operator : 系统
Acq. Instrument : 1260LC   Location : 31
Injection Date : 05/06/2020 19:43:44
Inj Volume : 5.000 µl
Acq. Method : E:\DK\YJZ\METHOD\70C_30D30min_254.0nM.M
Last changed : 05/12/2017 21:38:11 by 系统
Analysis Method : E:\DK\TL\方法\70C-30D-30min-1u.M
Last changed : 25/02/2022 21:41:46 by 系统

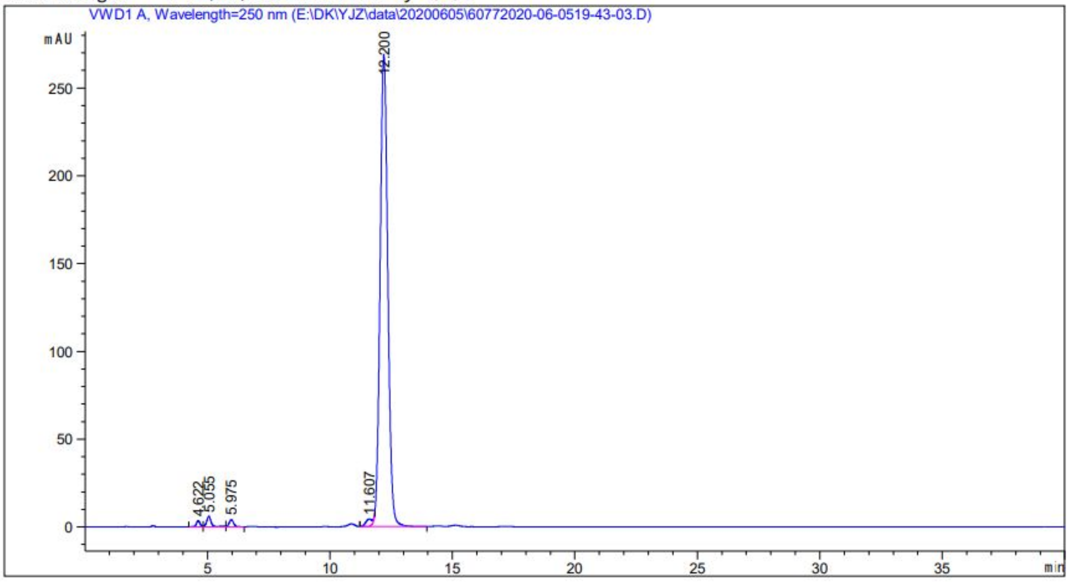

===

Area Percent Report

===

Sorted By : Signal
Multiplier : 1.0000
Dilution : 1.0000
Sample Amount: : 5.00000 [ng/ul] (not used in calc.)
Use Multiplier & Dilution Factor with ISTDs

Signal 1: VWD1 A, Wavelength=250 nm

Peak #	RetTime [min]	Type	Width [min]	Area [mAU*s]	Height [mAU]	Area %
1	4.622	BV	0.1605	37.24383	3.54570	0.6073
2	5.055	VV R	0.2008	81.01887	5.95385	1.3211
3	5.975	VB	0.1907	51.75875	4.14525	0.8440
4	11.607	BV E	0.2666	71.45096	4.11475	1.1651
5	12.200	VB R	0.3408	5891.35107	268.74084	96.0626

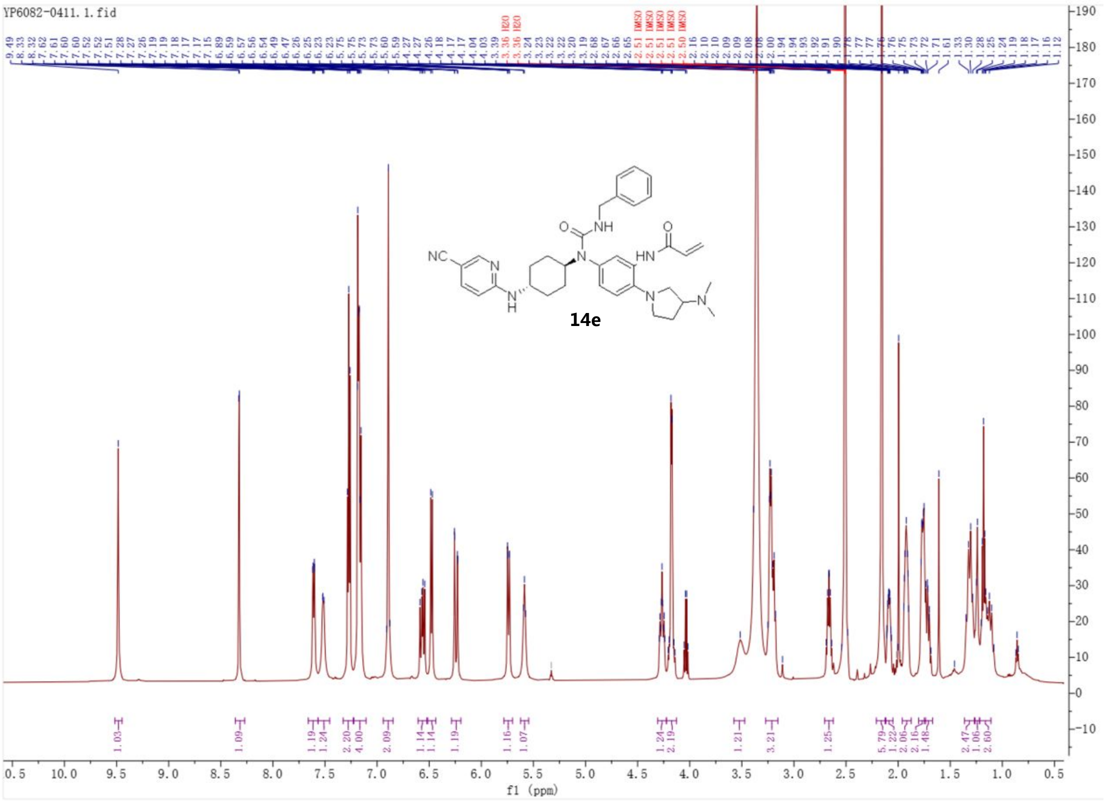

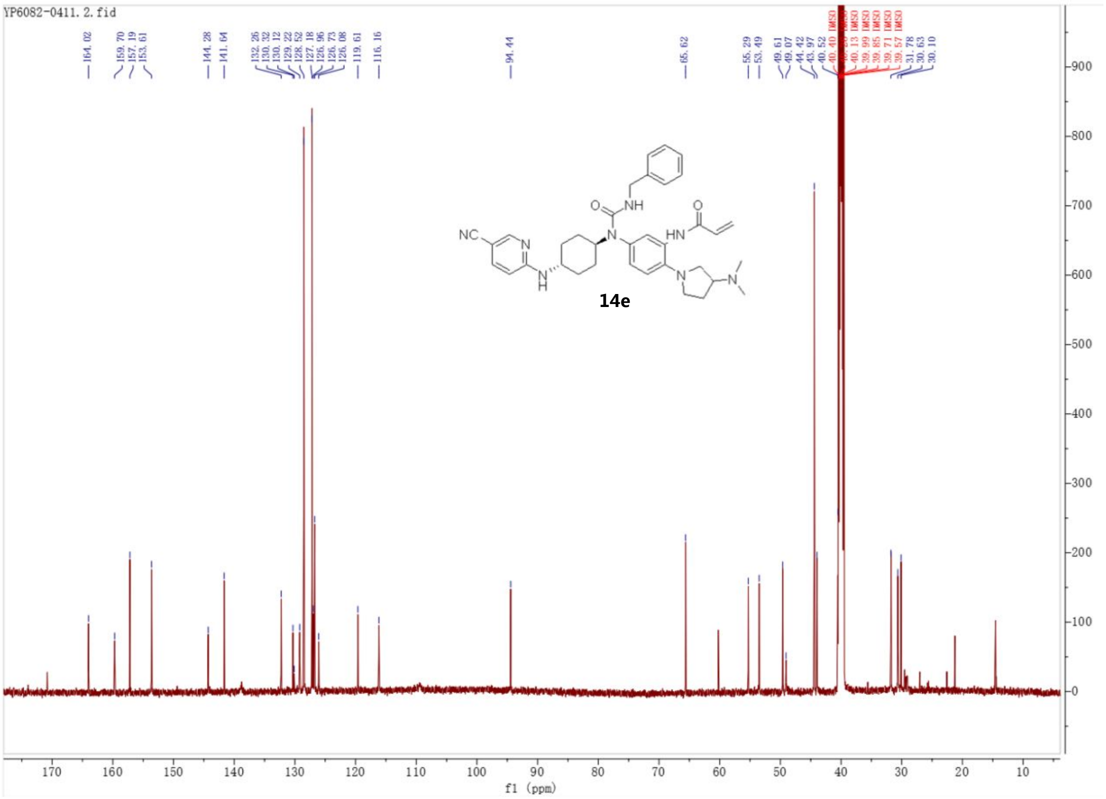

Hit	Formula	m/z	RDB	ppm	MS Rank	MSMS ppm	MSMS Rank	Found
1	C35H42N8O2	607.3503	19.0	-3.9	1			NA/NA

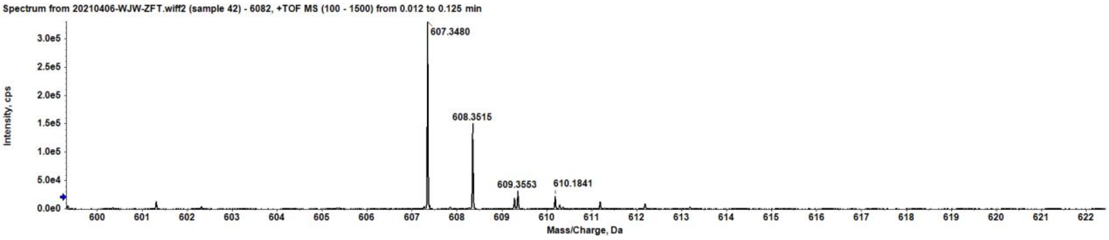

Data File E:\DK\YJZ\data\20200608\60822020-06-0814-35-55.D
Sample Name: 6082

===

Acq. Operator : 系统
Sample Operator : 系统
Acq. Instrument : 1260LC Location : 12
Injection Date : 08/06/2020 14:36:38
Inj Volume : 5.000 µl
Acq. Method : C:\CHEM32\1\METHODS\70C-30D-254NM.M
Last changed : 06/12/2019 14:32:59 by 系统
Analysis Method : E:\DK\TL\方法\70C-30D-30min-1u.M
Last changed : 25/02/2022 21:41:46 by 系统

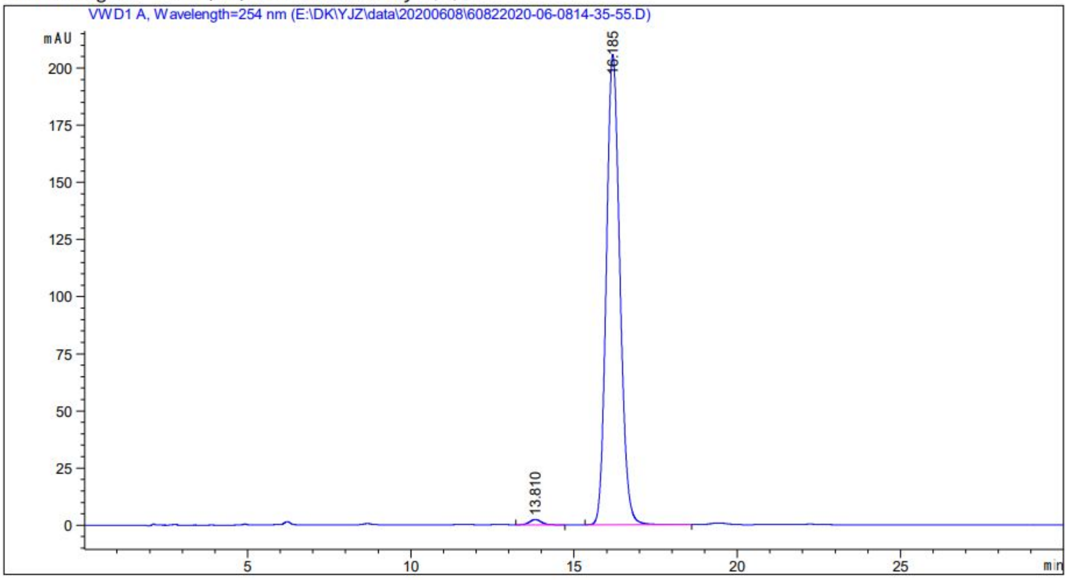

===
Area Percent Report
===

Sorted By : Signal
Multiplier : 1.0000
Dilution : 1.0000
Sample Amount: : 5.00000 [ng/ul] (not used in calc.)
Use Multiplier & Dilution Factor with ISTDs

Signal 1: VWD1 A, Wavelength=254 nm

Peak #	RetTime [min]	Type	Width [min]	Area [mAU*s]	Height [mAU]	Area %
1	13.810	BB	0.3710	55.84170	2.29372	0.9343
2	16.185	BB	0.4468	5921.19873	205.71477	99.0657

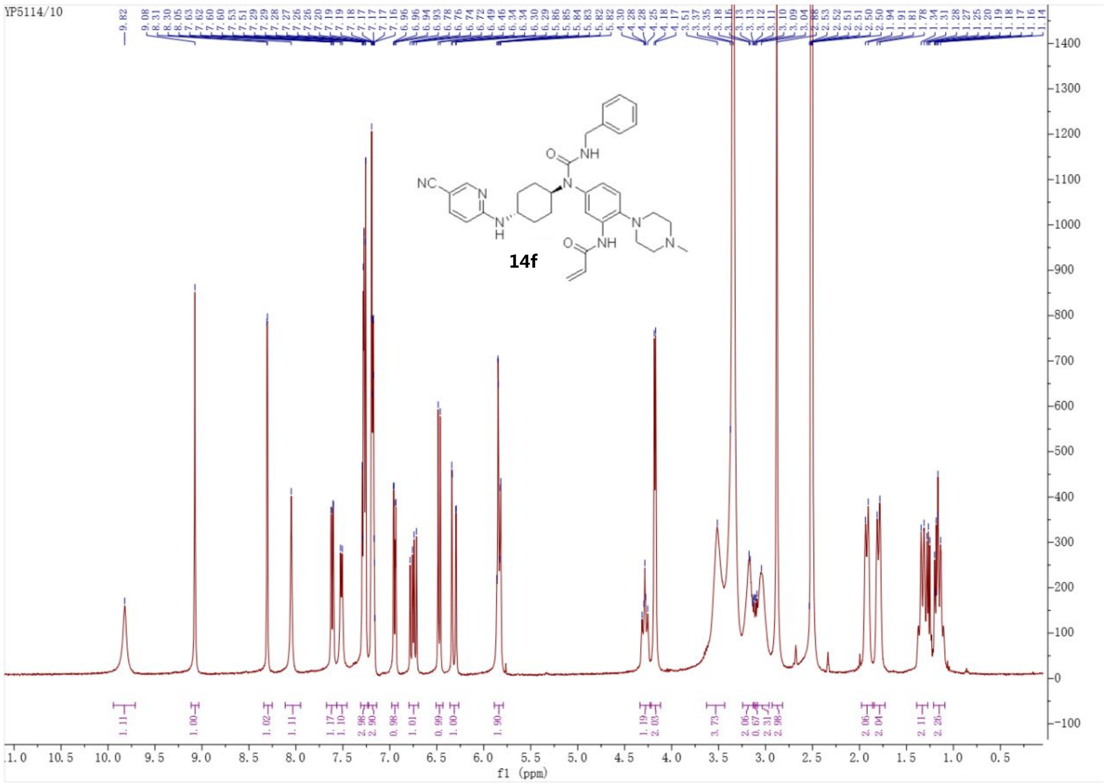

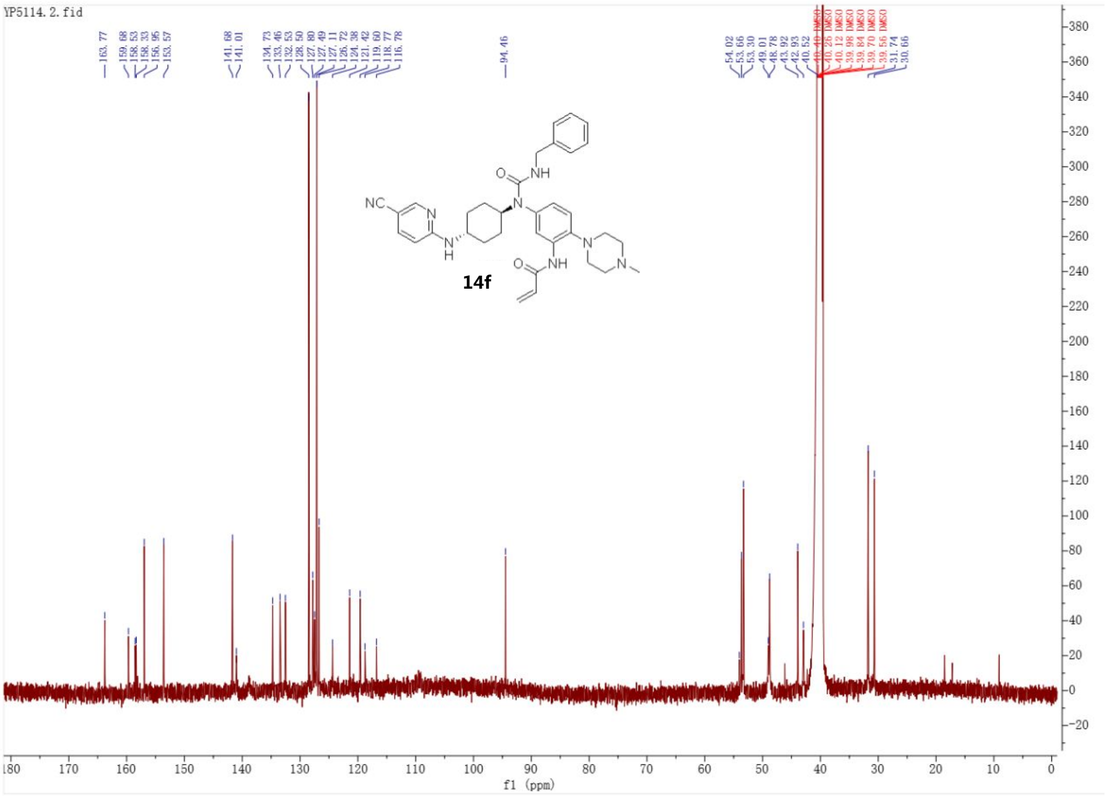

Hit	Formula	m/z	RDB	ppm	MS Rank	MSMS ppm	MSMS Rank	Found
1	$C_{34}H_{40}N_8O_2$	593.3347	19.0	3.0	1			NA/NA

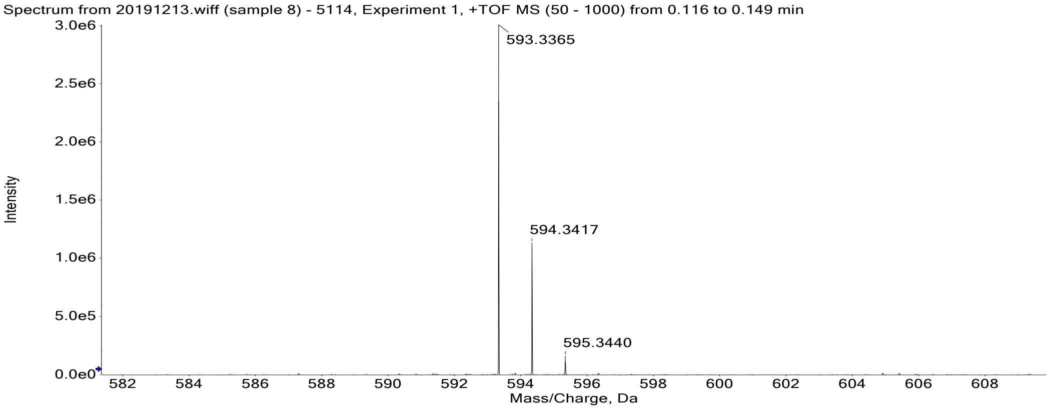

Data File E:\DK\YJZ\data\20191202\51142019-12-0214-06-33.D
Sample Name: 5114

===
Acq. Operator : 系统
Sample Operator : 系统
Acq. Instrument : 1260LC Location : 55
Injection Date : 02/12/2019 14:07:15
Inj Volume : 5.000 µl
Acq. Method : E:\DK\TL\方法\80C-20D-30min-1u.M
Last changed : 02/12/2019 14:26:21 by 系统
(modified after loading)
Analysis Method : E:\DK\TL\方法\70C-30D-30min-1u.M
Last changed : 25/02/2022 21:41:46 by 系统

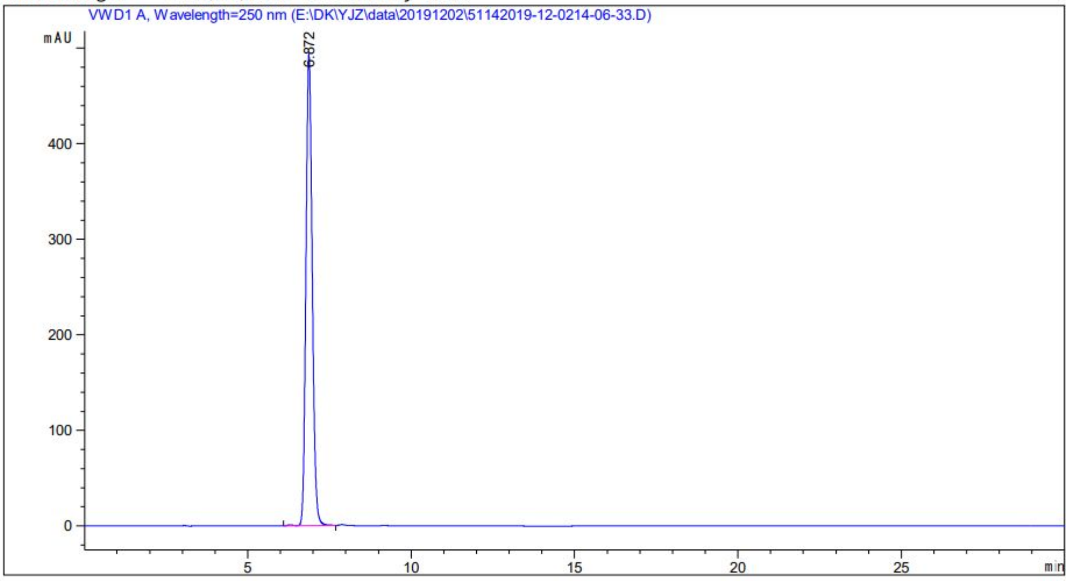

===
Area Percent Report
===

Sorted By : Signal
Multiplier : 1.0000
Dilution : 1.0000
Sample Amount: : 5.00000 [ng/ul] (not used in calc.)
Use Multiplier & Dilution Factor with ISTDs

Signal 1: VWD1 A, Wavelength=250 nm

Peak #	RetTime [min]	Type	Width [min]	Area [mAU*s]	Height [mAU]	Area %
1	6.872	VB R	0.2085	6615.71240	493.20804	100.0000

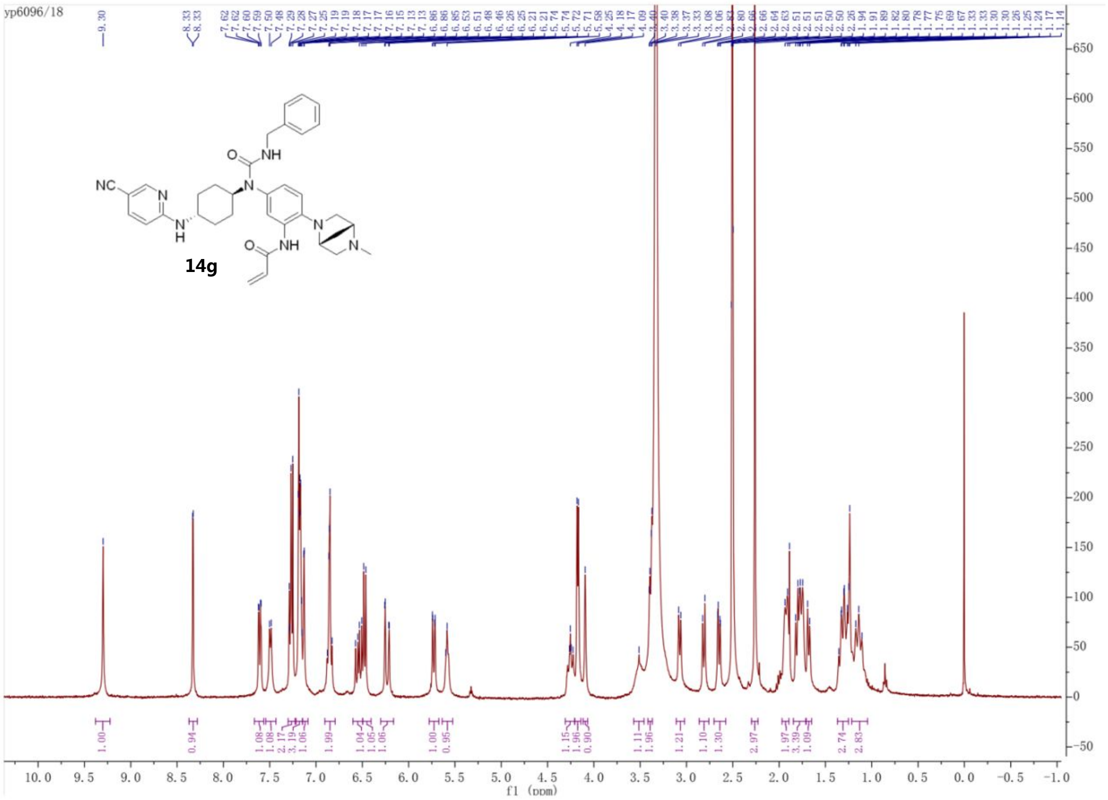

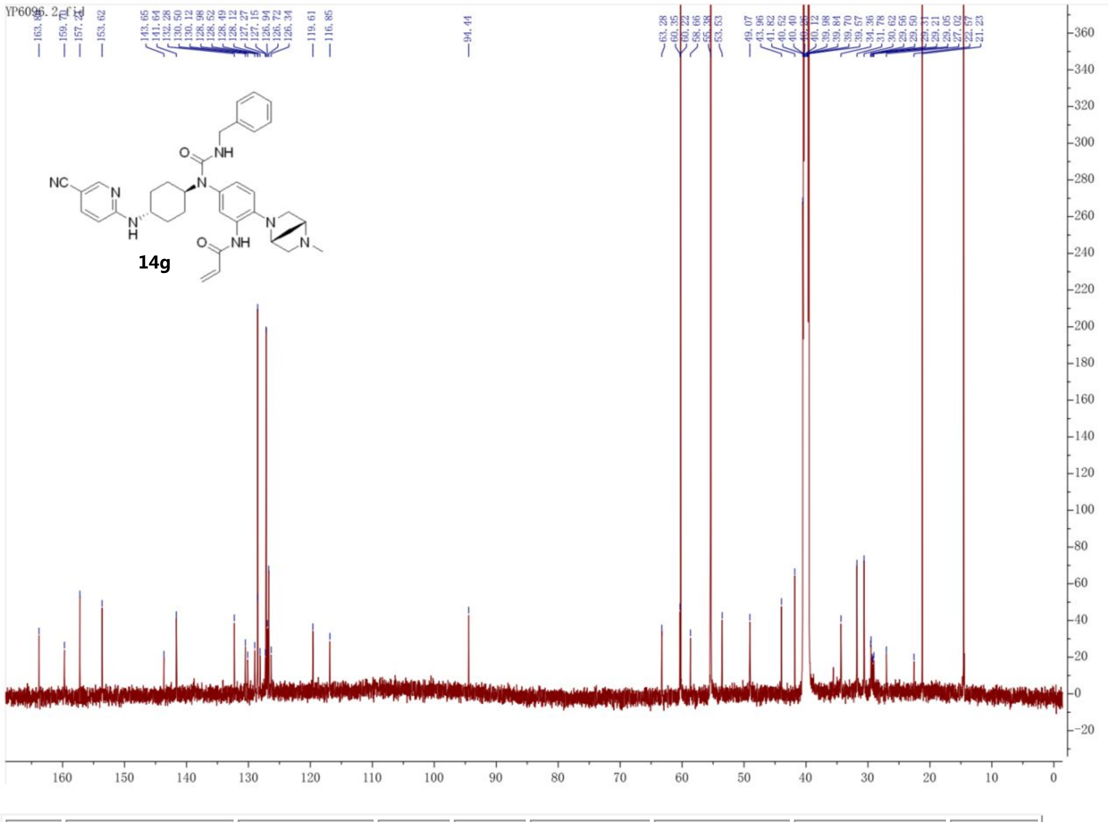

Hit	Formula	m/z	RDB	ppm	MS Rank	MSMS ppm	MSMS Rank	Found
1	C35H40N8O2	605.3347	20.0	-3.0	1			NA/NA

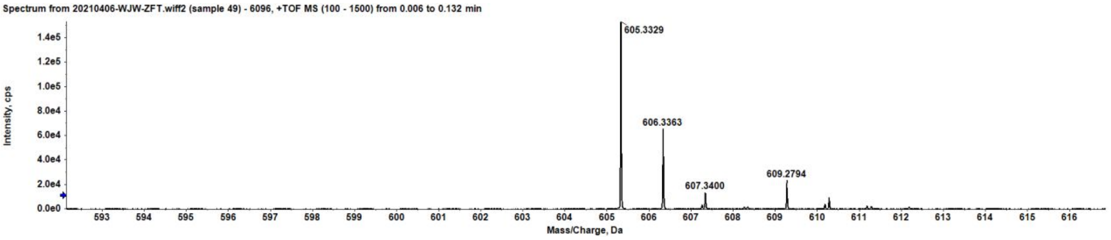

Data File E:\DK\YJZ\data\20200611\60962020-06-1216-22-34.D
Sample Name: 6096

===

Acq. Operator : 系统
Sample Operator : 系统
Acq. Instrument : 1260LC Location : 2
Injection Date : 12/06/2020 16:23:14
Inj Volume : 10.000 µl
Acq. Method : E:\DK\YJZ\METHOD\70C_30D30min_254.0nM.M
Last changed : 12/06/2020 16:22:21 by 系统
(modified after loading)
Analysis Method : E:\DK\TL\方法\70C-30D-30min-1u.M
Last changed : 25/02/2022 21:41:46 by 系统

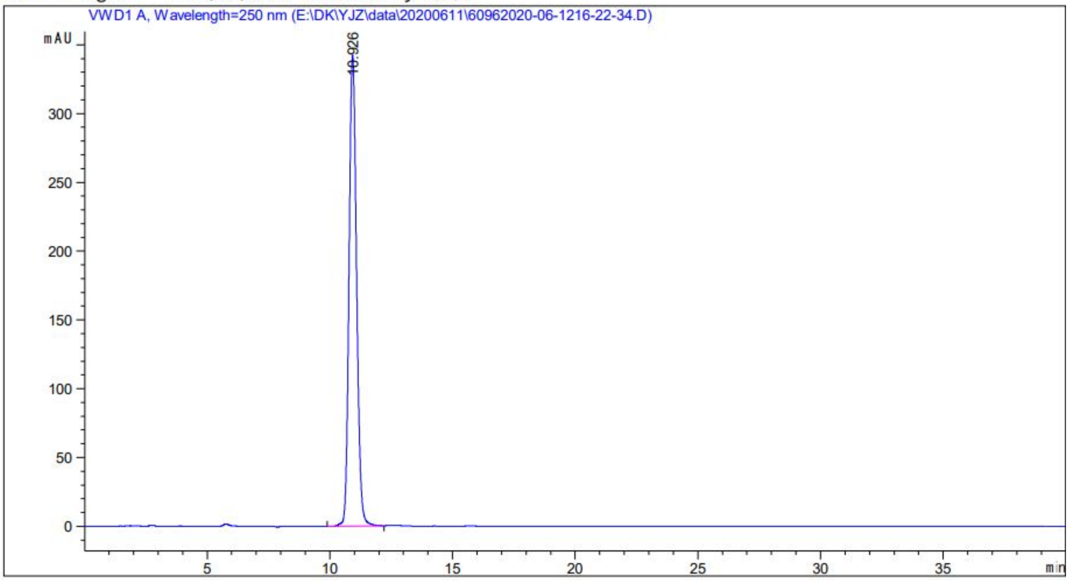

===
Area Percent Report
===

Sorted By : Signal
Multiplier : 1.0000
Dilution : 1.0000
Sample Amount: : 10.00000 [ng/ul] (not used in calc.)
Use Multiplier & Dilution Factor with ISTDs

Signal 1: VWD1 A, Wavelength=250 nm

Peak #	RetTime [min]	Type	Width [min]	Area [mAU*s]	Height [mAU]	Area %
1	10.926	BB	0.3309	7306.54150	342.49875	100.0000

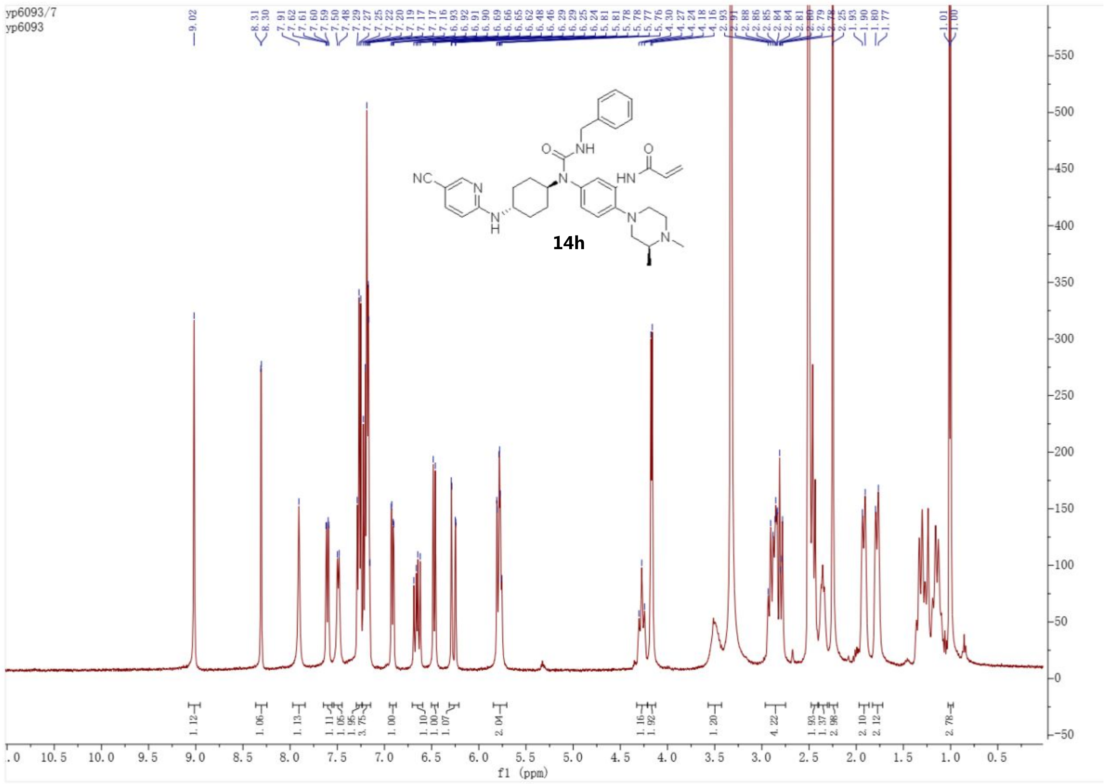

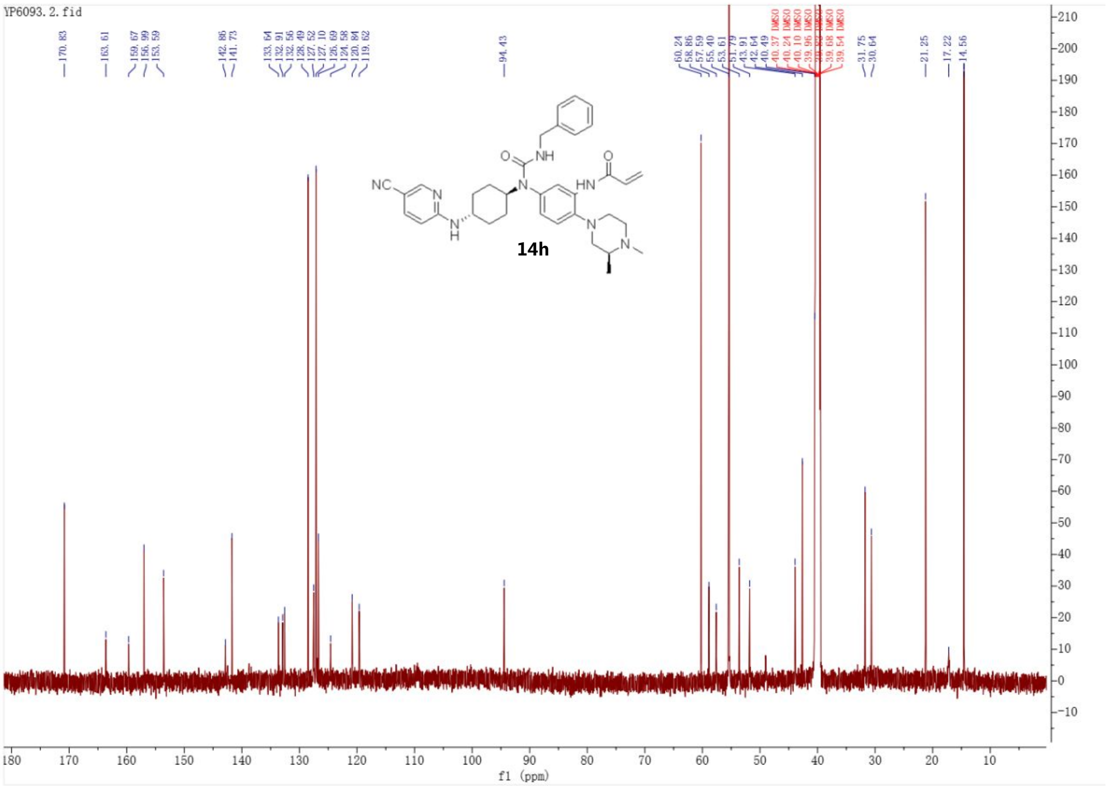

Hit	Formula	m/z	RDB	ppm	MS Rank	MSMS ppm	MSMS Rank	Found
1	C35H42N8O2	607.3503	19.0	-3.0	1			NA/NA

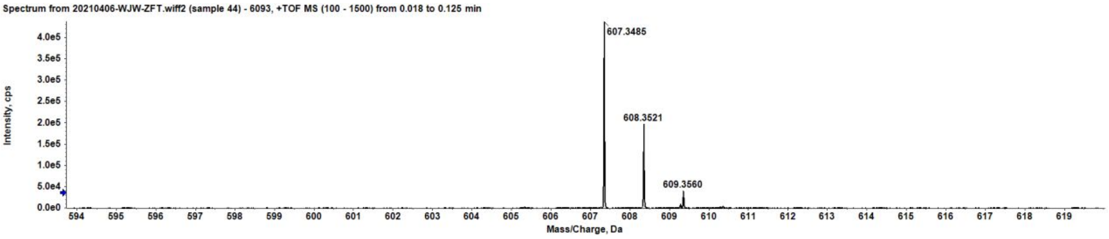


```
Data File E:\DK\YJZ\data\20201105\60932020-11-0521-50-47.D
Sample Name: 6093

    =====================================================================
    Acq. Operator   : 系统
    Sample Operator : 系统
    Acq. Instrument : 1260LC                        Location :   65
    Injection Date  : 05/11/2020 21:51:27
                                                  Inj Volume : 5.000 µl
    Acq. Method     : E:\DK\CY\方法\80p_30min_254nM.M
    Last changed    : 08/02/2017 14:13:15 by 系统
    Analysis Method : E:\DK\TL\方法\70C-30D-30min-1u.M
    Last changed    : 25/02/2022 21:41:46 by 系统
```

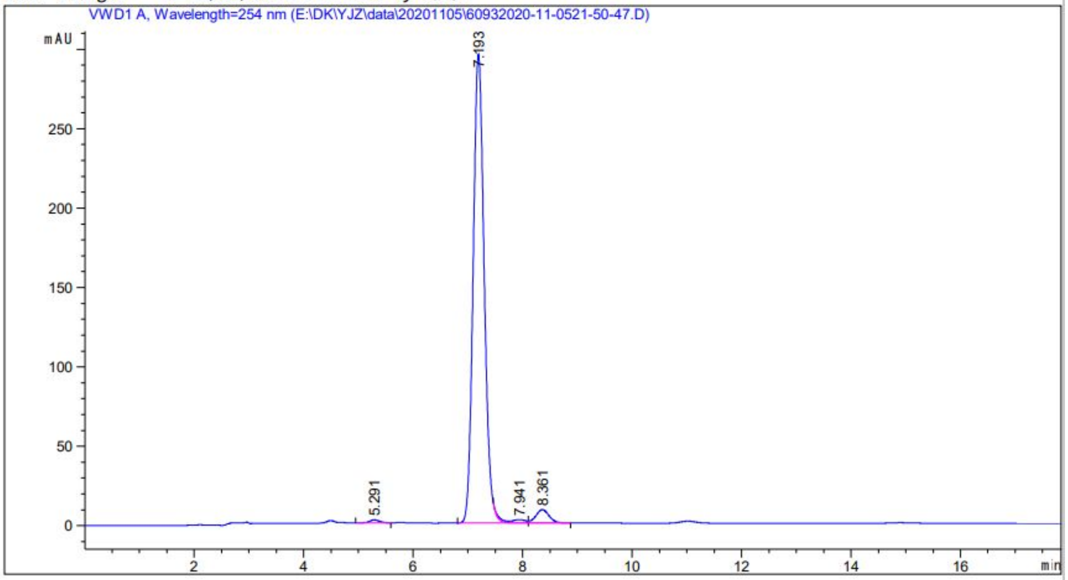


```
    =====================================================================
                              Area Percent Report
    =====================================================================

    Sorted By             :      Signal
    Multiplier            :      1.0000
    Dilution              :      1.0000
    Sample Amount:               :      5.00000  [ng/ul]   (not used in calc.)
    Use Multiplier & Dilution Factor with ISTDs


    Signal 1: VWD1 A, Wavelength=254 nm

    Peak RetTime Type  Width     Area      Height     Area
      #   [min]        [min]   [mAU*s]     [mAU]       %
    ----|-------|----|-------|----------|----------|--------|
       1   5.291 BB    0.1879   24.01143    1.91970   0.5539
       2   7.193 BV R  0.2160 4122.63916  295.11609  95.1043
       3   7.941 VV E  0.3181   48.43318    2.13933   1.1173
       4   8.361 VB E  0.2548  139.77904    8.41285   3.2245
```

Hit	Formula	m/z	RDB	ppm	MS Rank	MSMS ppm	MSMS Rank	Found
1	C35H41BrN8O2	685.2609	19.0	-2.1	1			NA/NA

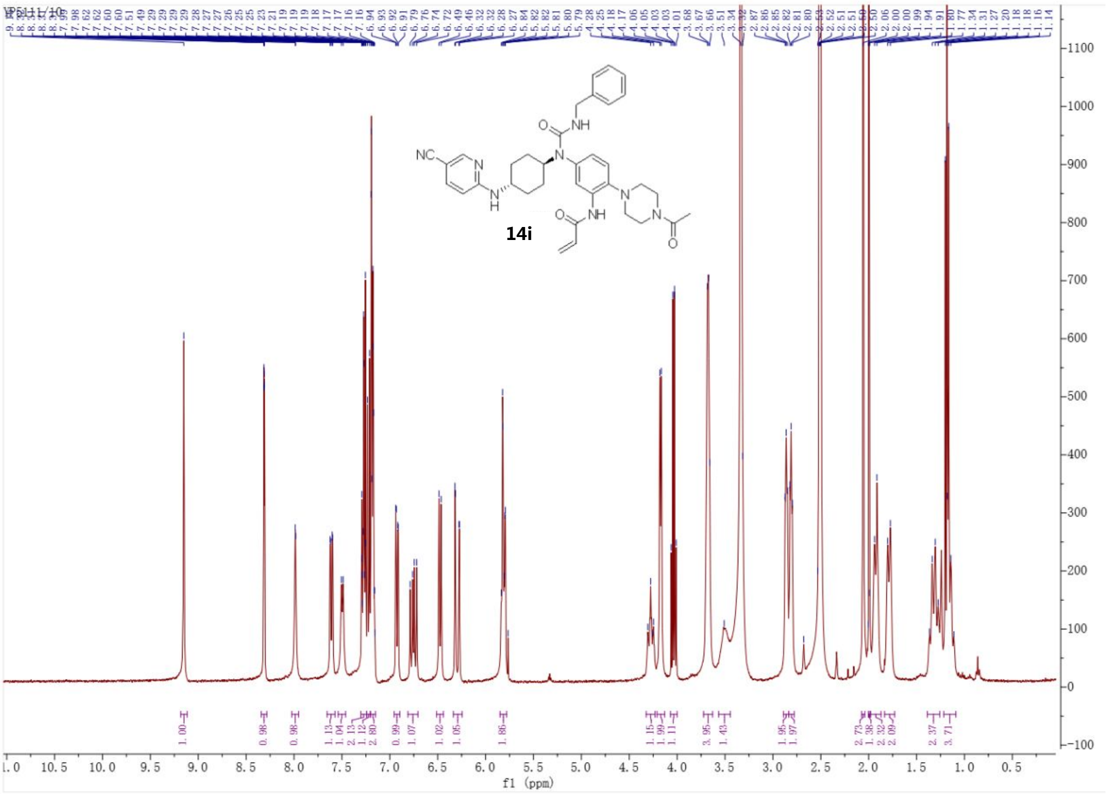

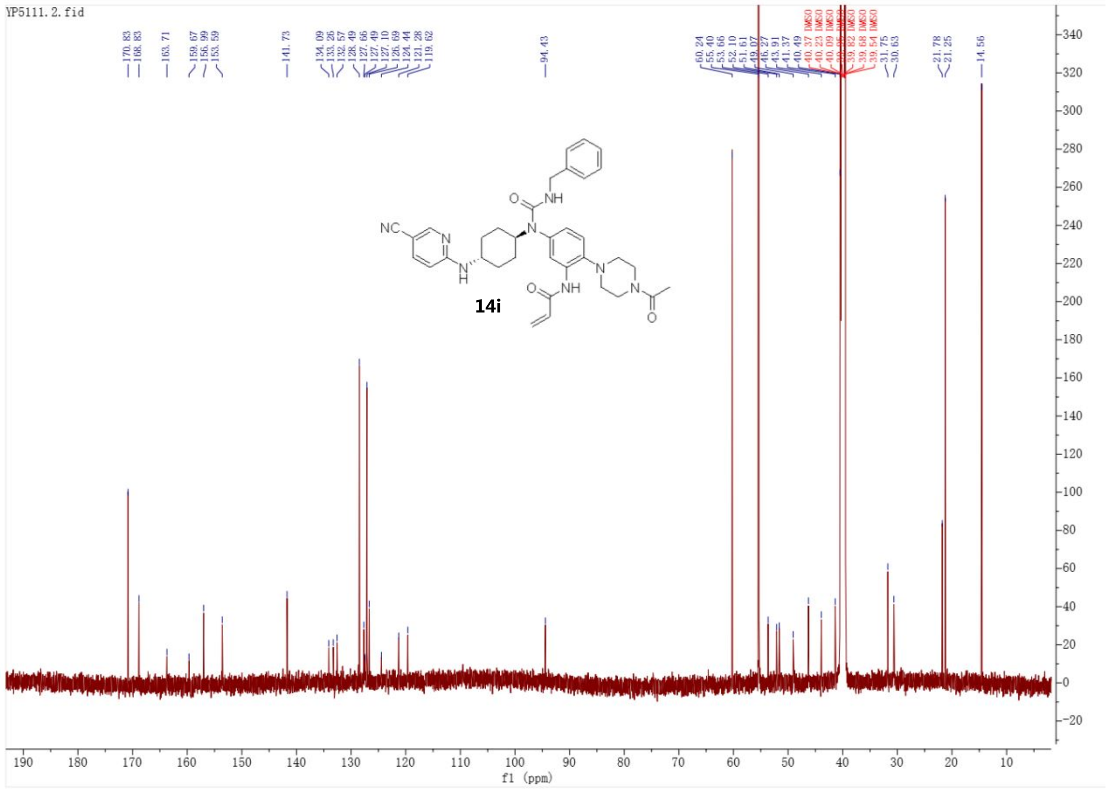

Hit	Formula	m/z	RDB	ppm	MS Rank	MSMS ppm	MSMS Rank	Found
1	C35H40N8O3	621.3296	20.0	-0.2	1			NA/NA

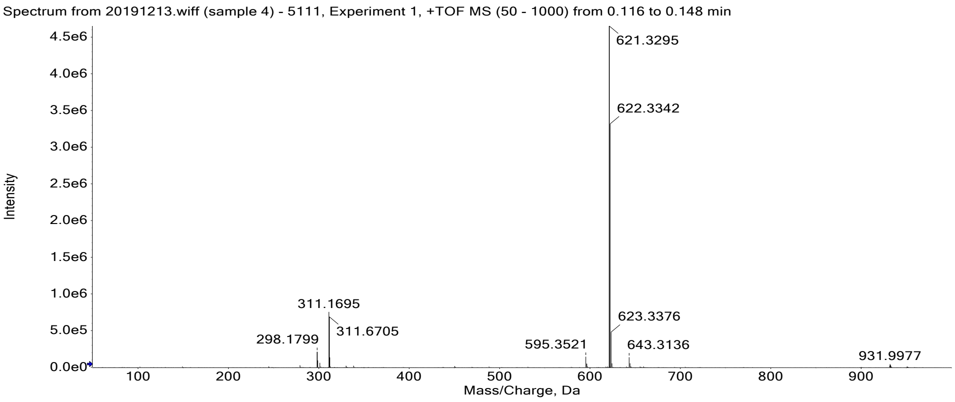

Data File E:\DK\YJZ\data\20191202\51112019-12-0217-42-26.D
Sample Name: 5111

===

Acq. Operator : 系统
Sample Operator : 系统
Acq. Instrument : 1260LC Location : 5
Injection Date : 02/12/2019 17:43:10
Inj Volume : 5.000 µl
Acq. Method : E:\DK\TL\方法\70C-30D-30min-1u.M
Last changed : 20/11/2019 13:02:51 by 系统
Analysis Method : E:\DK\TL\方法\70C-30D-30min-1u.M
Last changed : 25/02/2022 21:41:46 by 系统

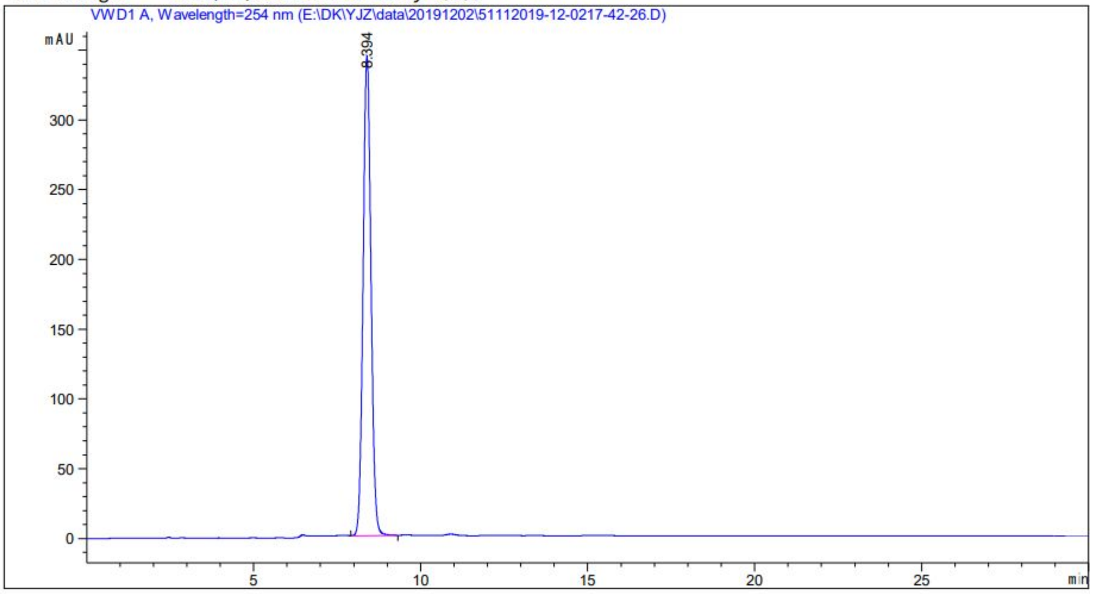

===

Area Percent Report

===

Sorted By : Signal
Multiplier : 1.0000
Dilution : 1.0000
Sample Amount: : 5.00000 [ng/ul] (not used in calc.)
Use Multiplier & Dilution Factor with ISTDs

Signal 1: VWD1 A, Wavelength=254 nm

Peak #	RetTime [min]	Type	Width [min]	Area [mAU*s]	Height [mAU]	Area %
1	8.394	BB	0.2546	5646.87939	343.87299	100.0000

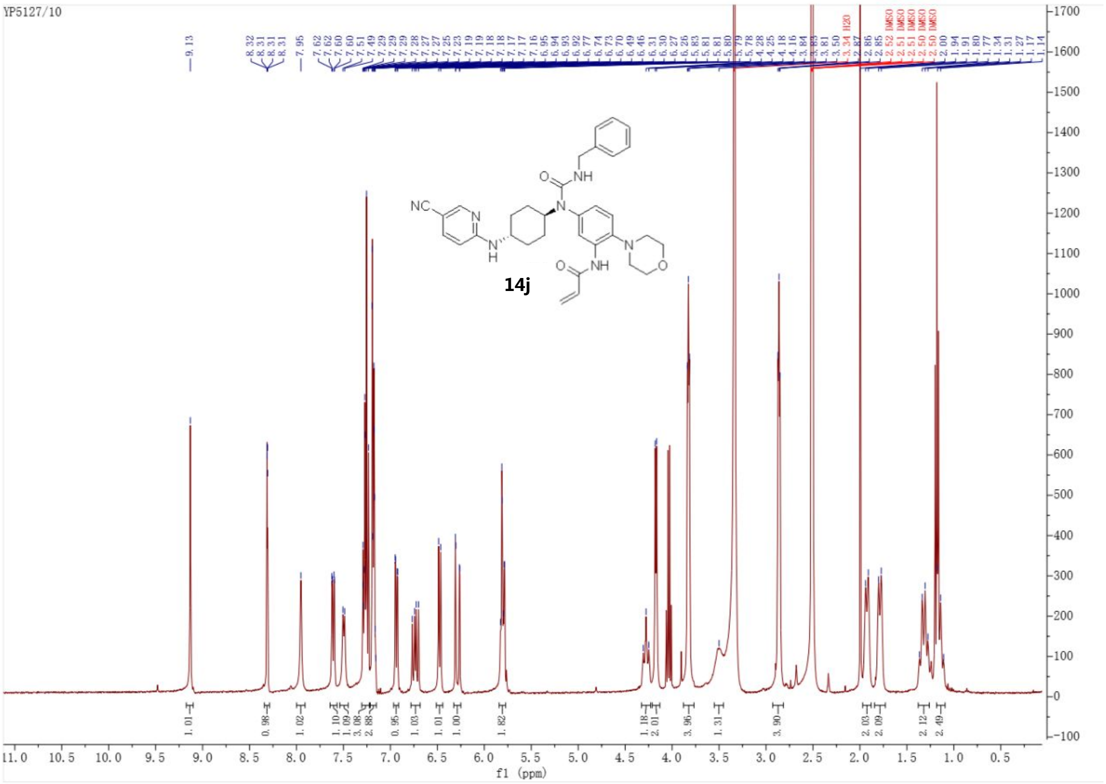
YP5127/10
14j
f1 (ppm)

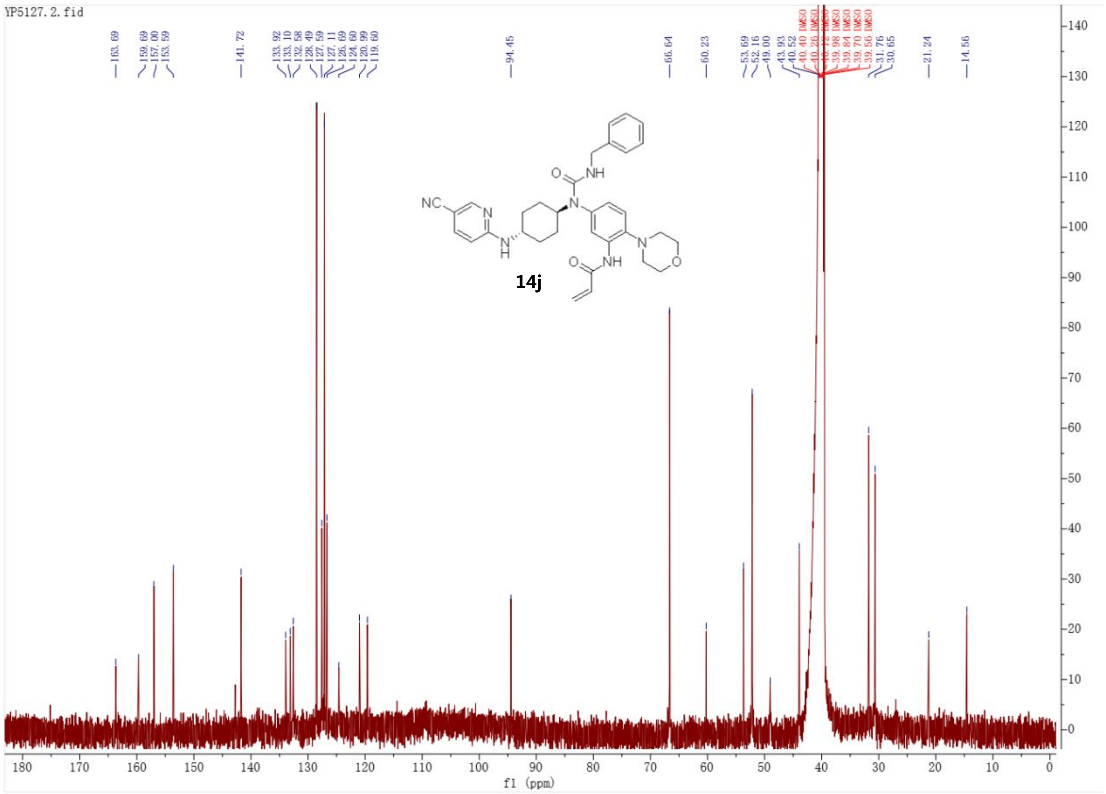
YP5127.2.fid
14j
f1 (ppm)

Hit	Formula	m/z	RDB	ppm	MS Rank	MSMS ppm	MSMS Rank	Found
1	C33H37N7O3	580.3031	19.0	-1.1	1			NA/NA

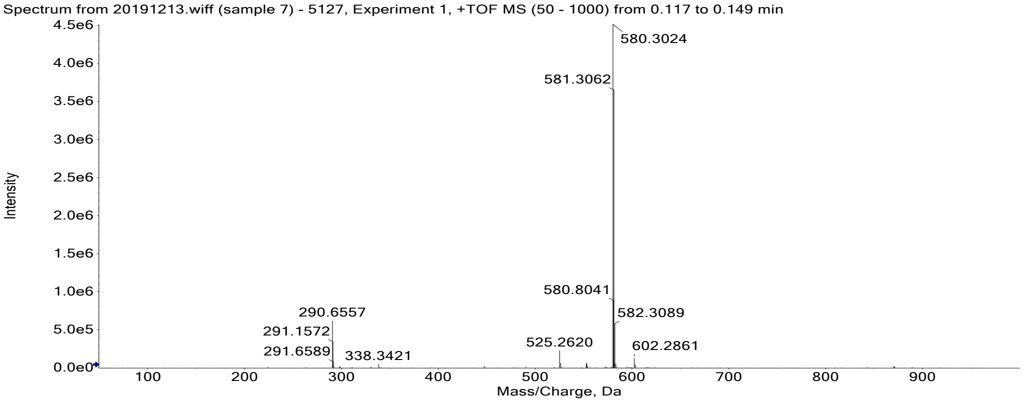

Data File E:\DK\YJZ\data\20191202\51272019-12-0218-24-04.D
Sample Name: 5127

===
Acq. Operator : 系统
Sample Operator : 系统
Acq. Instrument : 1260LC Location : 6
Injection Date : 02/12/2019 18:24:46
Inj Volume : 5.000 µl
Acq. Method : E:\DK\TL\方法\70C-30D-30min-1u.M
Last changed : 20/11/2019 13:02:51 by 系统
Analysis Method : C:\CHEM32\1\METHODS\80C-20D-30MIN-10UL.M
Last changed : 03/06/2022 15:26:09 by 系统

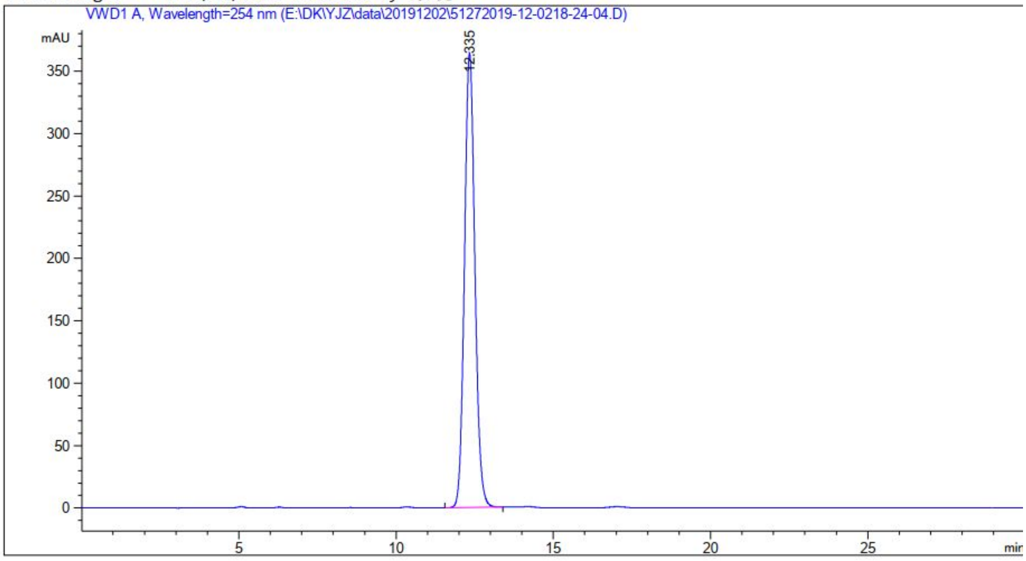

===
Area Percent Report
===

Sorted By : Signal
Multiplier : 1.0000
Dilution : 1.0000
Sample Amount: : 5.00000 [ng/ul] (not used in calc.)
Use Multiplier & Dilution Factor with ISTDs

Signal 1: VWD1 A, Wavelength=254 nm

Peak #	RetTime [min]	Type	Width [min]	Area [mAU*s]	Height [mAU]	Area %
1	12.335	BB	0.3567	8391.42578	364.36163	100.0000

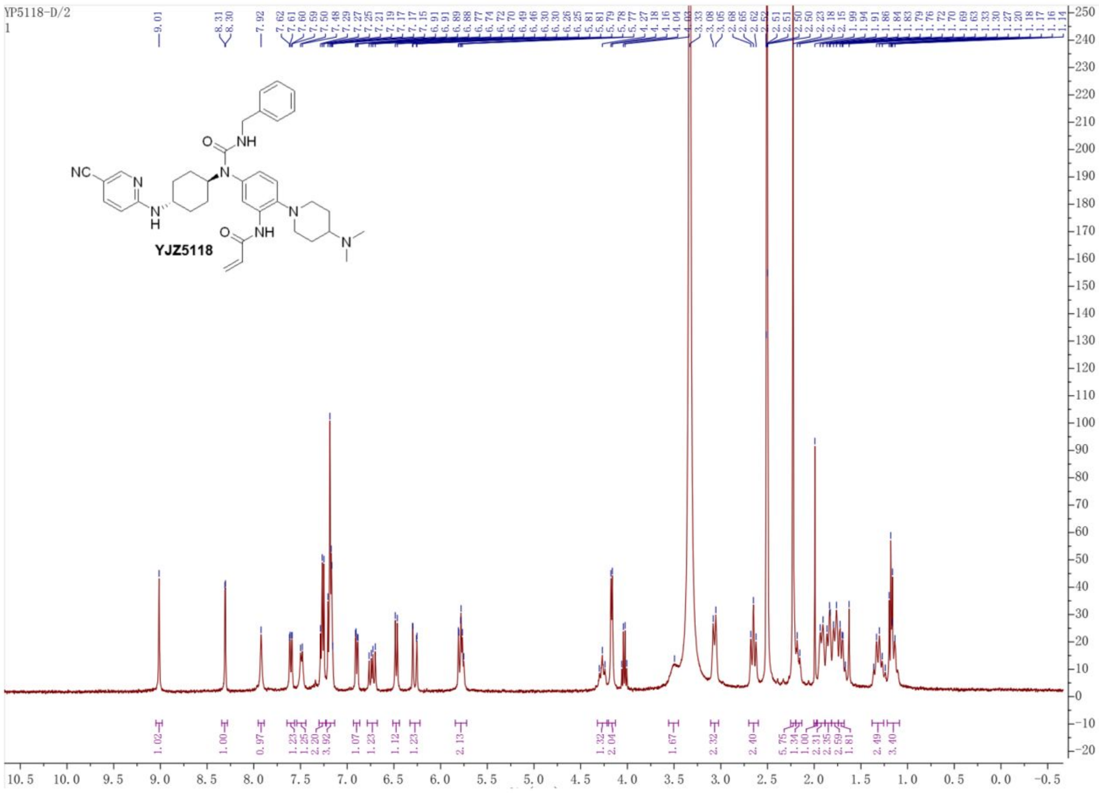

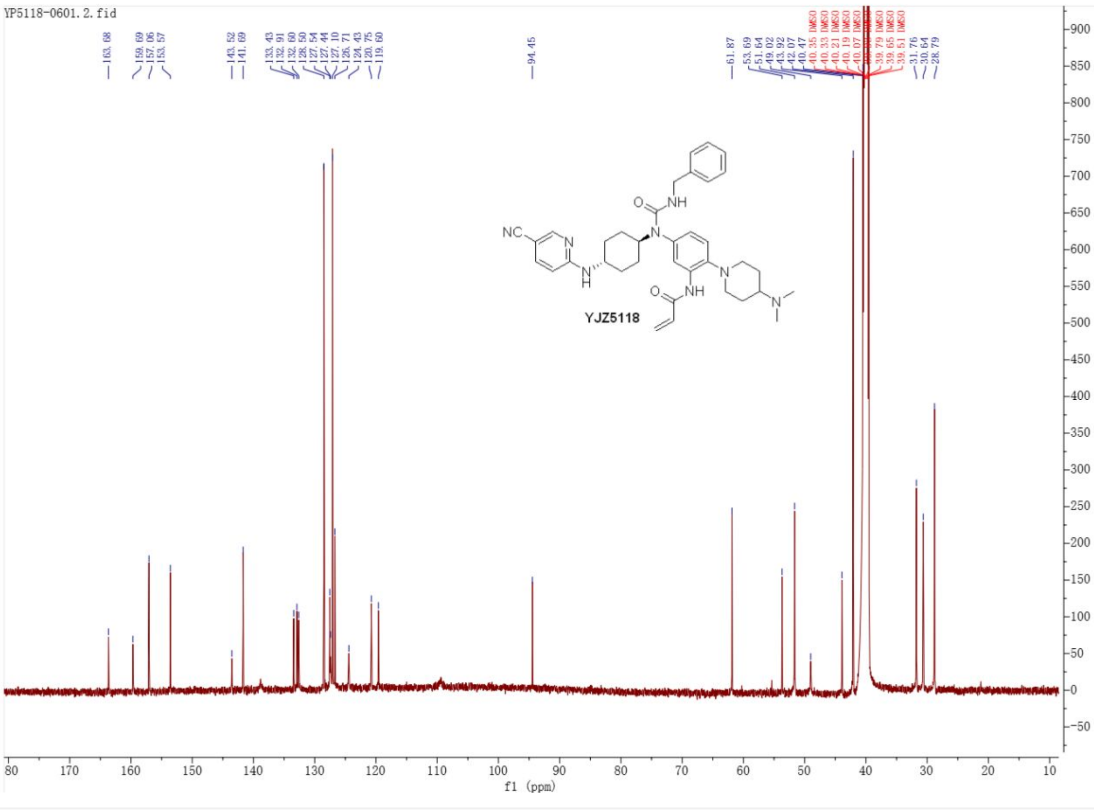

Hit	Formula	m/z	RDB	ppm	MS Rank	MSMS ppm	MSMS Rank	Found
1	C36H44N8O2	621.3660	19.0	1.0	1			NA/NA

Spectrum from 20191213.wiff (sample 6) - 5118, Experiment 1, +TOF MS (50 - 1000) from 0.116 to 0.149 min

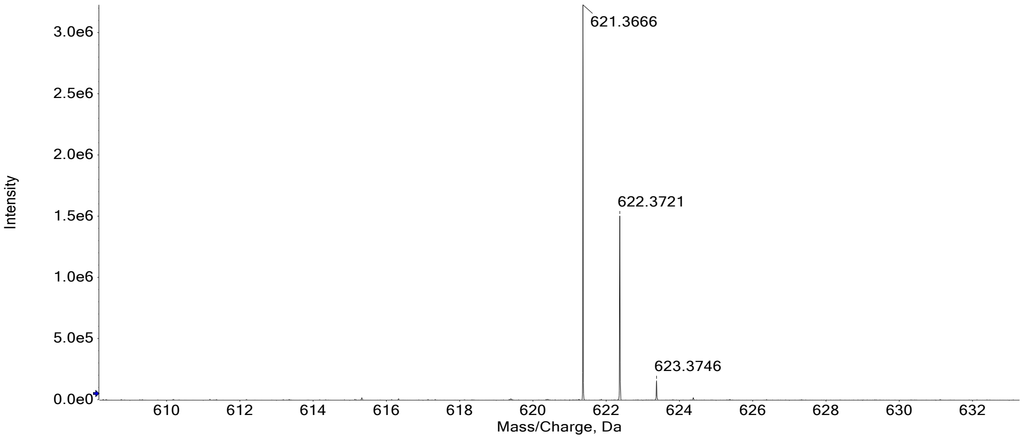


```
Data File E:\DK\YJZ\data\20201030\51182020-10-3121-59-59.D
Sample Name: 5118

    =====================================================================
    Acq. Operator   : 系统
    Sample Operator : 系统
    Acq. Instrument : 1260LC                         Location :   1
    Injection Date  : 31/10/2020 22:00:39
                                                   Inj Volume : 5.000 µl
    Acq. Method     : E:\DK\TL\方法\80C-20D-55MIN.M
    Last changed    : 07/03/2019 14:11:05 by 系统
    Analysis Method : C:\CHEM32\1\METHODS\80C-20D-30MIN-10UL.M
    Last changed    : 03/06/2022 15:26:09 by 系统
```

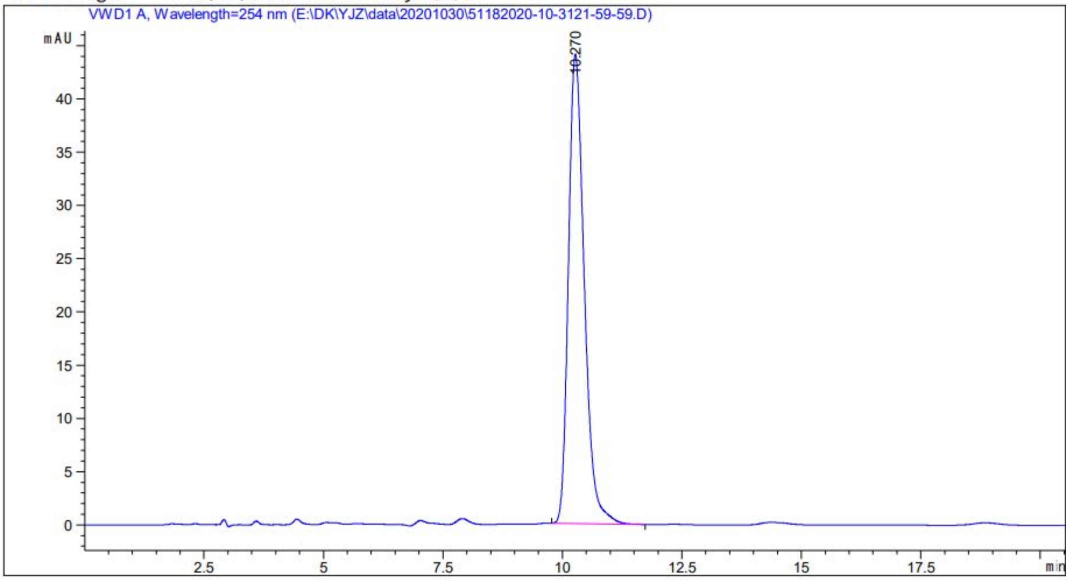


```
    =====================================================================
                            Area Percent Report
    =====================================================================

    Sorted By             :      Signal
    Multiplier            :      1.0000
    Dilution              :      1.0000
    Sample Amount:               :      5.00000  [ng/ul]   (not used in calc.)
    Use Multiplier & Dilution Factor with ISTDs


    Signal 1: VWD1 A, Wavelength=254 nm

    Peak RetTime Type  Width     Area      Height     Area
      #   [min]        [min]   [mAU*s]     [mAU]       %
    ----|-------|----|-------|----------|----------|--------|
       1  10.270 BB    0.3539 1011.60443   44.05629 100.0000
```

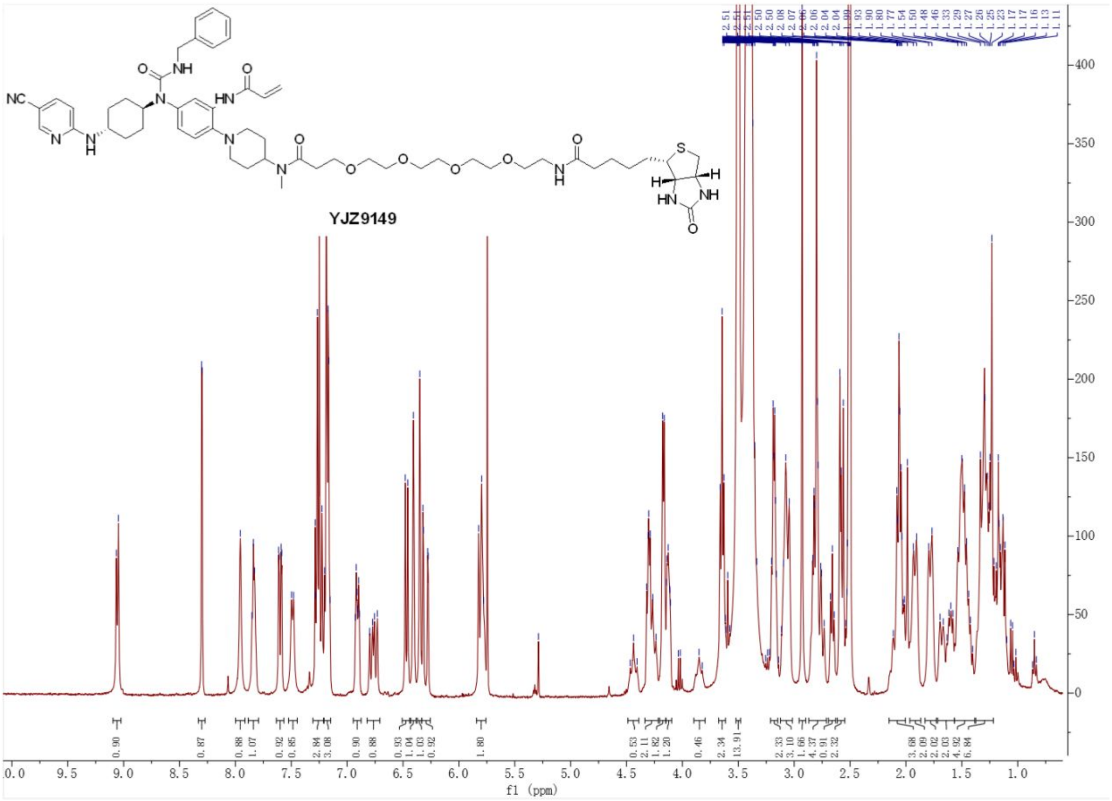
YJZ9149
f1 (ppm)

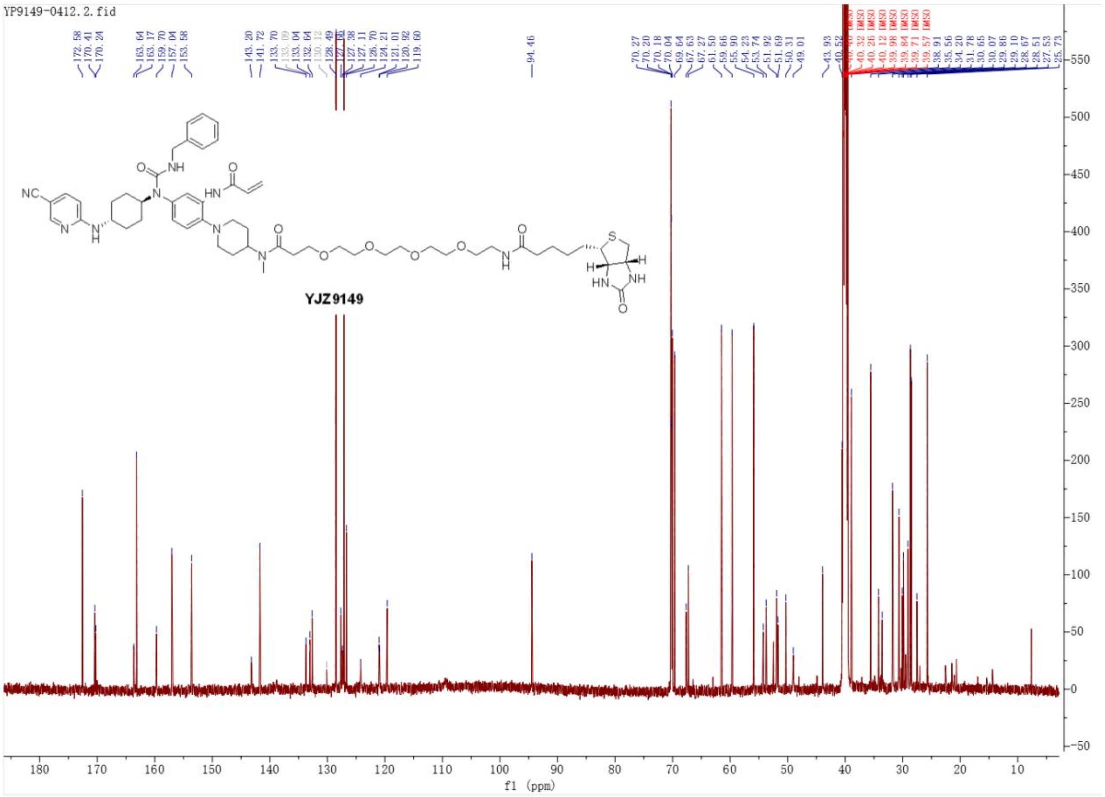
YP9149-0412.2.fid
YJZ9149
f1 (ppm)

Data File E:\DK\YJZ\data\20210715\YJZ1901DEF_LC 2021-07-16 22-36-17\9149.D
Sample Name: 9149

===

Acq. Operator : 系统 Seq. Line : 1
Acq. Instrument : 1260LC Location : 2
Injection Date : 16/07/2021 22:37:05 Inj : 1
Inj Volume : 5.000 µl
Sequence File : E:\DK\YJZ\data\20210715\YJZ1901DEF_LC 2021-07-16 22-36-17\YJZ1901DEF_LC.S
Method : E:\DK\YJZ\data\20210715\YJZ1901DEF_LC 2021-07-16 22-36-17\70b-30a-30MIN.M (Sequence Method)
Last changed : 16/07/2021 22:36:17 by 系统

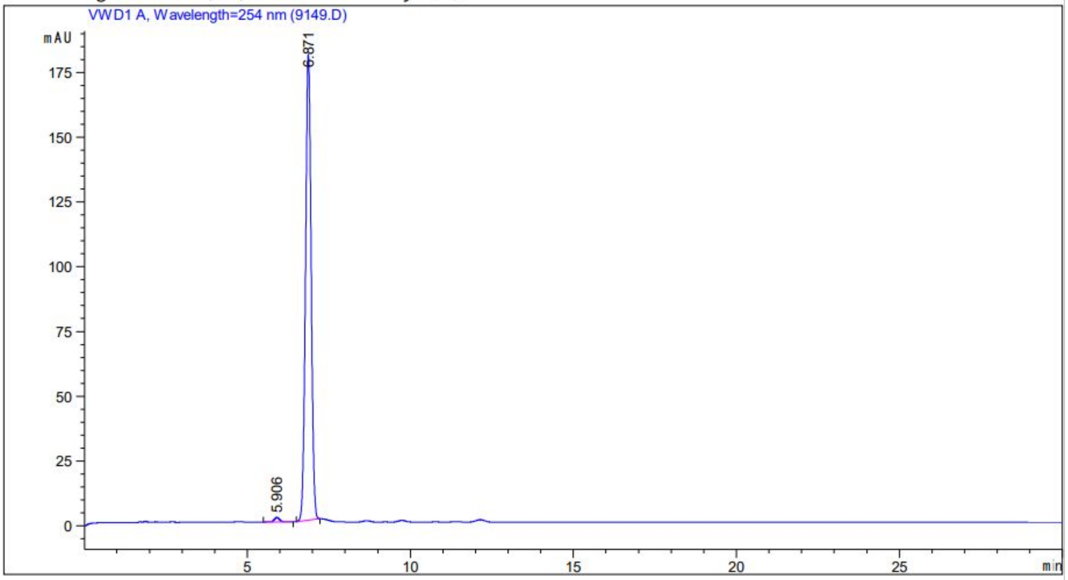

===

Area Percent Report

===

Sorted By : Signal
Multiplier : 1.0000
Dilution : 1.0000
Use Multiplier & Dilution Factor with ISTDs

Signal 1: VWD1 A, Wavelength=254 nm

Peak #	RetTime [min]	Type	Width [min]	Area [mAU*s]	Height [mAU]	Area %
1	5.906	BB	0.1804	21.08799	1.77703	0.9725
2	6.871	BB	0.1863	2147.40967	179.81970	99.0275
